# Design of Mixed Ionic-Electronic Materials for Permselective Membranes and Solid Oxide Fuel Cells Based on Their Oxygen and Hydrogen Mobility

**DOI:** 10.3390/membranes13080698

**Published:** 2023-07-27

**Authors:** Vladislav Sadykov, Elena Pikalova, Ekaterina Sadovskaya, Anna Shlyakhtina, Elena Filonova, Nikita Eremeev

**Affiliations:** 1 Federal Research Center, Boreskov Institute of Catalysis SB RAS, 630090 Novosibirsk, Russia; sadovsk@catalysis.ru (E.S.); yeremeev21@gmail.com (N.E.); 2 Institute of High Temperature Electrochemistry UB RAS, 620137 Yekaterinburg, Russia; e.pikalova@list.ru; 3 Graduate School of Economics and Management, Ural Federal University, 620002 Yekaterinburg, Russia; 4 Federal Research Center, Semenov Institute of Chemical Physics RAS, 119991 Moscow, Russia; annashl@inbox.ru; 5 Institute of Natural Sciences and Mathematics, Ural Federal University, 620002 Yekaterinburg, Russia; elena.filonova@urfu.ru

**Keywords:** solid oxide fuel cells, oxygen separation membranes, hydrogen separation membranes, oxygen mobility, hydrogen mobility, isotope exchange of oxygen

## Abstract

Oxygen and hydrogen mobility are among the important characteristics for the operation of solid oxide fuel cells, permselective membranes and many other electrochemical devices. This, along with other characteristics, enables a high-power density in solid oxide fuel cells due to reducing the electrolyte resistance and enabling the electrode processes to not be limited by the electrode-electrolyte-gas phase triple-phase boundary, as well as providing high oxygen or hydrogen permeation fluxes for membranes due to a high ambipolar conductivity. This work focuses on the oxygen and hydrogen diffusion of mixed ionic (oxide ionic or/and protonic)–electronic conducting materials for these devices, and its role in their performance. The main laws of bulk diffusion and surface exchange are highlighted. Isotope exchange techniques allow us to study these processes in detail. Ionic transport properties of conventional and state-of-the-art materials including perovskites, Ruddlesden–Popper phases, fluorites, pyrochlores, composites, etc., are reviewed.

## 1. Introduction

One of the major important challenges facing modern society is the necessity to search for renewable and environmentally friendly energy sources [1,2,3,4,5,6]. The progressive trend in alternative energy research is directed towards the intensive development of hybrid configuration systems that combine multiple energy sources and power systems to maximize the efficiency of energy production, use and storage [7,8,9,10,11,12]. Current polygeneration technologies include, among others, the combination of the solid oxide fuel cell (SOFC) as a promising source of direct energy production [13,14,15,16] with batteries, gas turbines, vehicles, heat systems, desalination systems, and hydrogen production systems [17,18,19,20,21,22,23]. The hybrid systems of SOFCs with proton-exchange membrane fuel cells [18,20,24] and waste-to-energy systems based on biofuels [25,26] should be mentioned separately.

The environmentally and economically efficient production of hydrogen and syngas for the above-mentioned polygeneration systems [27] and the dominant strategy to reduce the operating temperature of SOFCs [28] require the development of high-performance construction materials. They can be used for the design of electrodes [29,30,31,32,33,34,35] and electrolytes [36,37,38,39,40,41] in SOFCs and for the design of gas separation membranes [42,43,44,45,46] as well as in catalytic membrane reactors [47,48,49]. Oxide materials with mixed ionic-electronic conductivity (or mixed ionic electron conductors, MIECs) are considered as a prospective class of multifunctional materials and are widely investigated for their use in both SOFCs and membrane reactors [50,51,52,53,54,55,56].

Adler et al. [57] and Sadykov et al. [58] showed that the performance of SOFCs and permselective membranes, both based on MIECs, correlates with the oxygen mobility and surface reactivity, which can be characterized by the values of the oxygen self-diffusion coefficient and surface exchange constant. According to the Adler–Lane–Steele model, the electrode activity is defined both by the values of the oxygen self-diffusion coefficient and the surface exchange constant of the electrode material and by the microstructure characteristics [57,59]. In contrast to cathodes made of materials with dominant electronic conductivity, whose performance is limited by the triple-phase boundary (TPB), the performance of MIEC cathodes is limited by the double-phase boundary (DPB). It stimulates the oxygen reduction reaction at the cathode and enhances ion transport to the electrolyte [58,60,61,62].

Materials with mixed ionic-electronic conductivity are successfully applied in the design of oxygen separation membranes in catalytic membrane reactors to separate oxygen from air, while the oxygen is used in the oxidation of biofuels (methane, ethanol, etc.) to produce syngas as fuel for SOFCs. An asymmetric configuration of the membrane design includes a porous substrate and a gas-tight permselective layer, thus ensuring increased mechanical strength combined with a high oxygen flux [58]. High oxygen fluxes across the membrane and superior performance in catalytic reactions could be achieved if the materials used for the oxygen separation membranes (or for the permselective layer in the case of the asymmetric configuration) have a high mixed ionic-electronic conductivity [56,59].

The unique review work by Manthiram et al. [63] provides a comprehensive overview of the correlations between the chemical composition, crystal structure, transport properties and electrochemical performance of the mixed ionic-electronic oxide conductors that could be used in both SOFCs and membrane reactors. Oxides with the perovskite structure ABO_3_, layered perovskites LnBaCo_2_O_5+δ_, Ruddlesden–Popper phases (La,Sr)_n+1_M_n_O_3n+1_ and hexagonal phases RBa(Co_1−y_M_y_)_4_O_7_ have been widely discussed in the review. Meanwhile, it is worth noting that review [63] was published in 2011.

**Figure 1:**
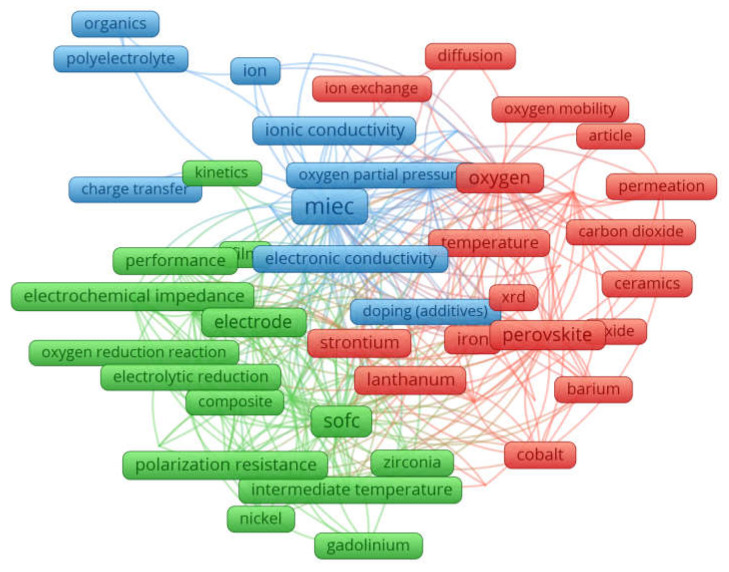
Thematic map of co-occurring author keywords from search results for the keyword {mixed ionic electron conductor (MIEC)} in the Scopus database.

A global search in the scientometric database Scopus with the query {mixed ionic electron conductor} yielded 535 documents of the type of Article and Review, indexed in the period from 1986 to June 2023. Figure 1, constructed with the software package VOSviewer version 1.6.19 [64] considering a minimum number of occurrences equal to 20 author keywords, visualizes the map with thematic clusters related to MIECs according to the query {mixed ionic electron conductor}.

The graphical data shown in Figure 1 illustrate that the author keywords in the documents referring to MIECs can be divided into three thematic clusters: the green cluster focusing on SOFCs, the red cluster focusing on oxygen membranes, and the blue cluster focusing on electrical conductivity. It can be said that the blue cluster highlights the fundamental property of MIECs—the presence of ionic and electronic conductivity—while the red and green clusters characterize the applications of MIECs. The green cluster summarizes the electrochemical activity of MIECs as the anode, cathode, including composite, and electrolyte materials, and the electrochemical performance of MIEC-based SOFCs in general. The red cluster generalizes the topics related to oxygen transport in MIEC-based membranes: oxygen mobility, oxygen permeability, oxygen diffusion, surface reactivity and ion exchange. Thus, published documents on the mixed ionic-electronic conductors can, therefore, be grouped under the three research themes mentioned above.

Among the highly cited documents in the MIEC search list in the Scopus database, the reviews highlighting the applications of MIECs as the anode [65,66] and cathode [67,68] materials for SOFCs and as the ceramic separation membranes [50,51,52] were found. However, it is worth noting that all the above reviews were published up to the year 2017. Therefore, the aim of the present review is to summarize recent studies on the oxide materials with mixed ionic-electronic conductivity, such as perovskites, fluorites, Ruddlesden–Popper phases, pyrochlores, and composites, focusing on their ion transport properties: oxygen and hydrogen diffusion, oxygen and hydrogen isotope exchange, and oxygen and hydrogen mobility. The applications of MIECs in SOFCs as cathodes and as the functional layers of the oxygen separation membranes are also highlighted.

## 2. Importance of Oxygen and Hydrogen Transport Properties for the Performance of Membranes and SOFCs

### 2.1. Oxygen Separation Membranes

**Figure 2:**
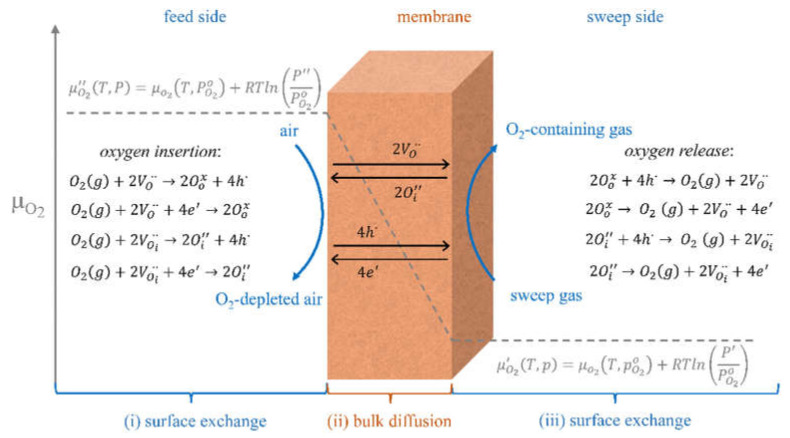
Schematic diagram of the different sections involved in the oxygen transport during oxygen permeation [74]. Reprinted with permission from Ref. [74]. Copyright 2019 Elsevier.

High oxygen mobility and surface reactivity as well as a high electronic conductivity are the crucial characteristics of oxygen separation membrane materials required for achieving high oxygen permeation fluxes. The oxygen bulk diffusion enables oxide ions’ transport across the membrane, while the oxygen surface exchange enables oxygen adsorption/desorption. Since the oxide ions’ transport across the membrane is coupled with the electron transport, a high electronic conductivity is required as well (Figure 2). This allows to use such membranes for pure oxygen production as well as a part of catalytic membrane reactors for fuels transformation reactions [53,55,58,69,70,71,72,73,74,75,76].

The oxygen permeation flux across the membrane $\left(j_{O_{2}}\right)$ obeys the Wagner equation (Equation (1)):

(1) $$j_{O_{2}}=-\frac{RT}{16F^{2}L}\int_{\ln P_{O_{2}}^{I}}^{\ln P_{O_{2}}^{II}}\frac{\sigma_{O}\sigma_{el}}{\sigma_{O}+\sigma_{el}}d\ln P_{O_{2}},$$

where *F* is the Faraday constant, *L* is the membrane thickness, $P_{O_{2}}^{I}$ and $P_{O_{2}}^{II}$ are the oxygen partial pressures at different sites of the membrane, and *σ_O_* and *σ_el_* are oxide-ionic and electronic conductivity, respectively [70]. In MIEC materials, *σ_O_* << *σ_el_*. If the ionic conductivity is constant across the entire membrane thickness, Equation (1) can be simplified as follows (Equation (2)):

(2) $$j_{O_{2}}≅-\frac{RT}{16F^{2}L}\sigma_{O}\ln\frac{P_{O_{2}}^{II}}{P_{O_{2}}^{I}}.$$

For MIEC membrane materials with oxygen nonstoichiometry depending on the oxygen partial pressure proportional to $P_{O_{2}}^{n}$, the Nernst–Einstein equation of their ionic conductivity (Section 3) can be re-written as follows (Equation (3)):

(3) $$\sigma_{O}=\frac{4F^{2}}{RTV_{m}}D_{V}\delta_{0}P_{O_{2}}^{n},$$

where *δ*_0_ is the oxygen nonstoichiometry at the reference oxygen pressure (1 atm), *V_m_* is the molar volume of the oxide, *D_V_* is the oxygen vacancy’s self-diffusion coefficient. Combining this with Equation (1) and assuming *σ_O_* << *σ_el_* one can obtain Sievert’s law (Equation (4)),

(4) $$j_{O_{2}}=-\frac{D_{V}\delta_{0}}{4V_{m}L}\int_{\ln P_{O_{2}}^{I}}^{\ln P_{O_{2}}^{II}}P_{O_{2}}^{n}d\ln P_{O_{2}}=\left(\frac{A}{L}\right)\left({\left(P_{O_{2}}^{I}\right)}^{n}-{\left(P_{O_{2}}^{II}\right)}^{n}\right),$$

where $A=\frac{D_{V}\delta_{0}}{4V_{m}n}.$

Considering the effect of the surface exchange of oxygen, the Wagner Equation (2) is transformed into the modified Wagner equation introduced by Bouwmeester et al. (Equation (5)) [77]:

(5) $$j_{O_{2}}=\frac{1}{1+\frac{2L_{C}}{L}}-\frac{RT}{16F^{2}L}\sigma_{O}\ln\frac{P_{O_{2}}^{II}}{P_{O_{2}}^{I}}.$$

where *L_C_* is the characteristic thickness (will be defined in Section 3).

**Figure 3:**
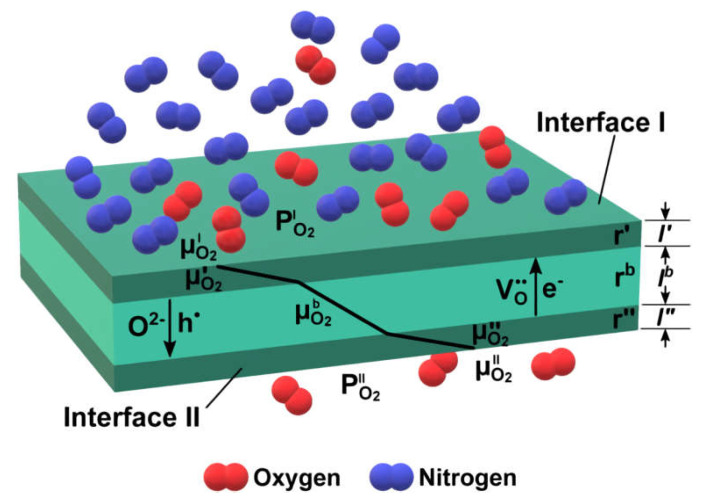
Conceptualization of oxygen permeation process according to the Zhu model [75]. Reprinted with permission from Ref. [75]. Copyright 2019 Elsevier.

Several models are used to model the membrane performance based on the membrane material oxygen mobility and surface reactivity, electronic conductivity, and other characteristics, such as Jacobson’s model [70], Xu and Thomson’s model [70,78], Zhu’s model [70,75,76]. E.g., Zhu’s model (Figure 3) is based on the Wagner equation and considers the area-specific resistance of membrane surfaces at the air and purge sides (*r′* and *r″*, respectively), which are proportional to the reciprocal oxygen surface exchange constant, and the membrane bulk (*r^b^*), which is proportional to the reciprocal oxygen self-diffusion coefficient.

In the case of a multi-layer asymmetric supported membrane, the characteristics of each layer should be considered along with the properties of gas-phase diffusion in a porous support [55,58,79,80,81]. However, gas-phase phenomena are out of the scope of this review.

### 2.2. Hydrogen Separation Membranes

Similar to the oxygen separation membranes, a high hydrogen mobility and surface reactivity as well as a high electronic conductivity are required for hydrogen separation membrane materials. This allows it to reach high hydrogen permeation fluxes for obtaining pure hydrogen including its production in catalytic membrane reactors for fuel transformation reactions [42,55,58,82,83,84,85,86,87,88,89]. There are advantages in using triple (H^+^/O^2−^/e^−^)-conducting materials for hydrogen separation membranes, since the presence of the oxide-ionic component of the conductivity can enable the following features:

1. Some proton transport mechanisms being mediated by the oxygen transport as will be mentioned in Section 3.2 [90,91,92];
2. Oxide ion counterpermeation across the membrane allows us to increase the hydrogen yield due to the water splitting reaction [89,93,94];
3. Triple conductivity allows to enhance the performance in various catalytic reactions and to improve gas separation properties due to the coupled transport of all types of mobile species, forcing them to be transported against their chemical potential gradient [95,96,97].

**Figure 4:**
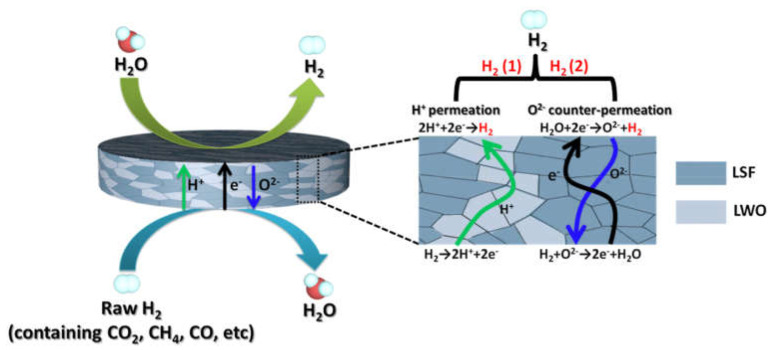
Schematic diagram of hydrogen production by La_5.5_WO_11.25−δ_-La_0.8_Sr_0.2_FeO_3−δ_ (LWO-LSF) mixed triple-conducting membrane with H^+^ permeation and O^2−^ counter-permeation property [89]. Reprinted with permission from Ref. [89]. Copyright 2021 Elsevier.

The processes in the triple-conductive hydrogen separation membrane are illustrated in Figure 4.

For dense metallic membranes, the hydrogen concentration in metal is proportional to $P_{H_{2}}^{0.5}$ [88]. Similar to MIEC oxides with the variation of oxygen nonstoichiometry on the oxygen partial pressure (Equation (4)), Sievert’s law (Equations (6) and (7)) can be obtained:

(6) $$j_{H_{2}}=\left(\frac{Pe}{L}\right)\left({\left(P_{H_{2}}^{I}\right)}^{n}-{\left(P_{H_{2}}^{II}\right)}^{n}\right),$$

where

(7) $$Pe=0.5D_{H}K_{s}$$

is the hydrogen permeability, $P_{H_{2}}^{I}$ and $P_{H_{2}}^{II}$ are hydrogen partial pressures in retentate and permeate gases, respectively, *n* is the exponent which in the ideal case is equal to 0.5 (for real membranes it lies in the range of ~0.5–1), *D_H_* is the hydrogen self-diffusion coefficient, and *K_s_* is the hydrogen solubility constant (Sievert’s constant) [87,88,98].

For ceramic membranes containing only protonic-electronic conductors, the Wagner equation can be written as follows (Equation (8)):

(8) $$j_{H_{2}}=-\frac{RT}{4F^{2}L}\int_{\ln P_{H_{2}}^{I}}^{\ln P_{H_{2}}^{II}}\frac{\sigma_{H}\sigma_{el}}{\sigma_{H}+\sigma_{el}}d\ln P_{H_{2}},$$

where *σ_H_* is the protonic conductivity [87,99,100]. Since protonic and electronic conductivity may depend on $P_{H_{2}}$, the result of integrating it in Equation (8) can be different. Assuming *σ_H_* << *σ_el_* and *σ_H_* is proportional to $P_{H_{2}}^{n}$, there are limiting cases which can be considered:

- *n* = 0.5, when protons are minority defects, then $j_{H_{2}}=-\frac{RT}{2F^{2}L}\sigma_{H,0}\left({\left(P_{H_{2}}^{I}\right)}^{0.5}-{\left(P_{H_{2}}^{II}\right)}^{0.5}\right)$;

- *n* = 0.25, when protons are majority defects compensated by electrons, then $j_{H_{2}}=-\frac{RT}{F^{2}L}\sigma_{H,0}\left({\left(P_{H_{2}}^{I}\right)}^{0.25}-{\left(P_{H_{2}}^{II}\right)}^{0.25}\right);$

- *n* = 0, when protons are majority defects compensated by acceptor dopants, then $j_{H_{2}}=-\frac{RT}{4F^{2}L}\sigma_{H,0}\ln\left(\frac{P_{H_{2}}^{II}}{P_{H_{2}}^{I}}\right)$ [87,99,101,102,103].

In the case of a cermet membrane, the equation for its hydrogen permeation flux combines those for the ceramic (Equation (8)) and metallic (Equation (6)) components (Equation (9)):

(9) $$j_{H_{2}}=-\left(x_{ceram}\frac{RT}{4F^{2}L}\int_{\ln P_{H_{2}}^{I}}^{\ln P_{H_{2}}^{II}}\frac{\sigma_{H}\sigma_{el}}{\sigma_{H}+\sigma_{el}}d\ln P_{H_{2}}+\left(1-x_{ceram}\right)\left(\frac{Pe_{metal}}{L}\right)\left({\left(P_{H_{2}}^{I}\right)}^{n}-{\left(P_{H_{2}}^{II}\right)}^{n}\right)\right),$$

where *x_ceram_* is the volume fraction of the ceramic component, *Pe_metal_* is the permeability of the metallic component [86].

For triple-conductive membranes, the oxide-ionic component of the conductivity should be accounted for (Equations (10)–(12)) [89,103]:

(10) $$j_{H_{2}}=-\frac{RT}{8F^{2}L}\int_{I}^{II}\sigma_{H}\left(2\frac{\sigma_{O}+\sigma_{el}}{\sigma_{H}+\sigma_{O}+\sigma_{el}}d\ln P_{H_{2}}+\frac{\sigma_{O}}{\sigma_{H}+\sigma_{O}+\sigma_{el}}d\ln P_{O_{2}}\right),$$

(11) $$j_{H_{2}}=-\frac{RT}{4F^{2}L}\int_{I}^{II}\sigma_{H}\left(\frac{\sigma_{el}}{\sigma_{H}+\sigma_{O}+\sigma_{el}}d\ln P_{H_{2}}+\frac{\sigma_{O}}{\sigma_{H}+\sigma_{O}+\sigma_{el}}d\ln P_{H_{2}O}\right),$$

(12) $$j_{H_{2}}=\frac{RT}{4F^{2}L}\frac{\sigma_{H}+\sigma_{el}}{\sigma_{H}+\sigma_{O}+\sigma_{el}}\ln\frac{P_{H_{2}}^{I}}{P_{H_{2}}^{II}}+\frac{RT}{8F^{2}L}\frac{\sigma_{O}+\sigma_{el}}{\sigma_{H}+\sigma_{O}+\sigma_{el}}\ln\frac{P_{O_{2}}^{II}}{P_{O_{2}}^{I}}.$$

In the case of the asymmetric supported hydrogen separation membrane, more complex description is required since mass and heat transfer phenomena take place in the gas phase in the layers of the porous support. Gas-phase mass transport certainly affects the membrane performance or even can determine its characteristics [55,99,104,105,106]. However, gas-phase phenomena are out of the scope of this review.

### 2.3. Solid Oxide Fuel Cells

**Figure 5:**
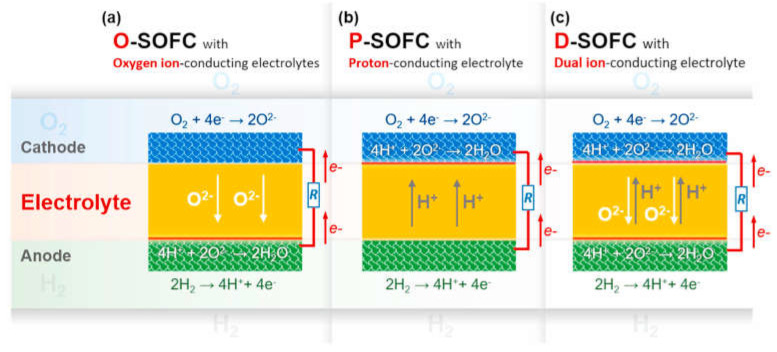
Schematic diagrams of the working principles for (**a**) O-SOFC, (**b**) P-SOFC and (**c**) D-SOFC [109]. Reprinted with permission from Ref. [109]. Copyright 2020 Elsevier.

By selecting solid oxide fuel cell materials with a high oxygen and/or hydrogen mobility, the fuel cell operating temperature can be lowered while maintaining or even increasing the power output. A high oxide-ionic or/and protonic conductivity of the electrolyte reduces its resistance which is a predominant component of the ohmic losses of the cell [41,53,58,69,107,108,109]. Figure 5 demonstrates SOFCs with oxide-ionic, protonic, and dual (oxide-ionic + protonic) conductive electrolytes.

**Figure 6:**
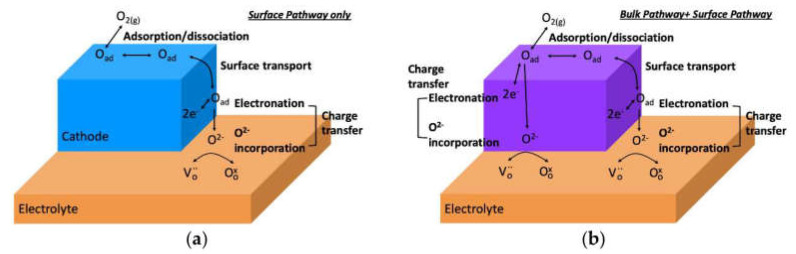
Schematic of possible elementary reaction steps during oxygen reduction reaction (ORR) and possible pathways for two different classes of cathode materials; (**a**) pure electronic conductor and (**b**) mixed ionic and electronic conducting (MIEC) cathodes [110]. Reprinted from Ref. [110] under the CC BY 4.0 license.

Using the electrode materials with pure electronic conductivity leads to limiting the electrode process by the electrode–electrolyte–gas phase triple-phase boundary (TPB) (Figure 6a). The ionic (oxide-ionic or/and protonic) component of the conductivity allows the electrode process to take place on the electrode–gas phase double-phase boundary (DPB) (Figure 6b). This results in the improvement in the electrode reaction kinetics [53,55,56,58,65,66,67,68,69,108,110].

## 3. Oxygen and Hydrogen Diffusion in MIEC Materials

### 3.1. Self-Diffusion, Tracer Diffusion and Chemical Diffusion

Let us consider an oxide-based material, in which the following species are mobile: electrons/holes, oxide anions/oxygen vacancies, protons/hydroxyls, etc. The flux of each of these species is ${\vec{j}}_{i}$. In the absence of the gradients of electric potential and temperature, the Fick’s first law (Equation (13)) is satisfied:

(13) $${\left.\vec{j_{i}}\right|}_{\vec{\nabla }T=0}=-D_{i}\vec{\nabla }C_{i},$$

where *D_i_* is a diffusion coefficient of *i*-th species, *C_i_* is their concentration. Strictly speaking, Equation (13) is correct if the diffusing species do not interact with each other. The interaction of the following species in triple-conductive oxides: holes, oxygen vacancies and protons, was noted in the number of studies [111,112,113]. In this case, in a linear non-equilibrium thermodynamics region, Equation (13) can be written as follows (Equation (14)):

(14) $${\left.\vec{j_{i}}\right|}_{\vec{\nabla }T=0}=-\sum_{k}\sum_{l}D_{k(l)}^{i}\vec{\nabla }C_{l},$$

where the coefficients $D_{k(l)}^{i}$ correspond to the effect of the *l*-th species concentration gradient, $\vec{\nabla }C_{l}$, on the *i*-th species diffusive flux, ${\vec{j}}_{i}$, and such coefficients are the sums of the respective species’ diffusion coefficients multiplied by the transference numbers. Three types of the $D_{k(l)}^{i}$ coefficients can be distinguished:

- “Direct” coefficients $D_{i(l)}^{i}$ corresponding to the effect of the gradient $\vec{\nabla }C_{i}$ on the flux ${\vec{j}}_{i}$;

- “Indirect” coefficients $D_{l(i)}^{i}$ corresponding to the effective diffusion coefficients of *i*-th species under the effect of the driving force $\vec{\nabla }C_{l}$ when $\vec{\nabla }C_{i}=0$, i.e., they correspond to the effect of the gradient $\vec{\nabla }C_{l}$ on the flux ${\vec{j}}_{i}$ when $\vec{\nabla }C_{i}=0$;

- The coefficients with three different indices $D_{k(l)}^{i}$ correspond to the effective diffusion coefficients of *i*-th species when $\vec{\nabla }C_{k}$ is a driven force and $\vec{\nabla }C_{l}=0$.

Fick’s second law (Equation (15)) follows from Fick’s first law and the mass conservation:

(15) $$\frac{\partial C_{i}}{\partial t}=D_{i}\hat{\Delta }C_{i}.$$

In the absence of the chemical potential gradient, *D_i_* is referred to as a self-diffusion coefficient. It is related to the ionic conductivity (*σ_i_*) according to the Nernst–Einstein equation (Equation (16)) [114,115,116,117]:

(16) $$\sigma_{i}=\frac{f_{I,i}D_{i}C_{i}q_{i}}{k_{B}T},$$

where *f_I,i_* is a correlation factor, *f_I,i_* ≈ 1. The self-diffusion coefficient is related to the tracer diffusion coefficient $\left(D_{i}^{*}\right)$ determined by isotope exchange techniques as follows (Equation (17)):

(17) $$D_{i}^{*}=f_{i}D_{i},$$

where *f_i_* is a correlation factor which is related to influence of counterflows of ions of different isotopes [69,114,116,117]. The ratio (Equation (18))

(18) $$H_{R}=\frac{f}{f_{I}}$$

is referred to as a Haven ratio.

It is to be noted that *i*-th species’ mobility may be non-uniform in the material’s bulk: the fraction *θ*_1_ of these species possesses a self-diffusion coefficient of *D_i_*_1_, the fraction *θ*_2_ possesses a self-diffusion coefficient of *D_i_*_2_, etc. [69,118,119,120,121,122,123]. In this case, the transport of the *i*-th species’ can be described by a set of parameters {*D_ij_*(*T*), *θ_j_*} or by an effective (mean) self-diffusion coefficient (Equation (19)):

(19) $$D_{over,i}=\sum_{j}\theta_{j}D_{ij}.$$

If one of *D_ij_* significantly exceeds other self-diffusion coefficients (it can be denoted as *D_i,fast_*) and its fraction *θ_j_* is high enough (it can be denoted as *θ_fast_*), then *D_over,i_* ≈ *θ_fast_ D_i,fast_*.

However, in the real operating conditions of SOFCs/SOECs and permselective membranes, the chemical or electrochemical potential gradient occurs due to different gas phase composition in various device compartments and the flowing electric current. In this case, instead of the Fick’s first law (Equation (13)), the Nernst–Plank equation (Equation (20)) [124,125,126]

(20) $${\left.\vec{j_{i}}\right|}_{\vec{\nabla }T=0}=-D_{i}\vec{\nabla }C_{i}-\frac{D_{i}C_{i}q_{i}}{k_{B}T}\vec{\nabla }\varphi$$

or the modified Fick’s first law (Equation (21)) [117,124,127]

(21) $${\left.\vec{j_{i}}\right|}_{\vec{\nabla }T=0}=-\Gamma_{V}D_{i}\vec{\nabla }C_{i}$$

can be used, where Γ*_V_* is referred to as the thermodynamic factor, or in other words, the factor of enhancement. In this case, such a gradient as a driving force (as well as electroneutrality conservation) causes net transport of species characterized by a chemical diffusion coefficient (*D_chem_*), which is related to the self- and tracer diffusion coefficients as follows (Equation (22)) [69,114,117]:

(22) $$D_{chem}=\Gamma_{V}D_{i}=\frac{\Gamma_{V}}{H_{R}}D_{i}^{*}.$$

For oxide-ionic and mixed oxide-ionic–electronic conductors, the following equations for the thermodynamic factor (Equations (23) and (24)) are known:

(23) $$\Gamma_{V}=-t_{h}\cdot \frac{1}{2}\frac{\partial \ln P_{O_{2}}}{\partial \ln C_{V_{O}^{••}}},$$

(24) $$\Gamma_{V}=t_{h}\left(1+\frac{4C_{V_{O}^{••}}}{C_{h}}\right),$$

where *t_h_* is the hole transport number, $P_{O_{2}}$ is the partial pressure of oxygen in the gas phase, and $C_{V_{O}^{••}}$ and *C*_h_ are the concentrations of oxygen vacancies and holes in the oxide, respectively [69,117,127,128,129,130].

For protonic conductors, a more complex relationship of chemical diffusion coefficient and self-diffusion coefficients of charge carriers (Equation (25)) is given in the work [111]:

(25) $$D_{chem}=\frac{\left(2-\frac{C_{{\mathrm{OH}}_{O}^{•}}}{2C_{{\left.V_{O}^{••}\right|}_{P_{{\;H}_{2}O}=0}}}\right)D_{H}D_{V}}{\frac{C_{{\mathrm{OH}}_{O}^{•}}}{2C_{{\left.V_{O}^{••}\right|}_{P_{{\;H}_{2}O}=0}}}D_{H}+2\left(1-\frac{C_{{\mathrm{OH}}_{O}^{•}}}{2C_{{\left.V_{O}^{••}\right|}_{P_{{\;H}_{2}O}=0}}}\right)D_{V}},$$

where $C_{{\mathrm{OH}}_{O}^{•}}$ is the concentration of hydroxyl ions in the oxide, $P_{H_{2}O}$ is the partial pressure of water in the gas phase, *D_H_* is the self-diffusion coefficient of protons, *D_V_* is the self-diffusion coefficient of oxygen vacancies (Equation (26)),

(26) $$D_{V}=\frac{C_{O}}{C_{V_{O}^{••}}}D_{O}=\frac{1}{f_{O}}\frac{C_{O}}{C_{V_{O}^{••}}}D_{O}^{*}.$$

For oxide-ionic and mixed oxide-ionic–electronic conductors, the temperature dependence of the oxygen self-diffusion coefficient is given according to the random walk theory (Equation (27)):

(27) $$D_{O}=\frac{\zeta }{6}\varepsilon^{2}C_{V_{O}^{••}}\nu \times \exp\left(\frac{\Delta_{m}S}{R}\right)\times \exp\left(-\frac{\Delta_{m}H}{RT}\right),$$

where *ζ* is a number of equivalent positions, *ε* is the random walk step length, *ν* is a frequency of particle vibrations, Δ*_m_S* and Δ*_m_H* are migration entropy and enthalpy, respectively [131].

For the self-diffusion coefficient of protons in interstitial sites of a metal face-centered cubic lattice with the parameter *a* (e.g., nickel), the following equation (Equation (28)) is given in the work [132]:

(28) $$D_{H}=a^{2}\frac{k_{B}T}{h}\exp\left(-\frac{\Delta_{TS-oxt}G}{k_{B}T}\right),$$

where *h* is the Planck constant, Δ*_TS–oct_G* is the Gibbs’ energy of the proton transition from the transition state to the ground octahedral state.

### 3.2. Oxygen and Hydrogen Diffusion Mechanisms

**Figure 7:**
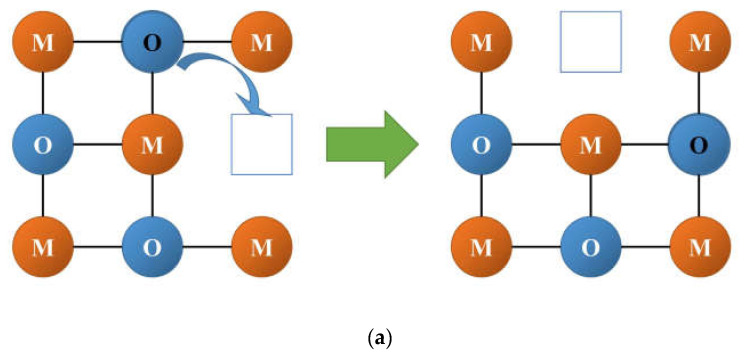

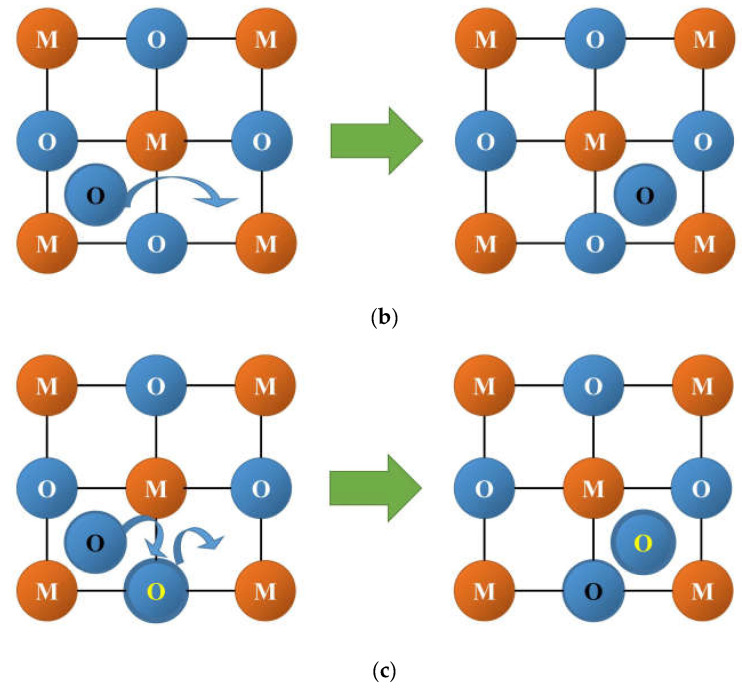
General types of oxygen diffusion mechanisms in oxides: (**a**) vacancy mechanism; (**b**) interstitial mechanism; and (**c**) cooperative mechanism [70,134,135,136].

There are three general types of oxygen diffusion mechanisms in oxides and composites:

- Vacancy mechanism (Figure 7a): transport of regular oxide anions into neighboring vacancies; this mechanism is typical for perovskites, fluorites and many other types of oxides [54,70,107,133,134];
- Interstitial mechanism (Figure 7b): transport of interstitial oxide anions into neighboring interstitial sites; this mechanism is typical for some pyrochlores, mayenites and some other oxides [70,107,134,135,136,137];
- Cooperative mechanism (Figure 7c): cooperative movement of different types of oxide anions (regular, interstitial); this mechanism is typical for Ruddlesden–Popper phases, apatites, brownmillerites, orthorhombic oxides and is proposed for some other oxides [69,70,107,134,135,136,138].

In the case of non-uniformity of the oxygen mobility in the materials’ bulk due to structural and defect features, more complex features of oxygen transport can take place. They will be reviewed in details in Section 6.

**Figure 8:**
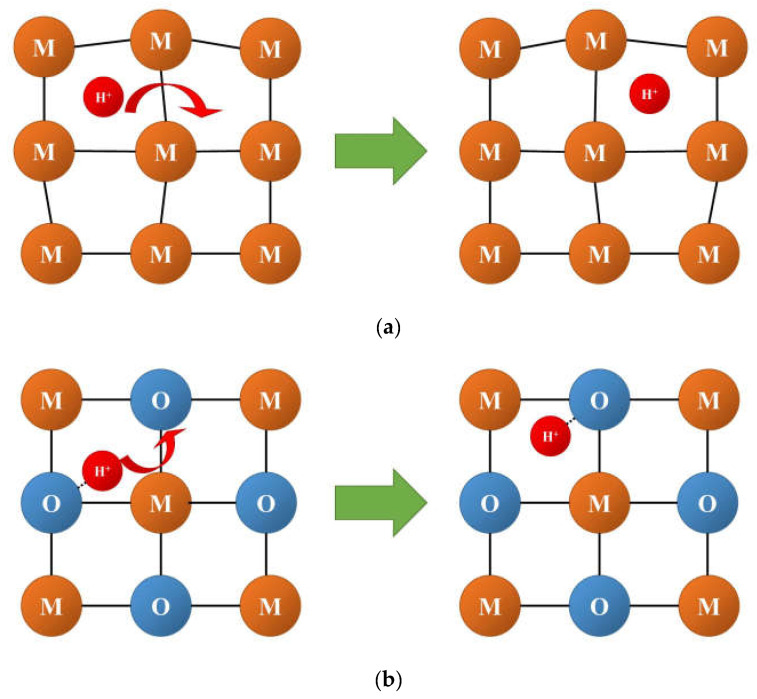

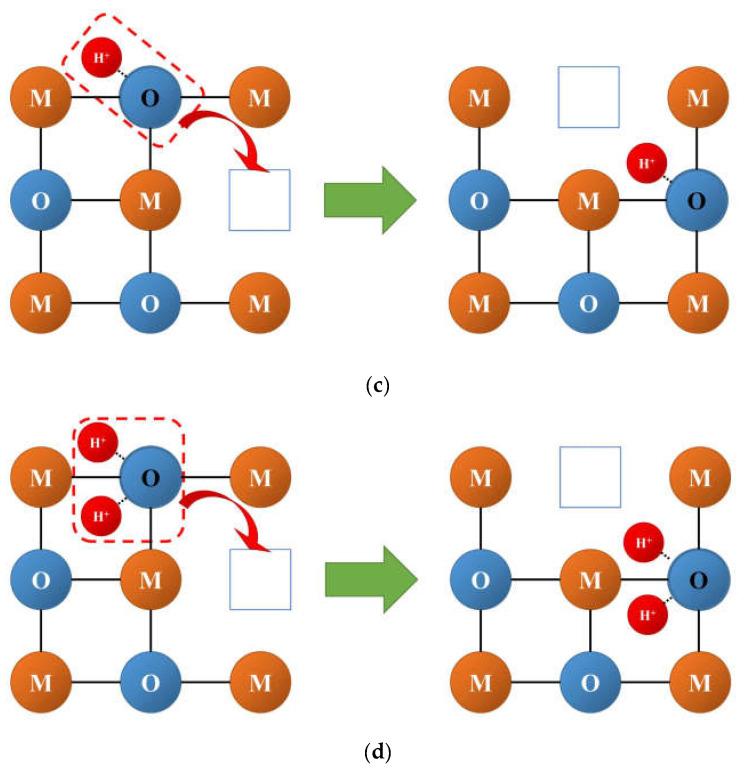
General types of hydrogen diffusion mechanisms: (**a**) diffusion of interstitial protons; (**b**) Grotthuss mechanism; (**c**) vehicle mechanism; (**d**) diffusion of structurally bound water [90,91,92].

The main mechanisms of hydrogen diffusion are:

- Diffusion of protons through interstitial defects (Figure 8a); this mechanism is typical for metals and alloys [90,91,99,132,139];
- Grotthuss mechanism (Figure 8b): jumps of protons between neighboring oxide anions with reorientation of M–O–H bonds; this mechanism is typical for the most oxides possessing a protonic component of conductivity [91,111,140];
- Vehicle mechanism (Figure 8c): transport of protons together with the neighboring oxide anion as a hydroxyl; this mechanism is also typical for proton-conducting oxides [91,111,140];
- Diffusion of structurally bound water (Figure 8d): transport of water species embedded into the lattice; this mechanism is proposed for some oxides [92,141].

### 3.3. Surface Exchange of Oxygen and Hydrogen

In 1970s, V.S. Muzykantov demonstrated that the interaction of oxide-based materials with molecular oxygen takes place via a dissociative adsorption–desorption mechanism (Muzykantov–Boreskov mechanism) [142,143], which includes the following stages:

1. Physical adsorption;
2. Dissociative chemisorption (Equations (29)–(31)):

(29) $${\;O}_{2\left(g\right)}+2{\left(\right)}_{a}=2{\left(O\right)}_{a},$$

(30) $${\;O}_{2\left(g\right)}+{\left(\right)}_{a}+{\left[\right]}_{S}={\left(O\right)}_{a}+{\left[O\right]}_{S},$$

(31) $${\;O}_{2\left(g\right)}+2{\left[\right]}_{S}=2{\left[O\right]}_{S};$$

3. Embedding (the exchange itself) (Equation (32)):

(32) $${\left(O\right)}_{a}+{\left[\right]}_{S}={\left(\right)}_{a}+{\left[O\right]}_{S}.$$

Here, ( )_a_ and [ ]_S_ denote the adsorption site and oxygen vacancy on the surface, respectively, (O)_a_ and [O]_S_ are the adsorbed (weakly bound, capable of surface diffusion) and surface (strongly bound with oxygen vacancy) oxygen species, respectively.

The exchange of oxygen with carbon dioxide proceeds in a different way compared to that with molecular oxygen. First, the entire surface of the material participates in the exchange, not only the active sites. Secondly, the mechanism of exchange is different. As a result, the exchange with CO_2_ proceeds 2–5 orders of magnitude faster compared to the exchange with O_2_. The CO_2_ molecule is adsorbed on the surface of the oxide (Equation (33)):

(33) $${\mathrm{CO}}_{2\left(g\right)}+{\left(\right)}_{a}={\left({\mathrm{CO}}_{2}\right)}_{a},$$

then interacts with a neighboring oxide anion to form the carbonate complex (Equation (34)):

(34) $${\left({\mathrm{CO}}_{2}\right)}_{a}+\left[O\right]={\left[{\mathrm{CO}}_{3}\right]}_{S},$$

or with a neighboring oxygen vacancy to form the carboxylate complex (Equation (35)) [144,145,146,147,148,149,150]:

(35) $${\left({\mathrm{CO}}_{2}\right)}_{a}+\left[\right]={\left[{\mathrm{CO}}_{2}\right]}_{S},$$

or, especially at high temperatures, can dissociate to the adsorbed carbon monoxide molecule and adsorbed oxygen (Equation (36)) [150,151,152,153]:

(36) $${\left({\mathrm{CO}}_{2}\right)}_{a}+{\left(\right)}_{a}={\left(\mathrm{CO}\right)}_{a}+{\left(O\right)}_{a}.$$

**Figure 9:**
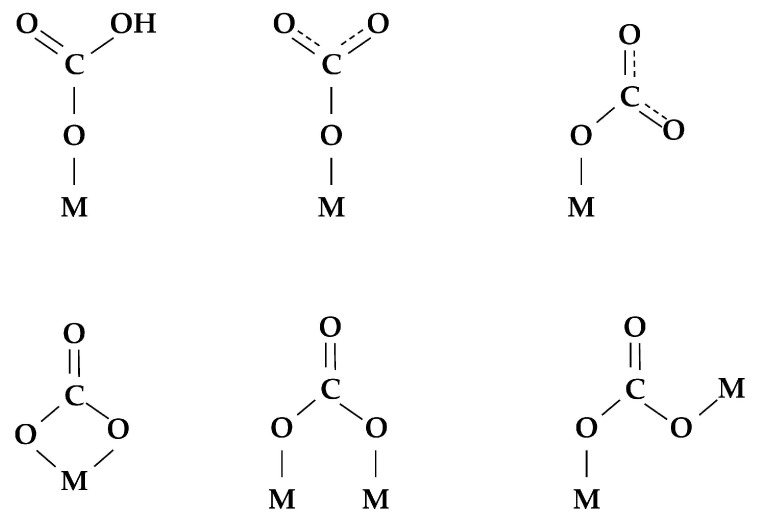
The examples of surface carbonate complexes [145,149,153].

The carbonate complexes can be negatively charged complexes like ${\mathrm{CO}}_{3}^{2-}$, ${\mathrm{CO}}_{3}^{-}$, neutrally charged complexes like CO_3_, or complexes being something between CO_3_ and CO_2_ as well. They can have various configurations depending on the metal cation they are coordinated to. The examples of such complexes are given in Figure 9 [144,145,149,154].

**Figure 10:**
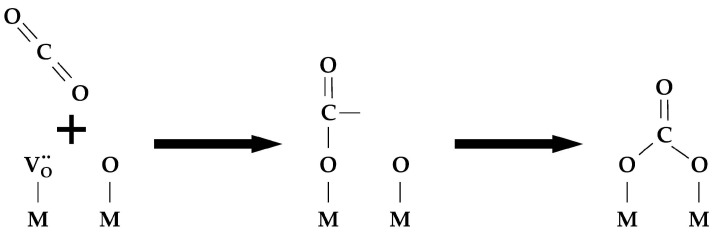
The formation of surface carbonate ion while closing of carboxylate ion-radical’s carbon bond on the oxide surface oxygen [149].

The carboxylate ion-radical, formed by the interaction of the adsorbed CO_2_ molecule and the oxygen vacancy, is unstable, and the free valence of carbon would tend to be saturated (Figure 10) [149].

The interaction of molecular hydrogen with metals is described by two main mechanisms: the Bonhoeffer–Farkas mechanism (Equation (37)) [155] corresponding to the dissociative adsorption of hydrogen with the formation of hydrogen adatoms H_a_:

(37) $$H_{2}=2H_{a},$$

and the Eley–Rideal mechanism (Equation (38)) [156,157], for which the formation of a hydrogen adatom on the metal surface proceeds through the stage of the formation of a three-atomic activated complex ${\left(H\cdots H\cdots H\right)}_{a}$:

(38) $$H_{2}+H_{a}={\left(H\cdots H\cdots H\right)}_{a}.$$

For metals which can intercalate hydrogen in their structure as interstitial defects (H*_i_*; Figure 8a), the intercalation reaction (Equation (39)) is considered as well [90,91]:

(39) $$H_{a}=H_{i}.$$

Oxide materials interact with molecular hydrogen via its adsorption, dissociation, and interaction with the surface oxygen. They interact with water via hydration. In both cases, hydroxyls are formed on the oxide surface. These reactions are given in Equations (40)–(44) [99,111,112,113,139].

(40) $$H_{2}=2H^{•}+2\;e^{\prime },$$

(41) $$H_{2}+2O_{O}^{\times }=2{\mathrm{OH}}_{O}^{•}+2\;e^{\prime },$$

(42) $$H_{2}O+2h^{•}=2H^{•}+\frac{1}{2}O_{2},$$

(43) $$H_{2}O+V_{O}^{••}=2H^{•}+O_{O}^{\times },$$

(44) $$H_{2}O+O_{O}^{\times }+V_{O}^{••}=2{\mathrm{OH}}_{O}^{•}.$$

The rate of surface exchange is typically determined in terms of a surface exchange constant (*k*). In the presence of a chemical potential gradient, there is a chemical surface exchange constant (*k_chem_*). In the absence of such a gradient, there is a surface exchange constant (*k_ex_* or k^*^ in the case of isotope studies) which, like diffusion coefficients, is related to the chemical surface exchange constant via a thermodynamic factor. The ratio of diffusion coefficient and surface exchange constant is referred to as a characteristic thickness (Equation (45)) [129,158]:

(45) $$L_{C}=\frac{D^{*}}{k^{*}},\ldots \ldots L_{C,chem}=\frac{D_{chem}}{k_{chem}}.$$

## 4. Isotope Exchange of Oxygen and Hydrogen

Isotope exchange techniques are based on the substitution of one isotope of the element in the sample (e.g., oxygen, hydrogen) with the other isotope (e.g., ^16^O/^18^O, H/D) while interacting with a gas-phase reagent in equilibrium or steady-state. These methods allow us to acquire the data on D^*^ and k^*^. They can be divided into methods with the solid-state-phase and gas-phase analysis. The methods with solid-state-phase analysis such as SIMS, in some cases do not allow one to study diffusion processes in detail and allow one to acquire the data on the mean integral diffusion coefficient. Therefore, the methods with the gas phase analysis are more suitable for studying oxygen and hydrogen mobility features for SOFC and permselective membrane materials [32,159]. The methods with gas phase analysis can be implemented using static or flow reactors. In the case of using flow reactors, gas-phase diffusion limitations are avoided, and more complete isotopic substitution can be achieved during the experiment, allowing oxygen and hydrogen transport features to be studied in greater detail [32,55,58,69,152,159,160,161,162,163,164]. In the authors’ previous experimental works and reviews [55,58,69,161,162,163,164], it has been demonstrated that temperature-programmed oxygen isotope exchange with ^18^O_2_ and C^18^O_2_ allows one to describe the oxygen surface-exchange mechanisms and bulk oxygen diffusion features, including the non-uniformity of bulk oxygen mobility, for many types of ceramic materials, and for SOFCs, permselective membranes and other applications. The most interesting feature of using C^18^O_2_ as a ^18^O-containing gas-phase reagent instead of ^18^O_2_ is its faster surface exchange with C^18^O_2_ compared to that with ^18^O_2_ (as marked in Section 3.3) [58,145,146,147,148], which generally allows one to avoid the limitation of the process by the surface exchange (i.e., to carry out the experiments in the diffusion-controlled or mixed-controlled regime) [153] and to obtain more detailed data on the oxygen bulk diffusion [58,69,161]. The comparison of various types of isotope exchange experiments with gas phase analysis is given in Table 1.

The reaction of isotope exchange of the solid states with the gas-phase reagent containing two identical atoms (^18^O_2_, C^18^O_2_, D_2_, D_2_O, etc.) proceeds via routes which are classified as three types of exchange mechanisms according to Muzykantov’s classification (Equations (46)–(48)) [165]:

- Homoexchange:
  o R^0^-type (0-atomic type, I type):

X_2_ + Y_2_ = 2 XY; (46)

- Heteroexchange:
  o R^1^-type (1-atomic type, II type):
X_2_ + (Y)_S_ = XY + (X)_S_, (47)
  o R^2^-type (2-atomic type, III type):

X_2_ + 2 (Y)_S_ = Y_2_ + 2 (X)_S_. (48)

In many cases, several types of exchange occur simultaneously.

If isotope exchange takes place with molecules containing more than two identical atoms, the mechanism can be more complex. For example, for hydrogen isotope exchange with methane, the theory of five types of exchange mechanisms is used [166,167].

**Figure 11:**
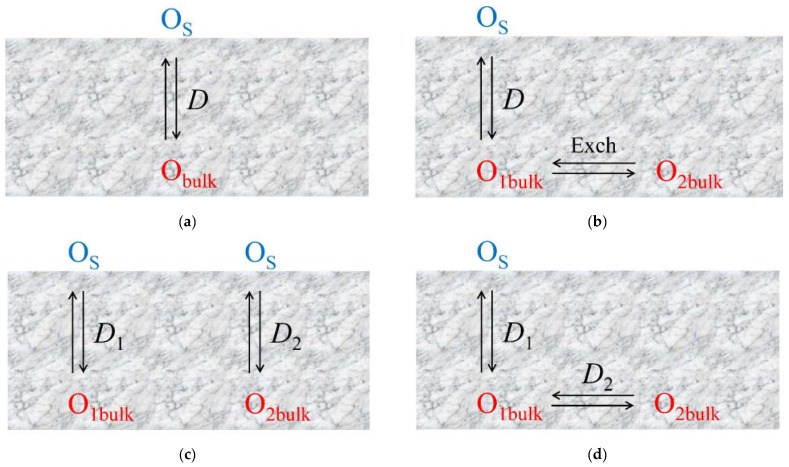

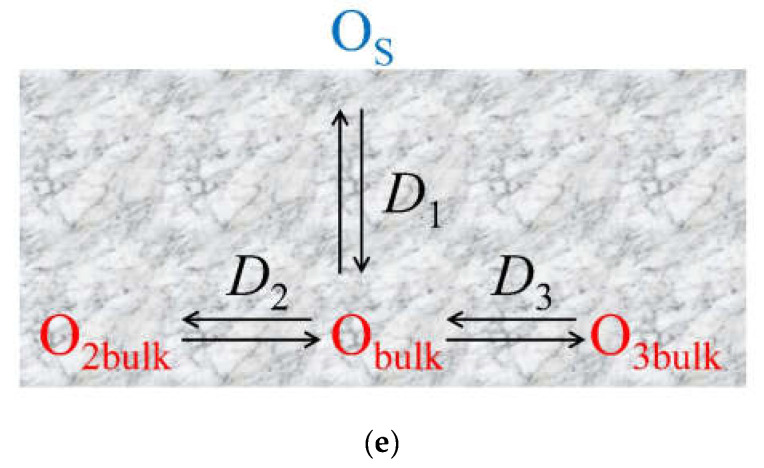
Models for the description of oxygen diffusion in oxides and composites: (**a**) Uniform 1D model; (**b**) non-uniform 1D model with a single diffusion channel and an exchange with neighboring oxygen forms; (**c**) non-uniform 1D model with several parallel diffusion channels; (**d**) non-uniform 2D model with a single diffusion channel along grain boundaries with subsequent diffusion within the grain bulk; and (**e**) non-uniform 2D model with a single diffusion channel along grain boundaries with subsequent diffusion within the bulk of different grains.

Several models or combinations thereof are used to describe oxygen diffusion in the bulk [58,162,163,164]:

- Uniform 1D model (e.g., simple oxides) (Figure 11a);
- Non-uniform 1D model with a single diffusion channel involving the weakest bound oxygen form and an exchange with the neighboring strongly bound oxygen forms (complex oxides) (Figure 11b);
- Non-uniform 1D model with several parallel diffusion channels involving different oxygen forms (composites) (Figure 11c);
- Non-uniform 2D model with a single fast diffusion channel along grain boundaries followed by diffusion of the isotope tracer within the grain bulk (monocrystalline) (Figure 11d);
- Non-uniform 2D model with a single fast diffusion channel along grain boundaries with subsequent diffusion of the isotope tracer within the balk of different grains (polycrystalline) (Figure 11e).

The generalized model of oxygen isotope exchange is the following (Equations (49)–(54)):

(49) $$N_{g}\frac{\partial \alpha_{g}}{\partial t}+O=N_{S}R^{\Sigma }(\alpha_{s}-\alpha_{g})+O(N_{g},\alpha_{g}),$$

(50) $$\frac{\partial \alpha_{s}}{\partial t}=R^{\Sigma }(\alpha_{g}-\alpha_{s})-\frac{N_{bulk}}{N_{s}}{\left.\frac{D}{h^{2}}\frac{\partial \alpha_{bulk}}{\partial \eta }\right|}_{\eta =0},$$

(51) $$\frac{\partial \alpha_{bulk}}{\partial t}=\frac{D}{h^{2}}\frac{\partial^{2}\alpha_{bulk}}{\partial \eta^{2}},$$

(52) $$N_{g}\frac{\partial f_{16-18}}{\partial t}=N_{s}R^{\left(i\right)}(P_{16-18}-f_{16-18})+O(N_{g},f_{16-18}),$$

where *N_g_*, *N_S_* and *N_bulk_* are numbers of oxygen atoms in the gas phase, on the sample surface and in the sample bulk, respectively; *α_g_*, *α_S_* and *α_bulk_* are ^18^O atomic fractions in the gas phase, on the sample surface and in the sample bulk, respectively; *O*(*N_g_*,*α_g_*) is a component which depends on the reactor type,

(53) $$O(N_{g},\alpha_{g})=\left\{\begin{array}{l} 0\;\;\;\;\;\;\text{-}\mathrm{static}\;\mathrm{reactor}\\ \frac{1}{\tau }N_{g}\frac{\partial \alpha_{g}}{\partial \xi }\;\;\text{-}\mathrm{flow}\;\mathrm{reactor} \end{array}\right.\;;$$

*R* and *D* are the heteroexchange rate and oxygen tracer diffusion coefficient, respectively, which are assumed to be constant for isothermal isotope exchange (IIE) experiments and dependent on the temperature according to the Arrhenius law for temperature-programmed (TPIE) experiments:

(54) $$R^{\left(i\right)}=\left\{\begin{array}{l} R^{2}=0.5R^{\Sigma }\\ R^{1}=R^{\Sigma } \end{array}\right.\;P_{16-18}=\left\{\begin{array}{l} 2\alpha_{s}(1-\alpha_{s}){\;\;\;\;\;\;\;\text{-}\mathrm{exchange}\;\mathrm{with}\;O}_{2\;}\;\\ \alpha_{g}(1-\alpha_{s})+\alpha_{s}(1-\alpha_{g}){\;\text{-}\;\mathrm{exchange}\;\mathrm{with}\;\mathrm{CO}}_{2} \end{array}\right..$$

The mathematical models for specific cases of oxygen diffusivity features (e.g., 2D diffusion) can be found in the works [162,163,164].

## 5. Relaxation Techniques

Relaxation techniques such as the electrical conductivity relaxation (ECR) [168,169,170,171,172,173,174,175,176], mass relaxation (MR, also referred to as weight relaxation or thermogravimetric relaxation) [69,111,112,113,176,177,178] and unit cell volume relaxation (UCVR) [69,162,179] techniques are based on the changing some characteristics of a sample with time after rapid change of the gas-phase composition such as the partial pressure of oxygen, carbon dioxide, hydrogen or water vapors. After such a rapid change, the system solid-state sample–gas phase becomes non-steady-state, and oxygen or/and hydrogen desorbs from the sample or adsorbs on the sample to reach a new steady state. This leads to the relaxation of characteristics dependent on the oxygen and hydrogen content in the sample such as the electrical conductivity, the sample weight, and the unit cell volume to the new steady-state values. These methods allow one to acquire the data on the coupled transport of the mobile particles (oxide anions, protons, holes, etc.) in the materials for SOFC, permselective membranes and other devices (*D_chem_* and *k_chem_*).

The data acquired from the relaxation (ECR, MR or UCVR) experiment are normalized, like those shown in Equation (55) for ECR:

(55) $$\bar{\sigma }\left(t\right)=\frac{\sigma \left(t\right)-\sigma_{0}}{\sigma_{\infty }-\sigma_{0}},$$

where *σ*_0_, *σ*(*t*) и *σ*_∞_ are the sample electrical conductivity before changing pressure, at the moment of time *t* and after the relaxation, respectively; $\bar{\sigma }\left(t\right)$ is the normalized electrical conductivity depending on time, $0\leq \bar{\sigma }\left(t\right)\leq 1$. The experimental data (Equation (55)) are fitted by theoretical curves, which can be found by solving Fick’s second law (Equation (15)). Such a solution was found for the following cases [173,174,180,181,182]:

- The infinite plane sheet (Equations (56)–(62)):

(56) $$\bar{\sigma }\left(t\right)=\sum_{i=1}^{\infty }A_{i}\exp\left(-\frac{t}{t_{i}}\right)+1,$$

where

(57) $$A_{i}=-\frac{2\Lambda^{2}}{\beta_{i}^{2}\left(\beta_{i}^{2}+\Lambda^{2}+\Lambda \right)},$$

(58) $$t_{i}=\frac{l^{2}}{4\beta_{i}^{2}D_{chem}},$$

(59) $$\Lambda =\frac{lk_{chem}}{D_{chem}}=\frac{l}{L_{C,chem}};$$

*β_i_* are the eigenvalues of Equation (60):

(60) $$\beta_{i}\tan\beta_{i}=\Lambda ;$$

*l* is the sheet thickness;

- The infinite cylinder (Equations (61)–(64)):

(61) $$\bar{\sigma }\left(t\right)=\sum_{i=1}^{\infty }A_{i}\exp\left(-\frac{t}{t_{i}}\right)+1,$$

where

(62) $$A_{i}=-\frac{2\Lambda_{\rho }^{2}}{\rho_{i}^{2}\left(\rho_{i}^{2}+\Lambda_{\rho }^{2}\right)},$$

(63) $$t_{i}=\frac{r^{2}}{\rho_{i}^{2}D_{chem}},$$

and the parameters Λ*_ρ_* and the eigenvalues *ρ_i_* can be found while solving Equation (64):

(64) $$\rho_{j}J_{1}\left(\rho_{j}\right)=\Lambda_{\rho }J_{0}\left(\rho_{j}\right)=\frac{rk_{chem}}{D_{chem}}J_{0}\left(\rho_{j}\right),$$

where *J*_0_ and *J*_1_ are the zero-order and the first-order Bessel functions, respectively; *r* is the cylinder radius;

- The short cylinder (Equations (65)–(67)):

(65) $$\bar{\sigma }\left(t\right)=\sum_{i=1}^{\infty }\sum_{j=1}^{\infty }A_{ij}\exp\left(-\frac{t}{t_{ij}}\right)+1,$$

where

(66) $$A_{ij}=-\frac{2\Lambda^{2}}{\beta_{i}^{2}\left(\beta_{i}^{2}+\Lambda^{2}+\Lambda \right)}\frac{2\Lambda_{\rho }^{2}}{\rho_{i}^{2}\left(\rho_{i}^{2}+\Lambda_{\rho }^{2}\right)},$$

(67) $$t_{ij}=\frac{1}{D_{chem}\left[{\left(\frac{\beta_{i}}{l/2}\right)}^{2}+{\left(\frac{\rho_{j}}{r}\right)}^{2}\right]};$$

- The sphere (Equations (68)–(71)):

(68) $$\bar{\sigma }\left(t\right)=\sum_{i=1}^{\infty }A_{i}\exp\left(-\frac{t}{t_{i}}\right)+1,$$

where

(69) $$A_{i}=-\frac{6\Lambda_{\rho }^{2}}{\rho_{i}^{2}\left(\rho_{i}^{2}+\Lambda_{\rho }\left(\Lambda_{\rho }-1\right)\right)},$$

(70) $$t_{i}=\frac{r^{2}}{\rho_{i}^{2}D_{chem}},$$

and the eigenvalues *ρ_i_* can be found while solving Equation (71):

(71) $$\rho_{i}\cot\rho_{i}-\Lambda_{\rho }-1=0;$$

*r* is the sphere radius;

- The rectangular bar (Equations (72)–(75)):

(72) $$\bar{\sigma }\left(t\right)=\sum_{i=1}^{\infty }A_{x,i}\exp\left(-\frac{t}{t_{x,i}}\right)\times \sum_{j=1}^{\infty }A_{y,j}\exp\left(-\frac{t}{t_{y,j}}\right)+1,$$

where

(73) $$A_{x,i}=-\frac{2\Lambda_{x}^{2}}{\beta_{x,i}^{2}\left(\beta_{x,i}^{2}+\Lambda_{x}^{2}+\Lambda_{x}\right)},\ldots A_{y,j}=-\frac{2\Lambda_{y}^{2}}{\beta_{y,i}^{2}\left(\beta_{y,i}^{2}+\Lambda_{y}^{2}+\Lambda_{y}\right)},$$

(74) $$t_{x,i}=\frac{l_{x}^{2}}{4\beta_{i}^{2}D_{chem}},\ldots t_{x,i}=\frac{l_{x}^{2}}{4\beta_{i}^{2}D_{chem}},$$

(75) $$\Lambda_{x}=\frac{l_{x}k_{chem}}{D_{chem}},\ldots \Lambda_{y}=\frac{l_{y}k_{chem}}{D_{chem}},$$

*l_x_* and *l_y_* are the bar dimensions along the *x* and *y* axes, respectively.

For a more complex sample shape or, all the more so, for any geometrical shape, a more complex approach is required such as mathematical modeling, e.g., the generalized model based on the inverse algorithm [172,175].

The reactor flush time (*t_f_*) is taken into account by introducing the factor $\frac{t_{i}}{t_{i}+t_{f}}$ and the addendum $\exp\left(-\frac{t}{t_{f}}\right)$ into Equations (56), (61), (65), (68) and (72) [182]. E.g., for the short cylinder case, the best description of disc-shaped pelletized samples, Equation (65) transforms into Equation (76):

(76) $$\bar{\sigma }\left(t\right)=\sum_{i=1}^{\infty }\sum_{j=1}^{\infty }A_{ij}\frac{t_{ij}}{t_{ij}-t_{f}}\left[\exp\left(-\frac{t}{t_{ij}}\right)-\exp\left(-\frac{t}{t_{f}}\right)\right]-\exp\left(-\frac{t}{t_{f}}\right)+1.$$

The relaxation techniques are being developed up to now, and include new approaches for processing the experimental data, elucidating the contribution of each phase and interphase of composite materials, the analysis of different charge carrier behavior in triple-conductive materials, etc. [111,112,113,168,170,172,175,178].

## 6. Oxygen and Hydrogen Mobility of Materials for Membranes and SOFC

### 6.1. Fluorites, Bixbyites and Rhombohedral Phases

Fluorites and fluorite-related materials are the most of important types of materials for application in electrochemistry including SOFCs/SOECs, oxygen/hydrogen separation membranes, etc.

Recent research efforts [183,184,185,186,187] have made it possible to increase the grain-boundary conductivity of proton-conducting zirconates with a perovskite structure. At the same time, there is another class of proton-conducting materials, with a fluorite-like structure, which have comparable total and bulk conductivities, whereas the contribution of grain-boundary conductivity is extremely small or zero. This class of materials comprises the following disordered pyrochlores and fluorites based on La compounds:

1. Ca-doped La_2_Zr_2_O_7_ ((La_2−x_Ca*_x_*)Zr_2_O_7−δ_) pyrochlore, a proton conductor in the range of 200–600 °C [188,189];
2. La_2_Ce_2_O_7_ (50% CeO_2_ + 50% La_2_O_3_) fluorite, a proton conductor below 450 °C and an oxygen ion conductor at high temperatures [190]; and
3. Fluorite-like La_6−x_WO_12−δ_ (x = 0–0.8), a proton conductor with conductivity up to (3–7) × 10^−3^ S cm^−1^ at 800 °C and 1 Pa, depending on *x* [82,191].

*Ln* tungstates were revealed to have mixed ionic–electronic conductivity with a potential ability of using in solid oxide fuel cells and proton conducting membranes [82,191]. La_6−x_WO_12−δ_ (x = 0.2–1) solid solutions based on lanthanum tungstate La_6_WO_12_ were of particular interest since they were found to have the highest proton conductivity among the few non-perovskite proton-conducting materials [82,191,192,193,194,195]. La_6−x_WO_12−δ_ (x = 0.2–1) tungstates can be used as potential solid electrolytes for solid-state fuel cells and proton-conducting membranes for hydrogen separation. An important advantage of lanthanum tungstates over perovskite-acceptor-doped barium and strontium cerates BaCeO_3_, SrCeO_3_–is the absence of interaction with CO_2_ and SO*_x_* with the formation of carbonates and compounds containing sulfur [192].

Among single-phase materials La_6−x_WO_12−δ_ (x = 0–0.8), the highest proton conductivity was provided by La_6−x_WO_12−δ_ (x = 0.4, 0.5) materials, but subsequent investigation showed that their proton conductivity dropped rather sharply during prolonged holding in wet H_2_ at 1100 °C, and the most stable materials were La_6−x_WO_12−δ_ with x = 0.6 and 0.7 [82]. According to Partin et al. [196], who prepared samples by standard solid-state reactions, the most stable solid solution was La_6−x_WO_12−δ_ with x = 0.4. It seems likely that the problem of low grain-boundary conductivity arises as well in the case of proton-conducting lanthanum tungstates. For example, in studies of the conductivity of La_6−x_WO_12−δ_ (x = 0.4, 0.6, 0.8, 1.0) [196], comparison of impedance plots before and after holding in a wet atmosphere showed a marked increase in grain-boundary resistance at 800–900 °C. By contrast, in the range 300–500 °C, the grain-boundary resistance decreased with increasing partial pressure in various atmospheres [82,196]. Since W^6+^ and Mo^6+^ are similar in ionic radius, Savvin et al. [197,198] expected to obtain proton-conducting materials based on the *Ln*_6_MoO_12_ (*Ln* = La–Lu) molybdates. Indeed, they succeed to extend the class of proton-conducting fluorite-like materials by synthesizing new mixed electron–proton-conducting molybdates: La_5.8_Zr_0.2_MoO_12.1_ and *Ln*_5.4_Zr_0.6_MoO_12.3_ (*Ln* = Nd, Sm, Dy) [197,198]. Doping with zirconium ensured a higher stability of molybdates to reduction, but as in the case of tungstates [191], Zr was found to be a donor dopant, reducing the proton conductivity of materials [197]. Among proton-conducting *Ln*_6−x_Zr*_x_*MoO_12+δ_ (*Ln* = La, Nd, Sm, Gd, Dy, Ho; x = 0.2–0.6) molybdates, most of which have a fluorite structure (sp. gr. $Fm\bar{3}m$), the highest conductivity was found for the rhombohedral La_5.8_Zr_0.2_MoO_12.1_ phase (sp. gr. $R\bar{3}$), which exhibited a total conductivity of 2.5 × 10^−5^ S cm^−1^ at 500 °C (3 × 10^−4^ S cm^−1^ at 800 °C) in wet air [198]. It should be noted that solid solutions based on rare-earth tungstates and molybdates are predominantly oxygen ion conductors in dry air at low temperatures, and predominantly proton conductors in wet air [82,198]. At high temperatures (above 600 °C) in an oxidizing atmosphere (air), the charge transport is dominated by *p*-type conduction, whereas under reducing conditions *n*-type conduction prevails [82,198]. Doping with Ti, Zr, and Nb on the Mo site and with fluorine on the oxygen site was studied using La_5.4_MoO_11.1_ as an example, but essentially all of the dopants reduced ionic conductivity of the material [197,199,200]. A similar situation was observed in La_6−x_WO_12−δ_ (x = 0.4, 0.5) lanthanum tungstates [82,191,192,193,194,195]. Due to the fact that cation doping [197,199,200,201] decreased the proton conductivity of RE molybdates, the main attention was paid to the study of pure solid solutions based on *Ln*_6_MoO_12_: *Ln*_6−*x*_MoO_12−δ_ (*Ln* = La, Nd, Sm, Gd–Lu) [163,202,203,204,205,206,207,208,209,210,211,212]. It is known that, to a large extent, the proton conductivity depends on the crystal structure type, and, in this regard, the rich polymorphism of solid solutions based on RE molybdates and tungstates *Ln*_6_MO_12_ (M = Mo, W) should be noted [199,201,203,205,206,209,210,212]. In the series *Ln*_6−*x*_MoO_12−δ_ (*Ln* = La, Nd, Sm, Gd–Lu), depending on the temperature and lanthanide ionic radii, various structural types are realized: rhombohedral $\left(R\bar{3}\right)$, fluorite $\left(Fm\bar{3}m\right)$, and bixbyite $\left(Ia\bar{3}\right)$. Proton conductivity was found in various solid solutions based on RE molybdates, and it was shown that it reached maximal values for lanthanum molybdates La_6−*x*_MoO_12−δ_ (x = 0.5, 0.6) with a complex rhombohedral structure *R*1 [202,203,211].

The stability of solid solutions based on REE molybdates, as well as of lanthanum tungstates La_6−x_WO_12−δ_ (x = 0–0.8) solid solutions, known proton conductors [82,191,192,193,194,195], is an important issue in the perspective of their practical application. As a rule, it is the process of reduction in variable valence cations in solid solutions, which results in a grain-boundary contribution growth, limiting the conductivity of materials in wet atmospheres at high temperatures. The stability of the Ho_5.4_Zr_0.6_MoO_12.3_ fluorite structure and the La_6−*x*_MoO_12−*δ*_ (x = 0.5) fluorite-like rhombohedral structure R1 in extremely dry conditions under dynamic vacuum was investigated by in situ variable temperature neutron diffraction (NDD) between 800 and 1400 °C [205]. The NDD results unambiguously demonstrated the dimensional stability of the fluorite-like rhombohedral La_6−*x*_MoO_12−*δ*_ (x = 0.5) as compared to the Ho_5.4_Zr_0.6_MoO_12.3_ fluorite in the heating–cooling cycle. According to the NDD, heating to 1100 °C followed by vacuum cooling does not change the *c* cell parameter of rhombohedral La_6−*x*_MoO_12−δ_ (x = 0.5), whereas its *a* parameter decreases by 0.13%. It was also found that the *a* cell parameter of cubic fluorite Ho_5.4_Zr_0.6_MoO_12.3_ decreases by ~2.6%. It may be result of the partial reduction of Mo^6+^ to Mo^+5^ in RE molybdates. It seems likely that the same cause, i.e., the decrease in cubic cell parameter as a result of the partial reduction of W^6+^ to W^+5^, accompanied by disordering on the La/W sites, and subsequent formation of a denser atomic packing in the La_6-*x*_WO_12−δ_ (x = 0.4, 0.6, 0.8) lanthanum tungstates, underlies their relatively low stability [196,198,213,214,215]. We believe that the loss of dimensional stability under reducing conditions in *Ln*_6_MO_12_ (M = Mo, W)-based solid solutions, which results in a grain-boundary contribution, limiting their conductivity in wet atmospheres, is due to the partial reduction of Mo^6+^ and W^6+^ in the rare-earth molybdates and tungstates, respectively [205].

**Figure 12:**
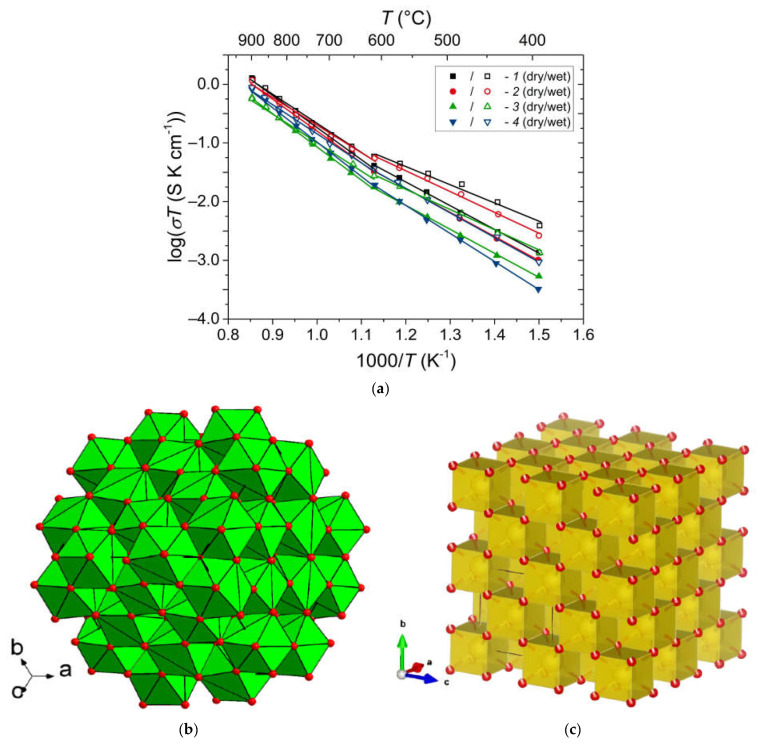
(**a**) Temperature dependence of total conductivity for La_6−*x*_MoO_12−δ_ in dry and wet air: (1) x = 0.5; (2) x = 0.6; (3) x = 0.7; (4) x = 1; (**b**) bixbyite $\left(Ia\bar{3}\right)$; (**c**) fluorite $\left(Fm\bar{3}m\right)$.

A follow-up study of the structure of La-containing molybdates La_6−*x*_MoO_12−δ_ (x = 0.5, 0.6) showed that they have a new structure type based on rhombohedral cells, which has been discussed in series of papers [199,203,205,206,209,210,212]. Along with main peaks of the $R\bar{3}$ [205] or $R\bar{3}m$ [209] structure, additional lines are present. These are superstructure lines typical of complex crystallographic cells whose parameters are increased by seven (R1) or five (R2)) times according to López-Vergara et al. [209]. López-Vergara et al. [203] reported that, depending on the cooling rate, the La_6−*x*_MoO_12−δ_ (x = 0.6) solid solution can be obtained either in the form of a complex rhombohedral modification R1 (slow cooling) or in the form of fluorite (quenching), which agrees with the high-temperature experiment in vacuum for La_6−*x*_MoO_12−δ_ (x = 0.5) [205]. It also turned out that R1 phase La_6−*x*_MoO_12−δ_ (x = 0.6) has better oxygen-ion and proton conductivity than that of fluorite [203,209]. The decrease in the lanthanum concentration led to a decrease in the rhombohedral distortion degree and to the decrease in the contribution of proton conductivity in the series La_6−*x*_MoO_12−δ_ (x = 0.5, 0.6, 0.7, 1) [211]. The proton conductivity for the optimal composition of La_6−*x*_MoO_12−δ_ (x = 0.5) was ~5 × 10^−5^ S cm^−1^ at 500 °C in wet air, while for La_6−*x*_MoO_12−δ_ (x = 1) it was ~9 × 10^−6^ S cm^−1^ (Figure 12a) [30].

A tendency towards a decrease in the proton conductivity contribution for the rare-earth (RE) molybdates *Ln*_6−*x*_MoO_12−δ_ (*Ln* = La–Yb) series has been established. For heavy RE molybdates, the conditions for the synthesis of new proton conductors with a bixbyite structure (Figure 12b) were found for the first time [202,204,206,208,212], and the bixbyite structure type was first presented in the ICDD PDF crystallographic database (Er_6_MoO_12−δ_ (No. I11624) and Tm_6_MoO_12−δ_ (No. I11626)). It was found that with decreasing of the *Ln*_2_O_3_ content by 1.8 mol.%, fluorites *Ln*_5.5_MoO_11.25−δ_ (*Ln* = Er, Tm) are formed under the same conditions (Figure 12c) [212].

**Figure 13:**
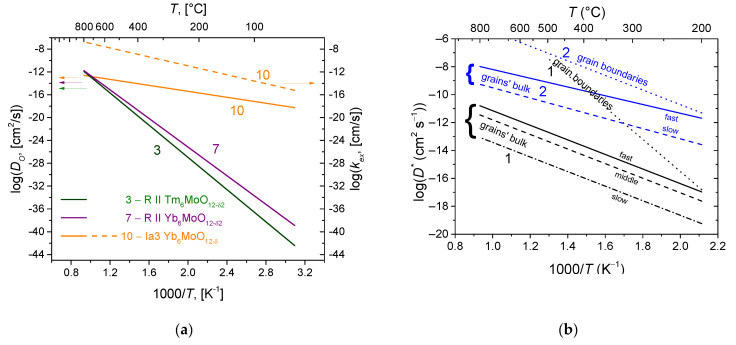
(**a**) Arrhenius plots of oxygen tracer diffusion coefficients and surface exchange constants for rhombohedral Tm_6_MoO_12−δ_, rhombohedral Yb_6_MoO_12−δ_, and bixbyite Yb_6_MoO_12−δ_. Reprinted with permission from Ref. [206]. Copyright 2019 American Chemical Society. (**b**) Arrhenius plots of oxygen tracer diffusion coefficients for Nd_5.5_WO_11.25−δ_ (1) [216] and Nd_10_Mo_2_O_21_ (2) [163].

Fluorites and bixbyites turned out to be mixed electron-oxygen conductors in dry air and electron-proton conductors in wet air, while the dominant ionic contribution maintains up to 550–600 °C [163,202,206]. In wet air, Er and Tm fluorites and bixbyites had a close total conductivity of ~2 × 10^−6^ S cm^−1^ at 500 °C, but at 200 °C, bixbyites performed better than that of fluorites. The using of the isotope exchange with C^18^O_2_ made it possible to confirm the high mobility of oxygen in these compounds in air, starting from 200 °C (Figure 13) [212]. A high or at least intermediate oxygen mobility was demonstrated for other fluorites and bixbyites (in some cases due to defect features such as the effect of grain boundaries resulting in a fast oxygen diffusion along grain boundaries (2D diffusion)), while rhombohedral phases possess lower oxygen mobility (Figure 13) [55,58,163,206,212,216].

It is of interest to note that the existence of compounds and solid solutions with close composition, differing by only a few mole percent, but having different structure, is typical for the L*n*_2_O_3_–Mo(W)O_3_ (L*n* = La, Nd, Pr, Sm) systems [217,218,219]. For example, in the Pr_2_O_3_–MoO_3_ and Nd_2_O_3_-MoO_3_ systems at 1000 °C, the compounds with *Ln*_2_O_3_:MoO_3_ (*Ln* = Pr, Nd) molar ratios of 5:6 and 7:8 differ in composition by just ~3 mol.% [217]. According to Chambrier et al. [218,219], cubic solid solutions based on La_10_W_2_O_21_ free of La_2_O_3_ and La_6_W_2_O_15_ impurities exist up to ~1700 °C in a narrow composition range, 26–30 mol.% WO_3_, and La_10_W_2_O_21_ exact composition is 28.6 mol.% WO_3_ + 71.4 mol.% La_2_O_3_. La_6_WO_12_ contains 25 mol.% WO_3_. Thus, in the Ln_2_O_3_–WO_3_ system, La_6_WO_12_ and La_10_W_2_O_21_ differ in composition by just 3.6 mol.% WO_3_.

**Figure 14:**
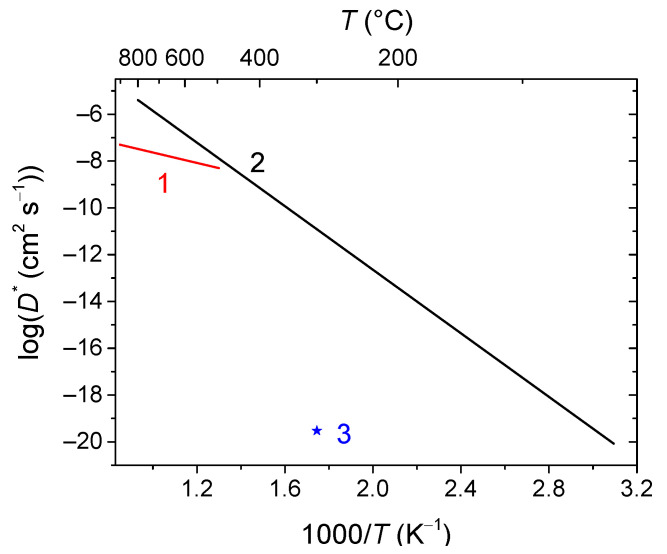
Arrhenius plots of oxygen tracer diffusion coefficients for Ce_0.9_Pr_0.1_O_2−δ_ (1) [221], Ce_0.65_Pr_0.25_Y_0.1_O_2−δ_ (2) [69] and Ce_0.8_Tb_0.2_O_2−δ_ (3) [224].

Doped ceria materials being typically pure ionic conductors in air and MIECs in reducing atmospheres are generally used as intermediate-temperature SOFC buffer layers between the electrolyte and the cathode in order to prevent their chemical interaction as well as electrolytes or components of composites for intermediate-temperature SOFC electrodes and oxygen separation membranes [33,36,39,53,54,58,69,70,107,220]. For using ceria as electrode or membrane material itself, the electronic component of conductivity should be increased. This can be achieved by doping with cations possessing redox activity such as Pr^4+/3+^ and Tb^4+/3+^ [70,220]. Doping with Pr leads to an increase in oxygen mobility and surface reactivity as well, due to the formation of ordered chains of Pr^4+/3+^ cations [69,221,222]. For Tb-doped ceria, it was demonstrated that it possesses a high oxygen heteroexchange rate comparable with that for Gd-doped ceria [220,222]. On the other hand, it was demonstrated that the oxygen mobility of Ce_1−x_Tb_x_O_2−δ_ (x = 0, 0.2 and 0.5) decreases with increasing Tb content, probably due to interaction between defects resulting in forming local associates [223,224]. Nevertheless, the oxygen permeability of membranes based on some Pr- and Tb-doped ceria was comparable to that for similar membranes based on perovskites such as LFN and LSFC [70,220]. Figure 14 demonstrates comparison of the oxygen tracer diffusion coefficient values of MIEC-doped ceria materials.

### 6.2. Pyrochlores

**Figure 15:**
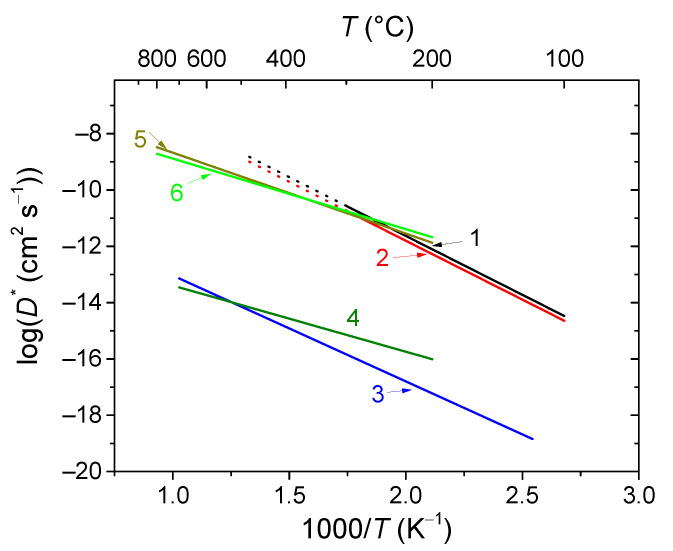
Arrhenius plots for oxygen tracer diffusion coefficients for Bi_2_Ce_2_O_7_ (1) [228], Bi_1.6_Y_0.4_Ce_2_O_7_ (2) [228], Bi_1.6_Y_0.4_Ti_2_O_7_ (3) [228], Bi_1.6_Sc_0.2_Ti_2_O_7−δ_ (4) [232], Bi_1.6_Mg_0.2_Ti_2_O_7-δ_ (5) [232] and Bi_1.6_Zn_0.2_Ti_2_O_7−δ_ (6) [231]. Adapted from Ref. [228] under the CC BY 4.0 license.

The pyrochlore structure A_2_B_2_O_7_ is a derivative of the fluorite structure in which half of the cubes are replaced by octahedra (more precisely, it consists of the alternating AO_8_ polyhedra and BO_6_ trigonal antiprisms). Pyrochlores possessing a highly mixed ionic-electronic conductivity such as doped Pr_2_Zr_2_O_7_, Gd_2_Ti_2_O_7_, Er_2_RuMnO_7_, etc., are used in SOFC cathodes [164,225,226], oxygen [83,227,228] and hydrogen separation membranes [229,230]. They contain high amounts of oxygen vacancies providing fine oxygen transport characteristics. Some pyrochlores contain interstitial oxide anions formed due to Frenkel disordering (Equation (77))

(77) $$O_{O}^{\times }=V_{O\;(48f)}^{••}+{\;O^{\prime \prime }}_{i\;(8a)}$$

involved in the oxygen diffusion as well [137]. There are two forms of oxygen in the pyrochlore structure (O, O′), of which the content ratio is 6:1. However, according to TPIE C^18^O_2_ studies [56,58,164,228,231,232,233], the oxygen bulk mobility is uniform, or, in the case of its nonuniformity, the ratio of various oxygen forms differing in their mobility differs from 6:1. This is evidence that the oxygen migration mechanism is rather complex and includes the oxygen of both O- and O’-sublattices. It was proposed as well that the oxygen forms differing in their mobility can be associated with A–O–A, A–O–B and B–O–B migration pathways with their fraction depending on the partial disordering of the pyrochlore structure [164,228]. The other feature of some pyrochlores (Mg-doped Sm and Gd zirconates) is the fast oxygen transport along grain boundaries being characterized by a very high mobility (D* ~10^−7^ cm^2^ s^−1^ at 1000 K) [164]. The comparison of the oxygen mobility of some pyrochlores is given in Figure 15.

Shimura et al. [234] studied the proton conductivity of Ln_2_Zr_2_O_7_-based (Ln = La, Nd, Sm, Gd и Er) pyrochlore oxides and found that the conductivity of the Ln_2_Zr_1.8_Y_0.2_O_7−δ_ (Ln = La, Nd, Sm, Gd и Er) solid solutions in a hydrogen atmosphere at *T* > 600 °C was comparable to that of perovskites. The effect of alkaline earth cation (Mg, Ca, Sr, and Ba) and Y substitutions for both the La and Zr sites in pyrochlore La_2_Zr_2_O_7_ on its proton conductivity was studied in detail in [188,189,234,235]. The highest proton conductivity was obtained by substituting La with Ca and Sr. The conductivity of (La_1.97_Ca_0.03_)Zr_2_O_7−δ_ between 600 and 700 °C was determined to be 4 × 10^−4^ S cm^−1^ [188]. It is important to note that the degree of Ca substitution in such solid solutions is low, and not higher than x = 0.05 in (La_2−x_Ca_x_)Zr_2_O_7−δ_. Eurenius et al. [236,237] recently studied the proton conductivity of rare-earth stannates and titanates with the pyrochlore structure A_2−x_Ca_x_Sn_2_O_7−x/2_ (A = La, Sm, Yb) and Sm_2_Ti_1.92_Y_0.08_O_7−δ_, Sm_1.92_Ca_0.08_Ti_2_O_7−δ_. The conductivity of the A-site acceptor-substituted pyrochlores was about one order of magnitude higher than that of the B-site substituted materials. On the other hand, the conductivity clearly depended on the nature of the B-site cation: an increase in the ionic radius and electronegativity of the B-site cation was accompanied by the increase in conductivity. The proton conductivity of the samarium titanate-based solid solutions, and especially that of the rare-earth stannates, was found to be lower than that of the Ca-doped La_2_Zr_2_O_7_.

Calcium- and strontium-doped lanthanum zirconates, La_2−x_D_x_Zr_2_O_7−δ_ (x = 0.05, 0.1; D = Ca, Sr), were extensively studied as electrolyte materials for proton-conducting solid oxide fuel cells (PC-SOFCs) [188,235,238,239,240]. Calcium appears to be the most promising dopant because strontium doping results in the formation of a second phase, SrZrO_3_ with a perovskite structure, on the surface of strontium-containing zirconate ceramics [239] and, more importantly, because the overall conductivity of strontium-containing ceramics is an order of magnitude lower than that of calcium-containing ceramics. It was reported that pyrochlore solid solutions La_1.95_Ca_0.05_Zr_2_O_6.95_ and La_1.9_Ca_0.1_Zr_2_O_6.9_ were almost identical in proton conductivity [188,238]: 7.0 × 10^−4^ S cm^−1^ at 600 °C. As was shown earlier [241], the proton conductivity of Sm_2−x_Ca_x_Zr_2_O_7−δ_ (x = 0.05) at 600 °C is ~7.5 × 10^−4^ S cm^−1^ [241].

Gas-tight proton-conducting Nd_2−x_Ca_x_Zr_2_O_7−δ_ (x = 0, 0.05) ceramics were prepared for the first time via mechanical activation of the oxide mixture, followed by the single-step firing at 1600 °C for 3 or 10 h [242]. Like in the case of (Ln_1−x_Ca_x_)_2_Zr_2_O_7−x_ (Ln = La, Sm; x = 0.05) pyrochlore solid solutions, the unit-cell parameter of the Ca-doped material Nd_2−x_Ca_x_Zr_2_O_7−δ_ (x = 0.05) was smaller than that of the undoped Nd_2_Zr_2_O_7_. The Rietveld-refined XRD data demonstrated that Ca substitutes on both cation sites of zirconate and that most of the Ca cations resides in the Zr sublattice. As a result, the total conductivity of Nd_2−x_Ca_x_Zr_2_O_7−δ_ (x = 0.05) in wet air was lower than that of the (Ln_1−x_Ca_x_)_2_Zr_2_O_7−x_ (Ln = La, Sm; x = 0.05) pyrochlores, where Ca substituted predominantly on the Ln site. The proton conductivity in wet air was 3 × 10^−4^ S cm^−1^ at 500 °C (7 × 10^−4^ S cm^−1^ at 600 °C) in (La_1−x_Ca_x_)_2_Zr_2_O_7−x_ (x = 0.05), 7 × 10^−5^ S cm^−1^ at 500 °C (~2 × 10^−4^ S cm^−1^ at 600 °C) in (Nd_1−x_Ca_x_)_2_Zr_2_O_7−x_ (x = 0.05), and 1 × 10^−4^ S cm^−1^ at 500 °C (7.5 × 10^−4^ S cm^−1^ at 600 °C) in (Sm_1−x_Ca_x_)_2_Zr_2_O_7−x_ (x = 0.05). Even though the total conductivity of the Ca-doped zirconate Nd_2−x_Ca_x_Zr_2_O_7−δ_ (x = 0.05) was an order of magnitude higher than that of Nd_2_Zr_2_O_7_, predominant Ca substitution on the Zr site leads to a lower proton conductivity in comparison with that of (Ln_1−x_Ca_x_)_2_Zr_2_O_7−x_ (Ln = La, Sm; x = 0.05), where all of the Ca cations resided on the Ln site. It is also possible that this result was due to the higher firing temperature: the (Ln_1−x_Ca_x_)_2_Zr_2_O_7−x_ (Ln = La, Sm; x = 0.05) materials were prepared by firing at 1550 °C for 10–50 h [188,241], whereas a higher firing temperature of 1600 °C (3 and 10 h) was chosen for (Nd_1−x_Ca_x_)_2_Zr_2_O_7−x_ (x = 0.05) in order to obtain gas-tight ceramics.

### 6.3. Perovskites

**Figure 16:**
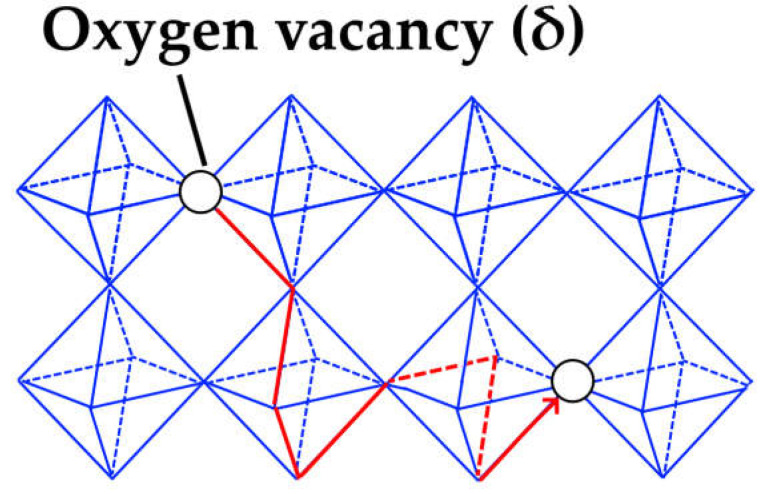
Oxygen vacancy migration path for perovskite-like oxides. Reprinted from Ref. [133] under the CC BY 3.0 license.

**Figure 17:**
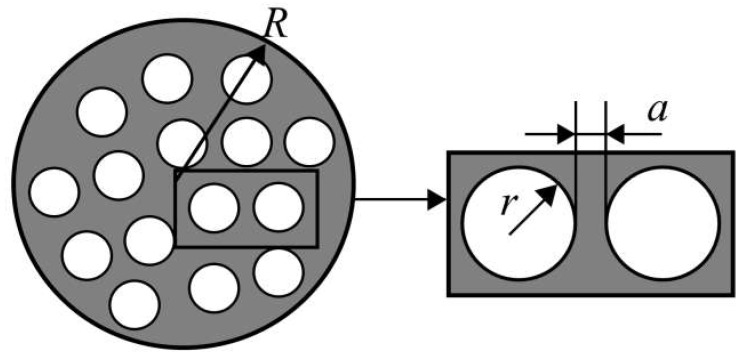
Regions of slow (white) and fast (grey) diffusion. Here, *R*, *r* and *a* are the average particle size, average domain size and grain boundary thickness, respectively [123]. Reprinted from Ref. [123]. Copyright 2006 Elsevier.

Perovskite-like oxides are widely used materials for SOFC and permselective membranes components due to their typically high electronic or mixed ionic-electronic conductivity [55,99,107,128,129,131,152,243,244,245]. The general oxygen transport mechanism in perovskites is a vacancy mechanism (Figure 7a and Figure 16). Hence, increasing the oxygen vacancy content can increase the oxygen mobility, which can be achieved by doping A- and B-sites with various aliovalent cations [131,246]. The creation of an A-site deficiency also allows for an increase in the oxygen vacancy content; however, it may result in a decrease in their mobility due to their binding to defect complexes such as $\left[{\;V^{\prime \prime \prime }}_{\mathrm{La}}-V_{O}^{••}\right]$ [131]. For some oxides with distorted perovskite structure, it was demonstrated that significant deviation from oxygen stoichiometry in such materials is accompanied by nanostructuring; at the same time, grain boundaries become fast channel of oxygen transport, while oxygen transport within the grain bulk is slower (Figure 17) [118,119,120,121,122,123].

**Figure 18:**
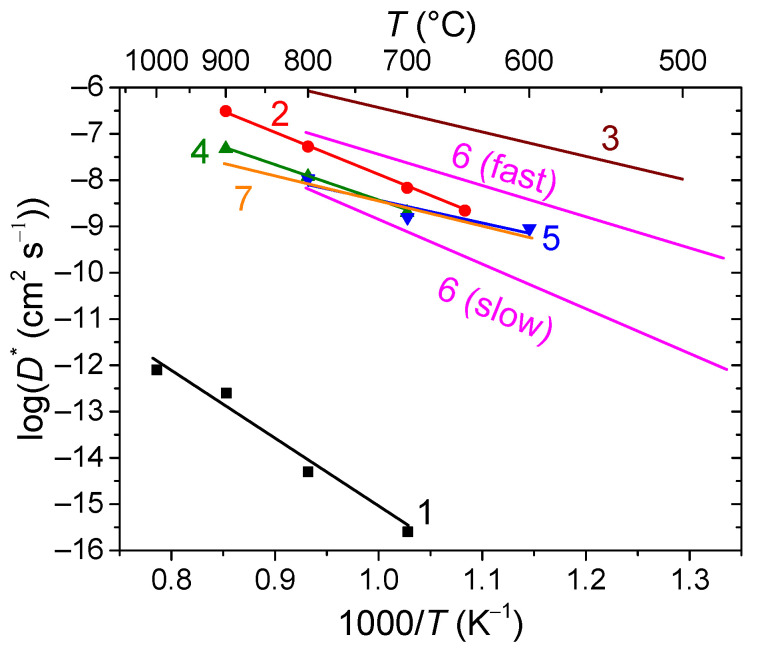
Arrhenius plots for the oxygen tracer diffusion coefficient for various perovskites: 1—La_0.8_Sr_0.2_MnO_3_ [248], 2—La_0.8_Sr_0.2_Fe_0.5_Co_0.5_O_3_ [263], 3—La_0.6_Sr_0.4_CoO_3_ [266], 4—La_0.8_Sr_0.2_Fe_0.7_Ni_0.3_O_3_ [263], 5—LaNi_0.6_Fe_0.4_O_3_ [252], 6—PrNi_0.5_Co_0.5_O_3_ [269], 7—La_0.91_Sr_0.09_ScO_3_ [245].

Conventional strontium-doped lanthanum manganite (LSM) materials have poor oxygen mobility (Figure 18), which limits their application as air electrodes in SOFCs with decreased operating temperatures that are being intensively developed [69,247,248]. However, they can be successfully used in the composite electrodes in combination with different ionic conductors [249,250,251]. Lanthanum ferrite-nickelates (LNF), being predominantly electronic conductors, demonstrate low oxygen diffusion and, as a result, oxygen permeation properties [252,253,254,255]. Nevertheless, LaNi_0.6_Fe_0.4_O_3_, as the most stable in the series, found widespread application in SOCs due to its superior conductivity, low thermal expansion coefficient value, and tolerance to chromium poisoning [256]. It is also successfully used in different composite electrodes for intermediate-temperature SOFCs [257,258,259,260], and as cathode contact materials [261,262]. Materials with mixed oxygen ion and electron conductivity (MIECs), such as Sr-doped lanthanum ferrites-nickelates/cobaltites (LSFN, LSFC) possess much higher oxygen mobility (Figure 18) enabling the O reduction reaction (ORR) along both triple- and double-phase boundaries, thus improving cathode performance, as well as oxygen permeation fluxes across oxygen-separation membranes [69,128,129,247,248,263]. The other state-of-the-art MIEC materials based on Sr-doped La cobaltites (LSC), showing a high catalytic activity in the ORR reaction and a high performance as SOFCs/SOECs air electrodes, demonstrate a high oxygen mobility and surface reactivity as well; moreover, it was reported that LSCs have higher D^*^ values compared to those for LSFCs (D^*^ up to 2 × 10^−7^ cm^2^ s^−1^ at 700 °C, Figure 18) [264,265,266,267,268]. Pr-nickelate-cobaltites (PNC) are stable to carbonation and interaction with electrolytes, which is a well-known issue for Sr-doped perovskites with an La-occupying A-site, possess total conductivity and oxygen diffusivity properties comparable or even exceeding those for LSFN and LSFC [55,58,69,243,269].

**Figure 19:**
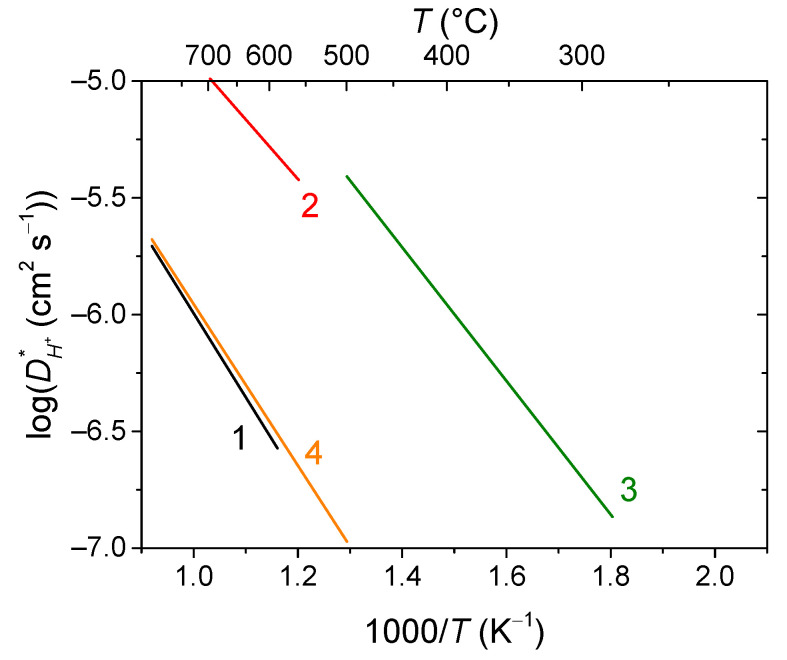
Arrhenius plots for the hydrogen tracer diffusion coefficient for various perovskites: 1—SrCe_0.95_Yb_0.05_O_3_ [272,274], 2—(Ba_0.965_Gd_0.035_)(Ce_0.935_Gd_0.035_)O_3_ [272,275], 3—BaCe_0.9_Y_0.1_O_3_ [272,276], 4—La_0.91_Sr_0.09_ScO_3_ [245].

Mixed protonic-electronic or triple (H^+^/O^2−^/e^−^)-conductive perovskites and their composites based on compositions, such as doped Sr/Ba cerates/zirconates, are the materials for proton-conducting SOFCs (H-SOFC), including high-performance electrodes with triple-conducting behavior [55,111,113,167,183,184,185,186,270,271,272,273], as well as hydrogen separation membranes [55,99,107,139]. Protons in such perovskites are formed due to the hydrogenation or hydration of oxygen vacancies (Equations (40)–(44)). Therefore, one of the factors providing fine protonic transport properties is a high content of oxygen vacancies. Typical values of the hydrogen tracer diffusion coefficient for doped Ba and Sr cerates are ~10^−6^–10^−5^ cm^2^ s^−1^ at 700 °C (Figure 19) [272,273,274,275,276].

The group of promising materials recently studied in applications as low- and intermediate-temperature SOFC cathodes (including H-SOFC), as well as oxygen separation membranes, are double perovskites A_2_B_2_O_6−δ_ or A_2_B_2_O_5+δ_ (A = La, Pr, Ca, Ba, etc., B = Mg, Mo, Sn, Fe, etc.) [55,60,69,243,244,272,277,278,279]. Double perovskites are attractive because they can accommodate a large amount of nonstoichiometric oxygen, as well as having a wide variation in the effective charge of the B-site cations, and having high redox stability and moderate values of the thermal expansion coefficient. Along with this, they possess very high oxygen (D^*^~10^−8^–10^−7^ cm^2^ s^−1^ at 700 °C) and, in some cases, hydrogen mobility (D^*^~10^−6^ cm^2^ s^−1^ at 500 °C) [63,272,280]. The other promising perovskite-based layered materials to be mentioned here are triple [281,282], quadruple [283] and even quintuple perovskites [284].

### 6.4. Ruddlesden–Popper Phases

**Figure 20:**
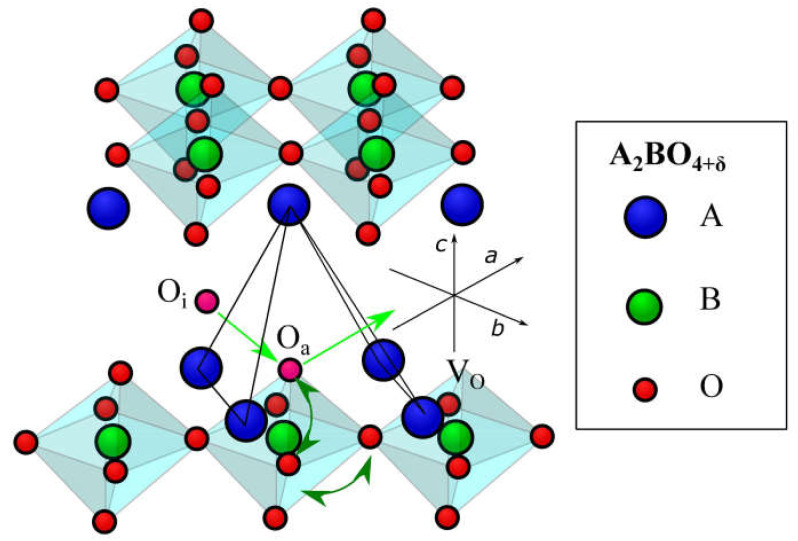
Cooperative mechanism of oxygen migration for Ruddlesden–Popper phases [69]. Reprinted from Ref. [69]. Copyright 2019 Elsevier.

**Figure 21:**
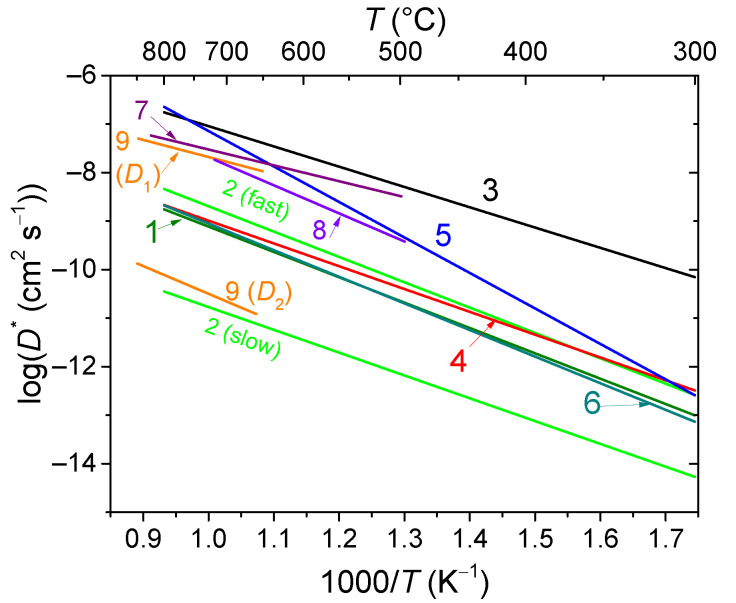
Arrhenius plots for oxygen tracer diffusion coefficient of first-order RP phases: 1—La_2_NiO_4+δ_ [291], 2—La_1.7_Ca_0.3_NiO_4+δ_ [291], 3—Pr_2_NiO_4+δ_ [299], 4—Pr_1.7_Ca_0.3_NiO_4+δ_ [299], 5—Nd_2_NiO_4+δ_ [300], 6—Nd_1.7_Ca_0.3_NiO_4+δ_ [300], 7—La_2_Ni_0.5_Cu_0.5_O_4+δ_ [286], 8—La_2_CuO_4+δ_ [286], 9—Pr_1.75_Sr_0.25_Ni_0.75_Co_0.25_O_4+δ_ [287].

The Ruddlesden–Popper (RP) phases with the general formula of (AO)(ABO_3_)_n_ or A_n+1_B_n_O_3n+1_ consist of the perovskite layers ABO_3−δ_ alternating with the rock salt layers A_2_O_2+δ_ [58,60,63,69,129,159,285,286,287,288,289,290]. The important feature of RP phases, which makes them attractive SOFC cathodes and oxygen-separation-membrane materials, is a fine oxygen transport provided via the cooperative mechanism of oxygen migration. In this case, both lattice and interstitial oxide anions accumulating at a high level are involved in the process of oxygen transport (Figure 20) [55,58,60,63,69,159,285,288,291,292,293,294,295,296,297]. This allows them to reach superior oxygen mobility compared to other MIECs (Figure 21). On the other hand, doping with alkaline earth metals (Ca, Sr, Ba), which significantly improves total conductivity, leads to an apparent decrease in the oxygen tracer diffusion coefficient values due to a decrease in the interstitial oxygen content and a larger size of dopant cations resulting in steric hindrances for the oxygen transport [287,288,292,298,299,300]. In some cases, it leads to the formation of slow diffusion channels with complicated pathways (Figure 21). The fraction of oxygen involved in the oxygen slow diffusion channel increases with the increasing cation-dopant radius in a row of Ca, Sr, Ba. With a decreasing host Ln cation size in the row of Ln = La, Pr, Nd, this effect becomes less pronounced. Introducing A-site deficiency can slightly increase oxygen diffusivity [55,69,292,301,302,303]. Doping La_2_NiO_4+δ_ with other lanthanides (Nd, Sm, Gd, Eu, etc.) can slightly increase or decrease the oxygen mobility as well [177,304]. The information on the effect of doping RP nickelates in the B-site with such cations as Cu on the oxygen transport properties is still lacking and controversial. The oxygen diffusivity can increase while doping with Cu due to the elongation of Ni/Cu–O bonds [305,306] and anomalous grain growth can occur due to Cu-rich liquid phase presence during sintering [307]; it can decrease due to decreasing the oxygen content [308,309], and a non-monotonous dependence can be observed as well [310].

**Figure 22:**
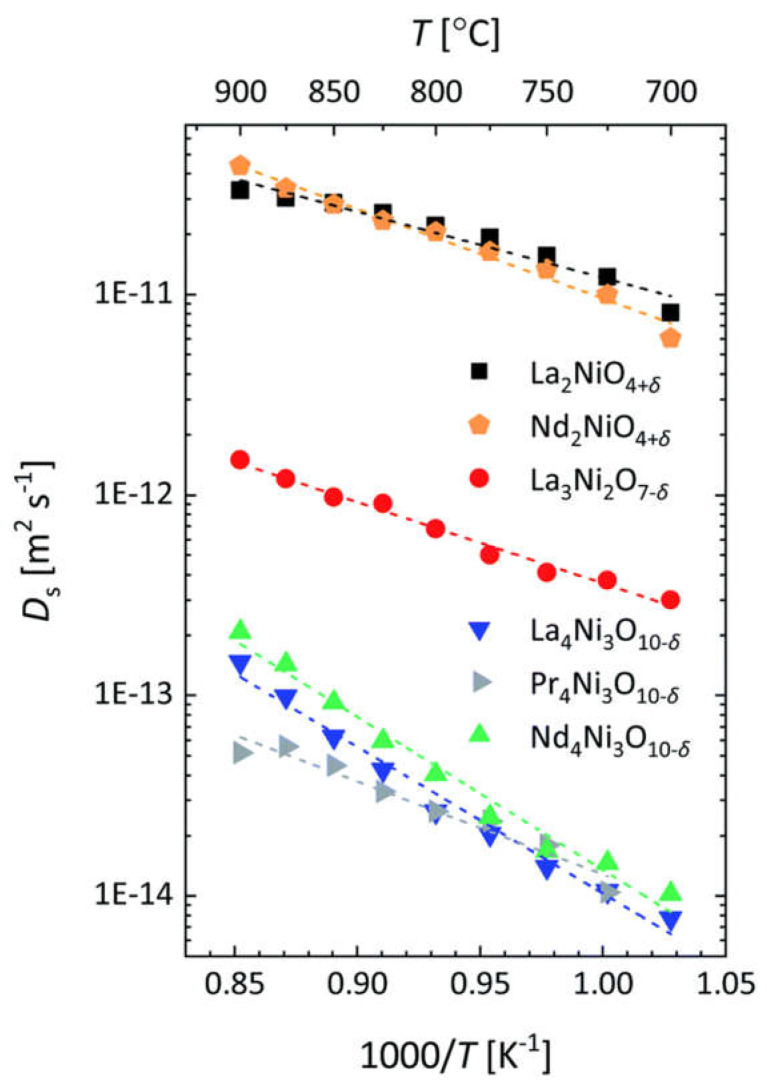
Arrhenius plots of the oxygen self-diffusion coefficient (*D_s_*) of RP nickelates [311]. Reprinted from ref. [311] under the CC BY-NC 3.0 license.

The RP phases of higher orders, different from the first-order ones being overstoichiometric and accumulating large amount of highly mobile interstitial oxygen, tend to be hypostoichiometric. Hence, they contain less amounts of interstitial oxygen in the rock salt layers and more oxygen vacancies in the perovskite layers. As a result, the oxygen diffusivity of the higher-order RP phases is lower compared to that of the first-order RP phases (Figure 22). For these materials, the contribution of the oxygen vacancy migration in the perovskite layers into the diffusion mechanism becomes predominant [108,288,311,312,313,314].

It was also reported [108] that some RP phases possess proton mobility, which results in accelerating the cathodic reaction process in H-SOFCs. Proton migration is believed to be implemented via the Grotthuss mechanism (Figure 8b). It includes two main pathways, namely, the inner-layer migration within the perovskite structure and the inter-layer migration between neighboring perovskite layers across the rock salt layer [108].

### 6.5. Composites

**Figure 23:**
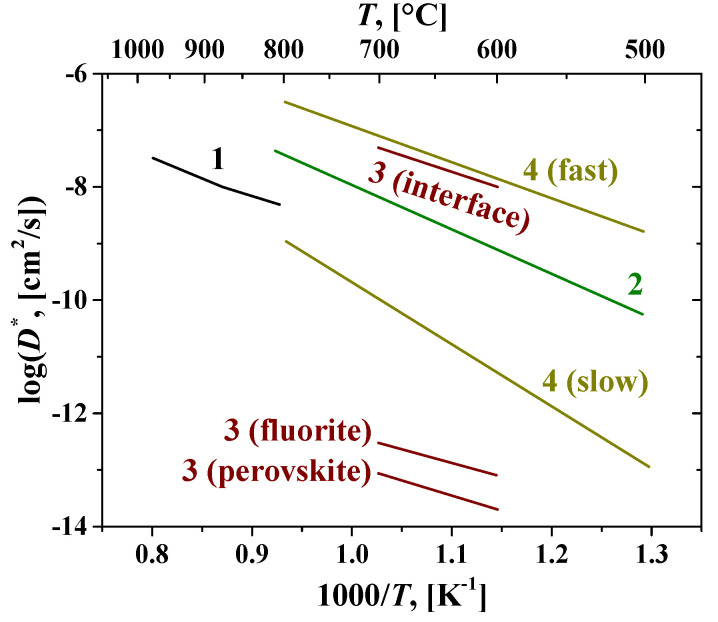
Arrhenius plots for the oxygen tracer diffusion coefficient for selected perovskite–fluorite nanocomposites: 1—LSM–YSZ [319], 2—LSFC–GDC [320], 3—LSFN–GDC [60], 4—PNC–YDC [321]. Reprinted with permission from Ref. [319]. Copyright 2022 Elsevier.

A promising approach in the design of materials for SOFC and permselective membranes is the synthesis of composites comprising both an electronic-conductive or MIEC material and an ionic conductive material [55,58,91,216,248,269,315]. The ionic-conductive component applied can be fluorite [55,60,244,316,317,318,319,320,321], pyrochlore [55,60,69], spinel [55,322], etc. Such an approach allows one to combine the features of the components, such as a high electronic conductivity of one component and a high ionic conductivity of another component, and even acquire new characteristics due to the synergetic effect of both phases, such as a fast oxide ionic transport across the interfaces or fast diffusion channels (Figure 23) [55,69,269]. For example, for PrNi_1−x_Co_x_O_3_–Ce_0.9_Y_0.1_O_1.95_ (x = 0.4–0.6), such a fast channel corresponds to the interfaces and the fluorite-like phase due to the redistribution of cations, namely, the incorporation of Pr^3+/4+^ cations into the doped ceria structure [55,60,69,269,321].

However, using composites not always results in improving all transport properties. Thus, for the composites based on Pr_1.9_NiO_4+δ_ and solid electrolytes (Ce_0.9_Gd_0.1_O_1.95_, Y_2_(Ti_0.8_Zr_0.2_)_1.6_Mn_0.4_O_7−δ_) a decrease in the oxygen tracer diffusion coefficient values compared to the individual Pr_1.9_NiO_4+δ_ material was reported, which is probably explained by the incorporation of the cations from the electrolyte into the RP structure leading to hampering the cooperative mechanism of oxygen migration mentioned above [58]. For the composites (Nd,La)_5.5_(W,M)O_11.25−δ_–Ni_0.5_Cu_0.5_O, a decrease in the oxygen tracer diffusion coefficient values compared to the individual defective fluorites was probably due to blocking the fluorite phase surface by Ni (II)–Cu (II) oxide nanoparticles possessing a low oxygen mobility, as well as the formation of admixture phases [216].

### 6.6. Other Materials

Some MIEC spinels such as Mn_x_Co_3−x_O_4_ [55,323], Fe_0.6_Mn_0.6_Co_0.6_Ni_0.6_Cr_0.6_O_4_ [324], and LaFe_2_O_4_ [225] can be utilized as cathode materials for SOFCs, including proton-conducting cells, due to a high activity in the oxygen reduction reaction (ORR). MnFe_2_O_4_ spinel and its composite with Gd-doped ceria are used for the fabrication of the oxygen-permeable protecting (buffer) layer of asymmetric supported oxygen separation membranes [53,55,56,69,321].

**Figure 24:**
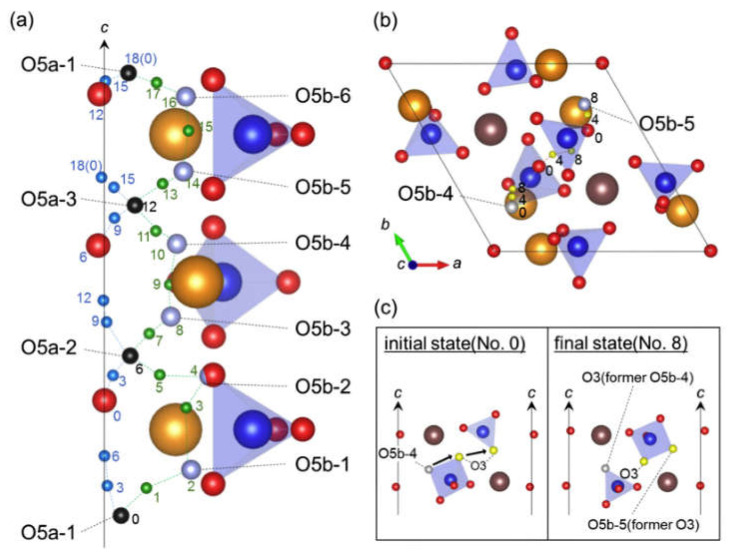
Cooperative oxygen transport mechanism in apatites [325]. (**a**) Two different conduction pathways along the c axis. The blue and green spheres represent trajectories of the interstitialcy and the interstitial mechanisms, respectively. The number beside each sphere corresponds to the image number in the calculated energy profiles. (**b**) A conduction pathway in the ab-plane. Yellow spheres represent trajectories of three O ions from O5b-4 to O5b-5. (**c**) Local atomic structures in the initial and final states of the pathway from O5b-4 to O5b-5. Reprinted with permission from Ref. [325]. Copyright 2020 Elsevier.

**Figure 25:**
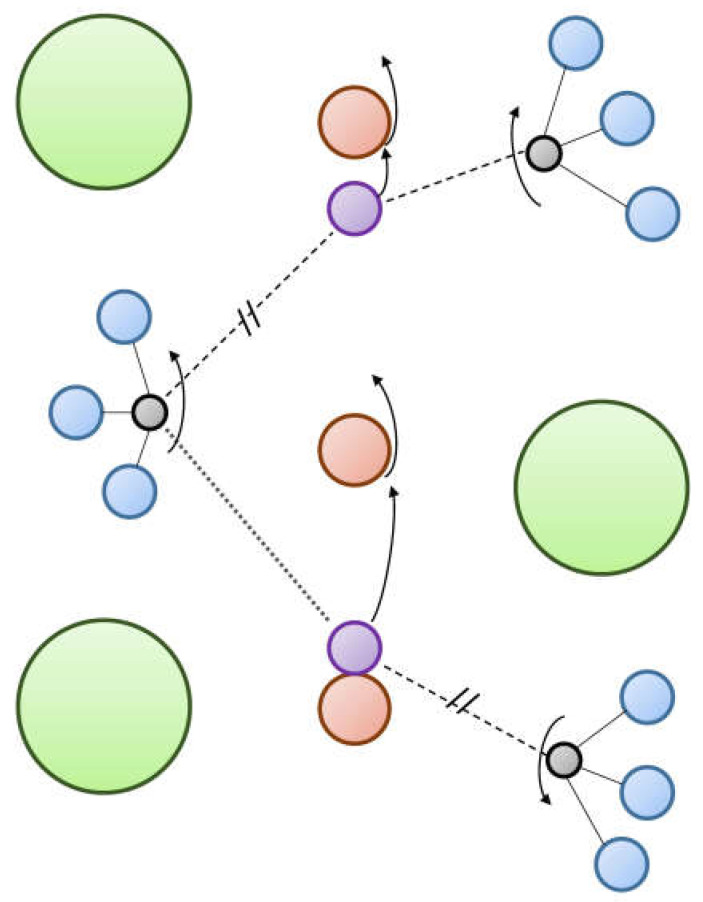
“Cog-wheel” cooperative mechanism of oxygen migration in La_1−x_Ba_1+x_GaO_4−x/2_ [134,326].

**Figure 26:**
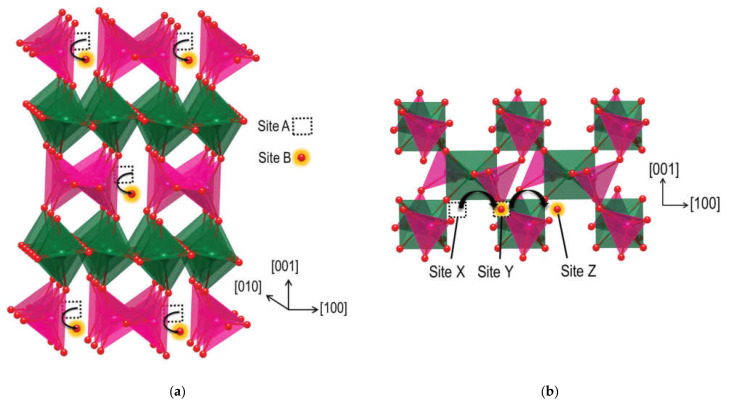
Cooperative mechanism of oxygen migration in SrCoO_2.5_ with brownmillerite structure. (**a**) Trajectory of interstitial oxygen migration through the vacancy channel within the tetrahedral layer from site *A* to site *B*. The interstitial oxygen atom moves towards the cobalt atom during its transport to site *B*. (**b**) Interstitial oxygen migration perpendicular to the vacancy channel along the *a* axis. Reprinted with permission from Ref. [328], Copyright 2014 AIP Publishing.

**Figure 27:**
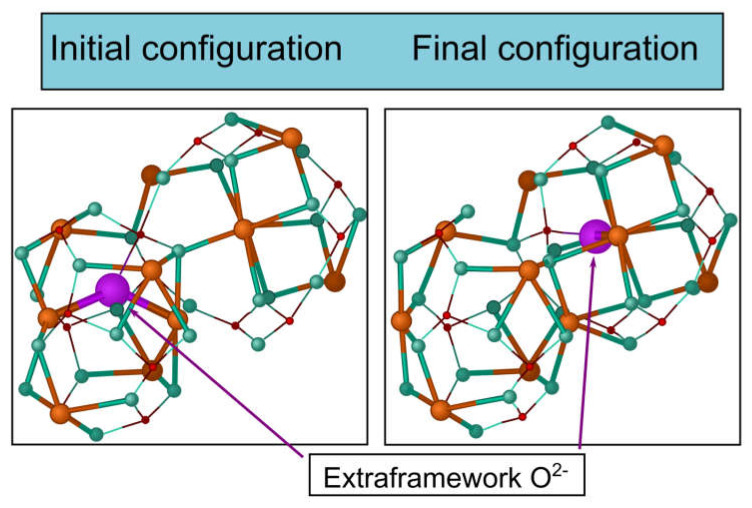
‘Free’ oxygen migration in mayenite [136]. Reprinted with permission from Ref. [136]. Copyright 2009 Elsevier.

**Figure 28:**
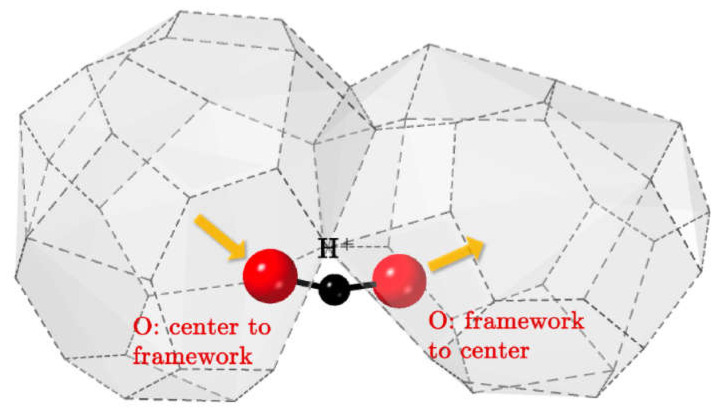
Transition state configuration in path along the pathway involving the nonbridging oxygen in the mayenite structure [335]. Reprinted with permission from Ref. [335]. Copyright 2020 American Chemical Society.

**Figure 29:**
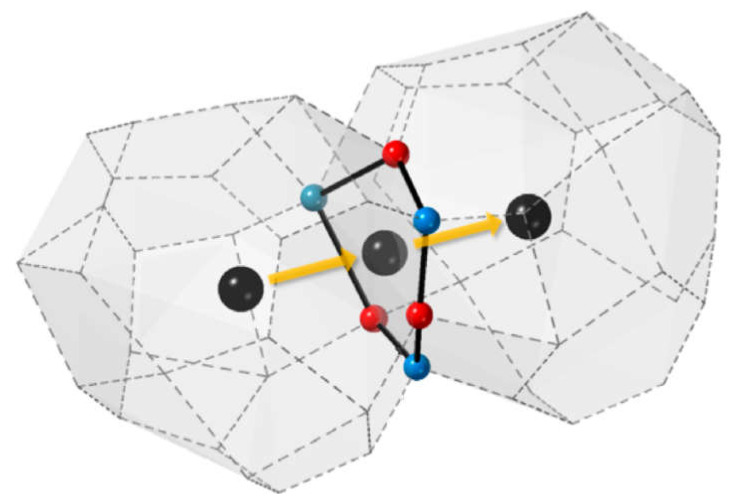
Hydrogen diffusion path in the mayenite structure and the transition state configuration. The intercage opening involved during hydrogen hopping as H^−^ hydride is highlighted [335]. Reprinted with permission from Ref. [335]. Copyright 2020 American Chemical Society.

Various types of oxide materials, which possess ionic conductivity due to cooperative oxygen migration mechanisms involving the cooperative motion of some forms of oxygen, can be used as SOFC electrolytes or, as a composite with electronically conductive or MIEC materials, as SOFC electrodes and oxygen separation membranes (or their permselective layers). Amongst these materials, doped La silicates/germanates with the apatite structure [58,69,107,134,325] (Figure 24), alkaline-earth-metal-doped La gallates with β-K_2_SO_4_ structure (Figure 25) [107,134,326], alkaline-earth metal ferrites, cobaltites, aluminates, gallates and indates with a brownmillerite structure (Figure 26) [107,134,327,328], M_3−x_M’_x_Ti_2_NbO_10−δ_ (M = Na, Ca, Cs; M = Bi, Ln, Rb) with a Dion–Jacobson-type layered perovskite structure [329,330,331], etc. [55,58,60,69] are to be mentioned. Mayenites based on Ca_12_Al_7_O_33,_ possessing a high oxygen mobility due to the fast transport of weakly bound intracellular ‘free’ oxygen (Figure 27), are to be mentioned as well [107,134,135,136,332]. Doping with Si mayenite possessing in general oxide-ionic type of conductivity allows one to increase the electronic conductivity, which is necessary for the cathode application [55,333]. It is to be noted that these materials, including apatites, brownmillerites, mayenites, etc., possess a high protonic conductivity [107,327,334,335]; hence, they can be used in H-SOFCs and hydrogen separation membranes as well. E.g., mayenites possess a high hydrogen diffusivity which is implemented by vehicle and Grotthuss mechanisms, including OH^−^ migration and the reorientation of O–H bonds to jump between neighboring oxygen species in (O–H–O)^3−^ transition states (Figure 28), as well as hydrogen jumps in a form of hydride H^−^ (Figure 29) and non-charged H^0^ [335].

Swedenborgite-like RBaCo_4−x_M_x_O_7_ (R = Y, Ca, In, Lu, Yb, etc., M = Co, Zn, Fe, Al, Ga) phases were demonstrated to be potential cathodes for low-temperature SOFCs due to their low thermal expansion and excellent electrochemical performance; however, their phase decomposition at elevated temperatures of 700–800 °C limited their application [60,244,336,337].

Other materials with low thermal expansion coefficient values, high total conductivity and fine oxygen transport properties to be mentioned as candidate SOFC cathodes are yttrium iron garnet Y_3_Fe_5_O_12_ [55,244,338], misfit layered Ca_3_Co_4_O_9_-based phases [339,340,341,342,343,344], and Aurivillius oxides (Bi_2_O_2_)(A_m−1_B_m_O_3m+1_) (A = Na^+^, K^+^, Ca^2+^, Sr^2+^, Pb^2+^, Bi^3+^, etc.; B = Ti^4+^, Nb^5+^, Ta^5+^, etc.) [345,346]. The Aurivillius oxide Bi_2_Sr_2_Nb_2_MnO_12−δ_ notably demonstrates an excellent chemical stability (including CO_2_ tolerance) as well. Ca_3_Co_4_O_9_ demonstrates fast surface-exchange kinetics (k* = 1.6 × 10^−7^ cm s^−1^ at 700 °C to be compared to 1.3 × 10^−7^ cm s^−1^ for the nickelate) [347], and is promising for air cathodes used in all type SOFCs, H-SOFCs and reversible cells, individually or in composites with protonics [348], ionics [349] and MIECs [350].

Alkaline-earth-metal-doped lanthanide niobates with sheelite, defective perovskite, monoclinic and tetragonal structures possess ionic (protonic and/or oxide-ionic), electronic or mixed ionic-electronic conductivity [58,351,352,353,354,355,356,357]. They can be used as a component of the composites for hydrogen separation membranes such as (La,Ca)NbO_4_–La_3_NbO_7_, (La,Ca)NbO_4_–LaNb_3_O_9_ and (La,Ca)NbO_4_–NiCu [58,351,352,353,354,355,356,357].

**Figure 30:**
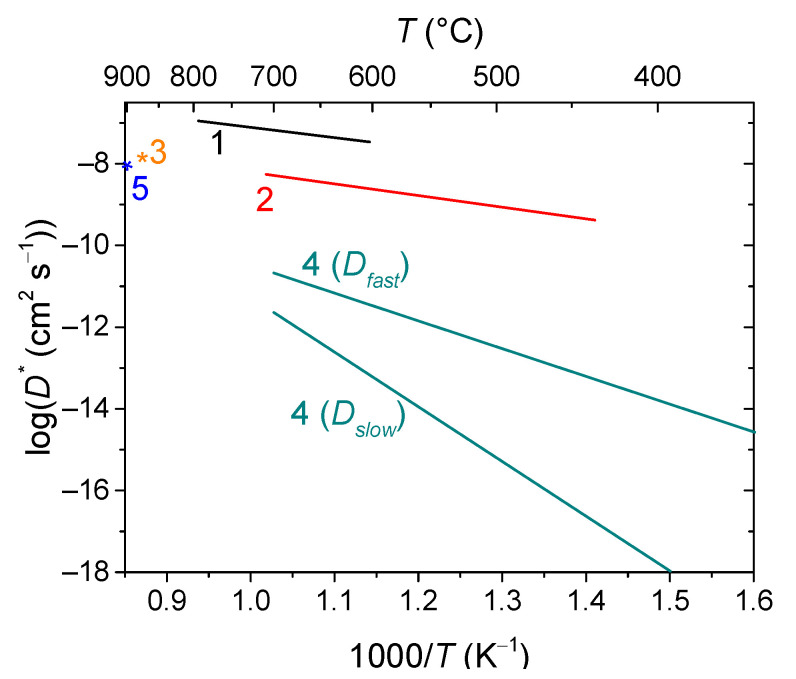
Arrhenius plots for oxygen tracer diffusion coefficient for various materials for SOFCs and permselective membranes: 1—PrBaCo_2_O_6−δ_ [280], 2—La_9.83_Si_5_Al_0.75_Fe_0.25_O_26.5_ [58], 3—Ca_12_Al_7_O_33_ [332], 4-La_0.99_Ca_0.01_NbO_4_—LaNb_3_O_9_ [356], 5—Y_3_Fe_5_O_12_ [338].

Figure 30 demonstrates the oxygen mobility of some non-conventional materials for SOFCs and permselective membranes.

**Figure 31:**
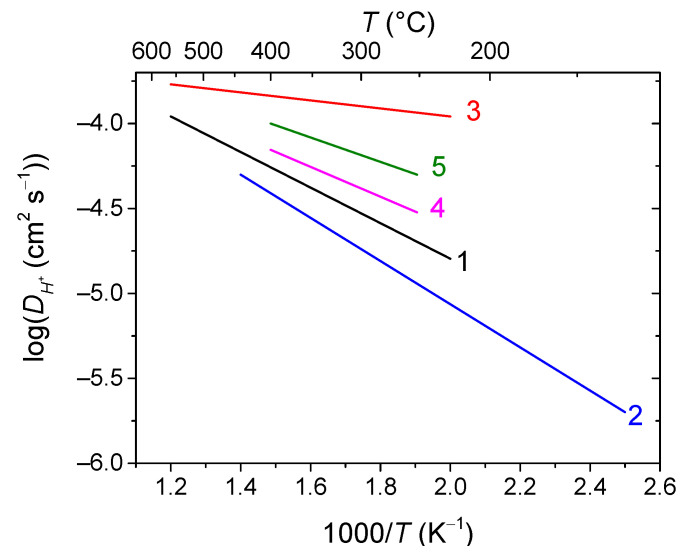
Arrhenius plots for hydrogen self-diffusion coefficient for various metals and alloys: 1—Pd [359], 2—Pd_0.77_Ag_0.23_ [358], 3—V [359], 4—V_0.85_Ni_0.15_ [359], 5—V_0.9_Cr_0.05_Al_0.05_ [359].

Metals and their alloys which can intercalate and transport protons as a defect (Figure 8a) are widely used for hydrogen separation membranes. Precious metals such as Pt, Pd, Ru, Ag and their alloys are conventionally used as hydrogen separation membrane materials. They possess absolute selectivity with respect to hydrogen; however, they are too expensive and have issues with stability under operating conditions [49,55,56,84,85,99,139,358]. As an alternative to precious metals, Ni and its alloys, being cheap but also possessing a high mixed protonic-electronic conductivity, can be used in hydrogen separation membranes in an individual form or as a component of cermet composites [55,56,132,216,359,360]. V and its alloys with Ni, Cu, V, Nb, Ta and other metals are promising materials showing high hydrogen permeation fluxes exceeding those for Pd-based membranes and having a lower cost [55,359,360,361]. The comparison of the hydrogen self-diffusion coefficient values of various metals and alloys is given in Figure 31.

## 7. Conclusions and Perspectives

In this review, the importance of oxygen and hydrogen mobility for the performance of solid oxide fuel cells, oxygen and hydrogen separation membranes was highlighted. Detailed studies of ionic transport characteristics using modern techniques such as temperature-programmed isotope exchange of oxygen with C^18^O_2_, ECR technique, etc., were shown to widen the possibility of the design of advanced materials for these applications. Analysis of the modern literature of isotope-exchange methods demonstrated the necessity of the further development of isotope-exchange techniques, including the usage of new labelled oxygen or hydrogen containing molecules to study the bulk diffusion and the surface exchange processes. The development of new approaches for such data analysis or improving the existing ones in combination with the data acquired by relaxation techniques, structural and spectroscopic methods will help to elucidate atomic-scale factors controlling the mechanisms of diffusion and surface exchange properties.

Moreover, a lack of data should be noted regarding the oxygen and hydrogen transport properties of many functionally attractive and promising materials for electrochemical devices with decreased operating temperature, which requires their further study. One of the interesting approaches in creating the materials for these devices is related to triple-conductive (H^+^ + O^2−^ + e^−^) oxides and composites, which may demonstrate better characteristics compared to the conventional mixed O^2−^ + e^−^ or H^+^ + e^−^ conductive materials. Studying the oxygen-transport properties of the proton conductors and vice versa would allow us to expand the known number of triple-conductive materials and find new applications for these materials. Selecting solid oxide fuel cell materials with a high oxygen and/or hydrogen mobility and surface reactivity allows to decrease the fuel cell operating temperature and increase its power density due to reducing the electrolyte resistance and enabling the electrode processes to take place on the electrode–gas phase double-phase boundary not to be limited by the electrode–electrolyte–gas phase triple-phase boundary. This opens new perspectives in the solid oxide fuel cells design and manufacturing.

Mixed ionic-electronic conducting materials for permselective membranes with a high oxygen and hydrogen mobility and surface reactivity, as well as a high electronic conductivity, allows the obtaining of high permeation fluxes of oxygen and hydrogen, respectively. Along with this, the presence of oxygen component of the conductivity of hydrogen separation membrane materials allows us to increase the hydrogen yield. This opens the opportunity for creating new membrane materials followed by their selection for the prospective practical use based on their superior transport properties.

## Figures and Tables

**Table 1:** Options of oxygen isotope exchange experiments with gas-phase analysis.

Reactor Type		
	Static	Flow
Oxygen exchanged in the oxide	<10%	up to ≅100%
Sensitivity to the diffusion rate	No	Yes
Low isotope consumption	Yes	No
Simple reactor construction	No	Yes
Exchangeable gas-phase reagent		
	^18^O_2_	C^18^O_2_
Sensitivity to the diffusion rate	No	Yes
Sensitivity to the kinetics of interaction with the oxide surface	Yes	No
Temperature mode		
	Isothermal	Temperature-programmed
Sensitivity to the oxygen non-uniformity in the oxide bulk	No	Yes

## Data Availability

Not applicable.

## References

[1] Rahman A., Farrok O., Haque M.M. (2022). Environmental Impact of Renewable Energy Source Based Electrical Power Plants: Solar, Wind, Hydroelectric, Biomass, Geothermal, Tidal, Ocean, and Osmotic. Renew. Sustain. Energy Rev..

[2] Abanades S., Abbaspour H., Ahmadi A., Das B., Ehyaei M.A., Esmaeilion F., Assad M.E.H., Hajilounezhad T., Hmida A., Rosen M.A. (2022). A Conceptual Review of Sustainable Electrical Power Generation from Biogas. Energy Sci. Eng..

[3] Ang T.-Z., Salem M., Kamarol M., Das H.S., Nazari M.A., Prabaharan N. (2022). A Comprehensive Study of Renewable Energy Sources: Classifications, Challenges and Suggestions. Energy Strat. Rev..

[4] Tian X., An C., Chen Z. (2023). The Role of Clean Energy in Achieving Decarbonization of Electricity Generation, Transportation, and Heating Sectors by 2050: A Meta-Analysis Review. Renew. Sustain. Energy Rev..

[5] Isazadeh A., Ziviani D., Claridge D.E. (2023). Global Trends, Performance Metrics, and Energy Reduction Measures in Datacom Facilities. Renew. Sustain. Energy Rev..

[6] Sayed E.T., Olabi A.G., Alami A.H., Radwan A., Mdallal A., Rezk A., Abdelkareem M.A. (2023). Renewable Energy and Energy Storage Systems. Energies.

[7] Wimalaratna Y.P., Afrouzi H.N., Mehranzamir K., Siddique M.B.M., Liew S.C., Ahmed J. (2022). Analysing Wind Power Penetration in Hybrid Energy Systems Based on Techno-Economic Assessments. Sustain. Energy Technol. Assess..

[8] Kamal M.M., Ashraf I. (2023). Evaluation of a Hybrid Power System Based on Renewable and Energy Storage for Reliable Rural Electrification. Renew. Energy Focus.

[9] Das P., Chandramohan V.P. (2023). A Review on Recent Advances in Hybrid Solar Updraft Tower Plants: Challenges and Future Aspects. Sustain. Energy Technol. Assess..

[10] Gomaa M.R., Al-Bawwat A.K., Al-Dhaifallah M., Rezk H., Ahmed M. (2023). Optimal Design and Economic Analysis of a Hybrid Renewable Energy System for Powering and Desalinating Seawater. Energy Rep..

[11] Ileberi G.R., Li P. (2023). Integrating Hydrokinetic Energy into Hybrid Renewable Energy System: Optimal Design and Comparative Analysis. Energies.

[12] Shokri A., Fard M.S. (2023). Water-Energy Nexus: Cutting Edge Water Desalination Technologies and Hybridized Renewable-Assisted Systems; Challenges and Future Roadmaps. Sustain. Energy Technol. Assess..

[13] Kuterbekov K.A., Nikonov A.V., Bekmyrza K.Z., Pavzderin N.B., Kabyshev A.M., Kubenova M.M., Kabdrakhimova G.D., Aidarbekov N. (2022). Classification of Solid Oxide Fuel Cells. Nanomaterials.

[14] Li N., Liu B., Jia L., Yan D., Li J. (2023). Liquid Biofuels for Solid Oxide Fuel Cells: A Review. J. Power Sources.

[15] Kazula S., de Graaf S., Enghardt L. (2023). Review of Fuel Cell Technologies and Evaluation of Their Potential and Challenges for Electrified Propulsion Systems in Commercial Aviation. J. Glob. Power Propuls. Soc..

[16] Peng J., Zhao D., Xu Y., Wu X., Li X. (2023). Comprehensive Analysis of Solid Oxide Fuel Cell Performance Degradation Mechanism, Prediction, and Optimization Studies. Energies.

[17] Damo U.M., Ferrari M.L., Turan A., Massardo A.F. (2019). Solid Oxide Fuel Cell Hybrid System: A Detailed Review of an Environmentally Clean and Efficient Source of Energy. Energy.

[18] Ma S., Lin M., Lin T.-E., Lan T., Liao X., Maréchal F., Van Herle J., Yang Y., Dong C., Wang L. (2021). Fuel Cell-Battery Hybrid Systems for Mobility and off-Grid Applications: A Review. Renew. Sustain. Energy Rev..

[19] Kumar P., Singh O. (2022). A Review of Solid Oxide Fuel Cell Based Hybrid Cycles. Int. J. Energy Res..

[20] Sinha A.A., Sanjay, Ansari M.Z., Shukla A.K., Choudhary T. (2023). Comprehensive Review on Integration Strategies and Numerical Modeling of Fuel Cell Hybrid System for Power & Heat Production. Int. J. Hydrogen Energy.

[21] Iliev I.K., Filimonova A.A., Chichirov A.A., Chichirova N.D., Pechenkin A.V., Vinogradov A.S. (2023). Theoretical and Experimental Studies of Combined Heat and Power Systems with SOFCs. Energies.

[22] He V., Gaffuri M., Van Herle J., Schiffmann J. (2023). Readiness Evaluation of SOFC-MGT Hybrid Systems with Carbon Capture for Distributed Combined Heat and Power. Energy Convers. Manag..

[23] Rahimi-Ahar Z., Hatamipour M.S. (2023). Exergy Analysis of Thermal Desalination Processes: A Review. Clean. Technol. Environ. Policy.

[24] Qin X., Cao J., Geng G., Li Y., Zheng Y., Zhang W., Yu B. (2022). Solid Oxide Fuel Cell System for Automobiles. Int. J. Green Energy.

[25] Abuadala A., Dincer I. (2012). A Review on Biomass-Based Hydrogen Production and Potential Applications: A Review on Biomass-Based Hydrogen Production and Applications. Int. J. Energy Res..

[26] Lee J., Lin K.-Y.A., Jung S., Kwon E.E. (2023). Hybrid Renewable Energy Systems Involving Thermochemical Conversion Process for Waste-to-Energy Strategy. Chem. Eng. J..

[27] Salimi M., Hosseinpour M., Mansouri S., Borhani T.N. (2022). Environmental Aspects of the Combined Cooling, Heating, and Power (CCHP) Systems: A Review. Processes.

[28] Tarancón A. (2009). Strategies for Lowering Solid Oxide Fuel Cells Operating Temperature. Energies.

[29] Skutina L., Filonova E., Medvedev D., Maignan A. (2021). Undoped Sr_2_MMoO_6_ Double Perovskite Molybdates (M = Ni, Mg, Fe) as Promising Anode Materials for Solid Oxide Fuel Cells. Materials.

[30] Li Z., Li M., Zhu Z. (2022). Perovskite Cathode Materials for Low-Temperature Solid Oxide Fuel Cells: Fundamentals to Optimization. Electrochem. Energy Rev..

[31] Kumar R.V., Khandale A.P. (2022). A Review on Recent Progress and Selection of Cobalt-Based Cathode Materials for Low Temperature-Solid Oxide Fuel Cells. Renew. Sustain. Energy Rev..

[32] Ahmad M.Z., Ahmad S.H., Chen R.S., Ismail A.F., Hazan R., Baharuddin N.A. (2022). Review on Recent Advancement in Cathode Material for Lower and Intermediate Temperature Solid Oxide Fuel Cells Application. Int. J. Hydrogen Energy.

[33] Pikalova E.Y., Kalinina E.G., Pikalova N.S., Filonova E.A. (2022). High-Entropy Materials in SOFC Technology: Theoretical Foundations for Their Creation, Features of Synthesis, and Recent Achievements. Materials.

[34] Tarutin A.P., Filonova E.A., Ricote S., Medvedev D.A., Shao Z. (2023). Chemical Design of Oxygen Electrodes for Solid Oxide Electrochemical Cells: A Guide. Sustain. Energy Technol. Assess..

[35] Filonova E., Pikalova E. (2023). Overview of Approaches to Increase the Electrochemical Activity of Conventional Perovskite Air Electrodes. Materials.

[36] Mathur L., Namgung Y., Kim H., Song S.-J. (2023). Recent Progress in Electrolyte-Supported Solid Oxide Fuel Cells: A Review. J. Korean Ceram. Soc..

[37] Hanif M.B., Rauf S., Motola M., Babar Z.U.D., Li C.-J., Li C.-X. (2022). Recent Progress of Perovskite-Based Electrolyte Materials for Solid Oxide Fuel Cells and Performance Optimizing Strategies for Energy Storage Applications. Mater. Res. Bull..

[38] Kim D., Jeong I., Kim K.J., Bae K.T., Kim D., Koo J., Yu H., Lee K.T. (2022). A Brief Review of Heterostructure Electrolytes for High-Performance Solid Oxide Fuel Cells at Reduced Temperatures. J. Korean Ceram. Soc..

[39] Maiti T.K., Majhi J., Maiti S.K., Singh J., Dixit P., Rohilla T., Ghosh S., Bhushan S., Chattopadhyay S. (2022). Zirconia- and Ceria-Based Electrolytes for Fuel Cell Applications: Critical Advancements toward Sustainable and Clean Energy Production. Environ. Sci. Pollut. Res..

[40] Choudhary B., Besra L., Anwar S., Anwar S. (2023). La_2_Ce_2_O_7_ Based Materials for next Generation Proton Conducting Solid Oxide Cells: Progress, Opportunity and Future Prospects. Int. J. Hydrogen Energy.

[41] Filonova E., Medvedev D. (2022). Recent Progress in the Design, Characterisation and Application of LaAlO_3_- and LaGaO_3_-Based Solid Oxide Fuel Cell Electrolytes. Nanomaterials.

[42] Yin H., Yip A.C.K. (2017). A Review on the Production and Purification of Biomass-Derived Hydrogen Using Emerging Membrane Technologies. Catalysts.

[43] Sun C., Alonso J.A., Bian J. (2021). Recent Advances in Perovskite-Type Oxides for Energy Conversion and Storage Applications. Adv. Energy Mater..

[44] Acharya D., Ng D., Xie Z. (2021). Recent Advances in Catalysts and Membranes for MCH Dehydrogenation: A Mini Review. Membranes.

[45] Al-Rowaili F.N., Khaled M., Jamal A., Zahid U. (2023). Mixed Matrix Membranes for H_2_/CO_2_ Gas Separation- a Critical Review. Fuel.

[46] Han N., Shen Z., Zhao X., Chen R., Thakur V.K. (2022). Perovskite Oxides for Oxygen Transport: Chemistry and Material Horizons. Sci. Total Environ..

[47] Meulenberg W.A., Schulze-Küppers F., Deibert W., Gestel T.V., Baumann S. (2019). Ceramic Membranes: Materials—Components—Potential Applications. ChemBioEng Rev..

[48] Wang Z., Chen T., Dewangan N., Li Z., Das S., Pati S., Li Z., Lin J.Y.S., Kawi S. (2020). Catalytic Mixed Conducting Ceramic Membrane Reactors for Methane Conversion. React. Chem. Eng..

[49] Algieri C., Coppola G., Mukherjee D., Shammas M.I., Calabro V., Curcio S., Chakraborty S. (2021). Catalytic Membrane Reactors: The Industrial Applications Perspective. Catalysts.

[50] Leo A., Liu S., Costa J.C.D.D. (2009). Development of Mixed Conducting Membranes for Clean Coal Energy Delivery. Int. J. Greenh. Gas Control.

[51] Gupta S., Mahapatra M.K., Singh P. (2015). Lanthanum Chromite Based Perovskites for Oxygen Transport Membrane. Mater. Sci. Eng. Rep..

[52] Deibert W., Ivanova M.E., Baumann S., Guillon O., Meulenberg W.A. (2017). Ion-Conducting Ceramic Membrane Reactors for High-Temperature Applications. J. Membr. Sci..

[53] Sadykov V.A., Sadovskaya E.M., Eremeev N.F., Pikalova E.Y., Bogdanovich N.M., Filonova E.A., Krieger T.A., Fedorova Y.E., Krasnov A.V., Skriabin P.I. (2020). Novel Materials for Solid Oxide Fuel Cells Cathodes and Oxygen Separation Membranes: Fundamentals of Oxygen Transport and Performance. Carbon Resour, Convers..

[54] Pikalova E.Y., Kalinina E.G. (2021). Solid Oxide Fuel Cells Based on Ceramic Membranes with Mixed Conductivity: Improving Efficiency. Russ. Chem. Rev..

[55] Sadykov V., Eremeev N., Sadovskaya E., Bespalko Y., Simonov M., Arapova M., Smal E. (2022). Nanomaterials with Oxygen Mobility for Catalysts of Biofuels Transformation into Syngas, SOFC and Oxygen/Hydrogen Separation Membranes: Design and Performance. Catal. Today.

[56] Sadykov V.A., Eremeev N.F., Sadovskaya E.M., Shlyakhtina A.V., Pikalova E.Y., Osinkin D.A., Yaremchenko A.A. (2022). Design of Materials for Solid Oxide Fuel Cells, Permselective Membranes, and Catalysts for Biofuel Transformation into Syngas and Hydrogen Based on Fundamental Studies of Their Real Structure, Transport Properties, and Surface Reactivity. Curr. Opin. Green Sustain. Chem..

[57] Adler S.B. (2004). Factors Governing Oxygen Reduction in Solid Oxide Fuel Cell Cathodes. Chem. Rev..

[58] Sadykov V.A., Sadovskaya E.M., Eremeev N.F., Skriabin P.I., Krasnov A.V., Bespalko Y.N., Pavlova S.N., Fedorova Y.E., Pikalova E.Y., Shlyakhtina A.V. (2019). Oxygen Mobility in the Materials for Solid Oxide Fuel Cells and Catalytic Membranes (Review). Russ. J. Electrochem..

[59] Adler S.B., Lane J.A., Steele B.C.H. (1996). Electrode Kinetics of Porous Mixed-Conducting Oxygen Electrodes. J. Electrochem. Soc..

[60] Sadykov V.A., Muzykantov V.S., Yeremeev N.F., Pelipenko V.V., Sadovskaya E.M., Bobin A.S., Fedorova Y.E., Amanbaeva D.G., Smirnova A.L. (2015). Solid Oxide Fuel Cell Cathodes: Importance of Chemical Composition and Morphology. Catal. Sustain. Energy.

[61] He S., Jiang S.P. (2021). Electrode/Electrolyte Interface and Interface Reactions of Solid Oxide Cells: Recent Development and Advances. Progr. Nat. Sci. Mater. Int..

[62] Mori T., Wepf R., Jiang S.P. (2020). Future Prospects for the Design of ‘State-of-the-Art’ Solid Oxide Fuel Cells. J. Phys. Energy.

[63] Manthiram A., Kim J.-H., Kim Y.N., Lee K.-T. (2011). Crystal Chemistry and Properties of Mixed Ionic-Electronic Conductors. J. Electroceram..

[64] van Eck N.J., Waltman L. (2010). Software Survey: VOSviewer, a Computer Program for Bibliometric Mapping. Scientometrics.

[65] Zhu W.Z., Deevi S.C. (2003). A Review on the Status of Anode Materials for Solid Oxide Fuel Cells. Mater. Sci. Eng. A.

[66] Shaikh S.P.S., Muchtar A., Somalu M.R. (2015). A Review on the Selection of Anode Materials for Solid-Oxide Fuel Cells. Renew. Sustain. Energy Rev..

[67] Tarancón A., Burriel M., Santiso J., Skinner S.J., Kilner J.A. (2010). Advances in Layered Oxide Cathodes for Intermediate Temperature Solid Oxide Fuel Cells. J. Mater. Chem..

[68] Pelosato R., Cordaro G., Stucchi D., Cristiani C., Dotelli G. (2015). Cobalt Based Layered Perovskites as Cathode Material for Intermediate Temperature Solid Oxide Fuel Cells: A Brief Review. J. Power Sources.

[69] Sadykov V.A., Mezentseva N.V., Bobrova L.N., Smorygo O.L., Eremeev N.F., Fedorova Y.E., Bespalko Y.N., Skriabin P.I., Krasnov A.V., Lukashevich A.I. (2019). Advanced Materials for Solid Oxide Fuel Cells and Membrane Catalytic Reactors. Advanced Nanomaterials for Catalysis and Energy.

[70] Zhu X., Yang W., Kharton V.V. (2017). Mixed Conducting Ceramic Membranes. Green Chemistry and Sustainable Technology.

[71] Zhao J., Pang Y., Su C., Jiang S., Ge L. (2023). Toward High Performance Mixed Ionic and Electronic Conducting Perovskite-Based Oxygen Permeable Membranes: An Overview of Strategies and Rationales. Energy Fuels.

[72] Athayde D.D., Motuzas J., Vasconcelos W. (2023). Perovskite Membranes for Oxygen Separation. Perovskite Ceramics.

[73] Ahmad F.N., Sazali N., Shalbi S., Ngadiman N.H.A., Othman M.H.D. (2019). Oxygen Separation Process Using Ceramic-Based Membrane: A Review. J. Adv. Res. Fluid Mechan. Therm. Sci..

[74] Xue J., Weng G., Chen L., Suo Y., Wei Y., Feldhoff A., Wang H. (2019). Various Influence of Surface Modification on Permeability and Phase Stability through an Oxygen Permeable Membrane. J. Membr. Sci..

[75] Li C., Li W., Chew J.J., Liu S., Zhu X., Sunarso J. (2019). Rate Determining Step in SDC-SSAF Dual-Phase Oxygen Permeation Membrane. J. Membr. Sci..

[76] Li C., Li W., Chew J.J., Liu S., Zhu X., Sunarso J. (2020). Oxygen Permeation through Single-Phase Perovskite Membrane: Modeling Study and Comparison with the Dual-Phase Membrane. Sep. Purif. Technol..

[77] Xu S.J., Thomson W.J. (1999). Oxygen Permeation Rates through Ion-Conducting Perovskite Membranes. Chem. Eng. Sci..

[78] Bouwmeester H.J.M., Burggraaf A.J. (1996). Chapter 10 Dense Ceramic Membranes for Oxygen Separation. Membrane Science and Technology.

[79] Shelepova E., Vedyagin A., Sadykov V., Mezentseva N., Fedorova Y., Smorygo O., Klenov O., Mishakov I. (2016). Theoretical and Experimental Study of Methane Partial Oxidation to Syngas in Catalytic Membrane Reactor with Asymmetric Oxygen-Permeable Membrane. Catal. Today.

[80] Wilkner K., Mücke R., Baumann S., Meulenberg W.A., Guillon O. (2022). Sensitivity of Material, Microstructure and Operational Parameters on the Performance of Asymmetric Oxygen Transport Membranes: Guidance from Modeling. Membranes.

[81] Qadir S., Ahsan M., Hussain A. (2023). Computational Fluid Dynamics Analysis of a Hollow Fiber Membrane Module for Binary Gas Mixture. Gases.

[82] Magrasó A., Haugsrud R. (2014). Effects of the La/W Ratio and Doping on the Structure, Defect Structure, Stability and Functional Properties of Proton-Conducting Lanthanum Tungstate La_28−x_W_4+x_O_54+δ_. A Review. J. Mater. Chem. A.

[83] Shlyakhtina A.V., Shcherbakova L.G. (2012). New Solid Electrolytes of the Pyrochlore Family. Russ. J. Electrochem..

[84] Manohar (2012). Development & Characterization of Ceramic Membranes. Int. J. Modern Eng. Res..

[85] Habib M.A., Harale A., Paglieri S., Alrashed F.S., Al-Sayoud A., Rao M.V., Nemitallah M.A., Hossain S., Hussien M., Ali A. (2021). Palladium-Alloy Membrane Reactors for Fuel Reforming and Hydrogen Production: A Review. Energy Fuels.

[86] Cheng H. (2022). Dual-Phase Mixed Protonic-Electronic Conducting Hydrogen Separation Membranes: A Review. Membranes.

[87] Vermaak L., Neomagus H.W.J.P., Bessarabov D.G. (2021). Recent Advances in Membrane-Based Electrochemical Hydrogen Separation: A Review. Membranes.

[88] Suzuki A., Yukawa H. (2020). A Review for Consistent Analysis of Hydrogen Permeability through Dense Metallic Membranes. Membranes.

[89] Liang W., Zhang Y., Hu T., Jiang H. (2021). Enhanced H_2_ Production by Using La_5.5_WO_11.25-δ_-La_0.8_Sr_0.2_FeO_3-δ_ Mixed Oxygen Ion-Proton-Electron Triple-Conducting Membrane. Int. J. Hydrogen Energy.

[90] Del-Pozo A., Villalobos J.C., Serna S. (2020). A General Overview of Hydrogen Embrittlement. Current Trends and Future Developments on (Bio-) Membranes.

[91] Hegde R.M., Kurkuri M.D., Kigga M., Inamuddin, Thomas S., Kumar Mishra R., Asiri A.M. (2019). Current Scenario of Nanocomposite Materials for Fuel Cell Applications. Sustainable Polymer Composites and Nanocomposites.

[92] Animitsa I., Neiman A., Sharafutdinov A., Nochrin S. (2000). Strontium Tantalates with Perovskite-Related Structure. Solid State Ion..

[93] Sunarso J., Hashim S.S., Zhu N., Zhou W. (2017). Perovskite Oxides Applications in High Temperature Oxygen Separation, Solid Oxide Fuel Cell and Membrane Reactor: A Review. Progr. Energy Combust. Sci..

[94] Escolástico S., Solís C., Haugsrud R., Magrasó A., Serra J.M. (2017). On the Ionic Character of H_2_ Separation through Mixed Conducting Nd_5.5_W_0.5_Mo_0.5_O_11.25−δ_ Membrane. Int. J. Hydrogen Energy.

[95] Papac M., Stevanović V., Zakutayev A., O’Hayre R. (2021). Triple Ionic–Electronic Conducting Oxides for next-Generation Electrochemical Devices. Nat. Mater..

[96] Virkar A. (2001). Transport of H2, O2 and H2O through Single-Phase, Two-Phase and Multi-Phase Mixed Proton, Oxygen Ion, and Electron Hole Conductors. Solid State Ion..

[97] Sanders M.D., O’Hayre R.P. (2011). Coupled Transport and Uphill Permeation of Steam and Oxygen in a Dense Ceramic Membrane. J. Membr. Sci..

[98] Liu L.C., Gong H.R., Zhou S.F., Gong X. (2019). Adsorption, Diffusion, and Permeation of Hydrogen at PdCu Surfaces. J. Membr. Sci..

[99] Cardoso S.P., Azenha I.S., Lin Z., Portugal I., Rodrigues A.E., Silva C.M. (2018). Inorganic Membranes for Hydrogen Separation. Sep. Purif. Rev..

[100] Huang Y., Zhang Q.-Y., Liao Q., Chen Y., Yan X., Guo X.-J., Lang W.-Z. (2021). Influence of Cr Doping on Hydrogen Permeation Performance of Lanthanum Tungstate Membrane. Sep. Purif. Technol..

[101] Norby T., Haugsrud R., Sammells A.F., Mundschau M.V. (2006). Dense Ceramic Membranes for Hydrogen Separation. Nonporous Inorganic Membranes.

[102] Kreuer K. (2000). On the Complexity of Proton Conduction Phenomena. Solid State Ion..

[103] Fontaine M., Norby T., Larring Y., Grande T., Bredesen R. (2008). Oxygen and Hydrogen Separation Membranes Based on Dense Ceramic Conductors. Membrane Science and Technology.

[104] Bobrova L., Vernikovskaya N., Eremeev N., Sadykov V. (2022). Model-Based Performance Analysis of Membrane Reactor with Ethanol Steam Reforming over a Monolith. Membranes.

[105] Bobrova L., Eremeev N., Vernikovskaya N., Sadykov V., Smorygo O. (2021). Effect of Asymmetric Membrane Structure on Hydrogen Transport Resistance and Performance of a Catalytic Membrane Reactor for Ethanol Steam Reforming. Membranes.

[106] Eremeev N., Krasnov A., Bespalko Y., Bobrova L., Smorygo O., Sadykov V. (2021). An Experimental Performance Study of a Catalytic Membrane Reactor for Ethanol Steam Reforming over a Metal Honeycomb Catalyst. Membranes.

[107] Malavasi L., Fisher C.A.J., Islam M.S. (2010). Oxide-Ion and Proton Conducting Electrolyte Materials for Clean Energy Applications: Structural and Mechanistic Features. Chem. Soc. Rev..

[108] Zhang L., Yao F., Meng J., Zhang W., Wang H., Liu X., Meng J., Zhang H. (2019). Oxygen Migration and Proton Diffusivity in Transition-Metal (Mn, Fe, Co, and Cu) Doped Ruddlesden–Popper Oxides. J. Mater. Chem. A.

[109] Shi H., Su C., Ran R., Cao J., Shao Z. (2020). Electrolyte Materials for Intermediate-Temperature Solid Oxide Fuel Cells. Progr. Nat. Sci. Mater. Int..

[110] Yang G., Jung W., Ahn S.-J., Lee D. (2019). Controlling the Oxygen Electrocatalysis on Perovskite and Layered Oxide Thin Films for Solid Oxide Fuel Cell Cathodes. Appl. Sci..

[111] Poetzsch D., Merkle R., Maier J. (2014). Proton Conductivity in Mixed-Conducting BSFZ Perovskite from Thermogravimetric Relaxation. Phys. Chem. Chem. Phys..

[112] Poetzsch D., Merkle R., Maier J. (2015). Stoichiometry Variation in Materials with Three Mobile Carriers-Thermodynamics and Transport Kinetics Exemplified for Protons, Oxygen Vacancies, and Holes. Adv. Funct. Mater..

[113] Poetzsch D., Merkle R., Maier J. (2015). Proton Uptake in the H^+^-SOFC Cathode Material Ba_0.5_Sr_0.5_Fe_0.8_Zn_0.2_O_3−δ_: Transition from Hydration to Hydrogenation with Increasing Oxygen Partial Pressure. Faraday Discuss..

[114] Salrin T.C., Johnson L., White S., Kilpatrick G., Weber E., Bragatto C. (2023). Using LAMMPS to Shed Light on Haven’s Ratio: Calculation of Haven’s Ratio in Alkali Silicate Glasses Using Molecular Dynamics. Front. Mater..

[115] Murch G.E. (1982). The Nernst-Einstein Equation in High-Defect-Content Solids. Philos. Magaz A.

[116] Akbar S.A. (1994). A Generalized View of the Correlation Factor in Solid-state Diffusion. J. Appl. Phys..

[117] Poirier D.R., Geiger G.H. (2016). Fick’s Law and Diffusivity of Materials. Transport Phenomena in Materials Processing.

[118] Goldberg E., Nemudry A., Boldyrev V., Schöllhorn R. (1998). Model for Anomalous Transport of Oxygen in Nonstoichiometric Perovskites: 1. General Formulation of the Problem. Solid State Ion..

[119] Goldberg E., Nemudry A., Boldyrev V., Schöllhorn R. (1999). Model for Anomalous Transport of Oxygen in Nonstoichiometric Perovskites Analytical and Numerical Solutions. Solid State Ion..

[120] Nemudry A., Rogatchev A., Gainutdinov I., Schöllhorn R. (2001). Reactivity of the Perovskite System Ca_1−x_Sr_x_FeO_2.5_ in Topotactic Electrochemical Oxidation at Ambient Temperature. J. Solid State Electrochem..

[121] Nemudry A., Goldberg E.L., Aguirre M., Alario-Franco M.Á. (2002). Electrochemical Topotactic Oxidation of Nonstoichiometric Perovskites at Ambient Temperature. Solid State Sci..

[122] Nemudry A., Uvarov N. (2006). Nanostructuring in Composites and Grossly Nonstoichiometric or Heavily Doped Oxides. Solid State Ion..

[123] Zhogin I.L., Nemudry A.P., Glyanenko P.V., Kamenetsky Y.M., Bouwmeester H.J.M., Ismagilov Z.R. (2006). Oxygen Diffusion in Nanostructured Perovskites. Catal. Today.

[124] Jin X., White R.E., Huang K. (2016). Simulating Charge Transport in Solid Oxide Mixed Ionic and Electronic Conductors: Nernst-Planck Theory vs Modified Fick’s Law. J. Electrochem. Soc..

[125] Mebane D.S., Liu Y., Liu M. (2007). A Two-Dimensional Model and Numerical Treatment for Mixed Conducting Thin Films. J. Electrochem. Soc..

[126] Lynch M.E., Liu M. (2010). Investigation of Sheet Resistance in Thin-Film Mixed-Conducting Solid Oxide Fuel Cell Cathode Test Cells. J. Power Sources.

[127] Liu M. (1997). Distributions of Charged Defects in Mixed Ionic-Electronic Conductors: I. General Equations for Homogeneous Mixed Ionic-Electronic Conductors. J. Electrochem. Soc..

[128] Lane J., Benson S., Waller D., Kilner J. (1999). Oxygen Transport in La_0.6_Sr_0.4_Co_0.2_Fe_0.8_O_3−δ_. Solid State Ion..

[129] Kilner J., De Souza R., Fullarton I. (1996). Surface Exchange of Oxygen in Mixed Conducting Perovskite Oxides. Solid State Ion..

[130] Honders A., Derkinderen J., Vanheeren A., Dewit J., Broers G. (1985). Bounded Diffusion in Solid Solution Electrode Powder Compacts. Part II. The Simultaneous Measurement of the Chemical Diffusion Coefficient and the Thermodynamic Factor in Li_x_TiS_2_ and Li_x_CoO_2_. Solid State Ion..

[131] Ishihara T. (2009). Perovskite Oxide for Solid Oxide Fuel Cells.

[132] Wimmer E., Wolf W., Sticht J., Saxe P., Geller C.B., Najafabadi R., Young G.A. (2008). Temperature-Dependent Diffusion Coefficients from *Ab Initio* Computations: Hydrogen, Deuterium, and Tritium in Nickel. Phys. Rev. B.

[133] De Larramendi I.R., Ortiz-Vitoriano N., Dzul-Bautista I.B., Rojo T., Pan L., Zhu G. (2016). Designing Perovskite Oxides for Solid Oxide Fuel Cells. Perovskite Materials Synthesis, Characterisation, Properties, and Applications.

[134] Chroneos A., Yildiz B., Tarancón A., Parfitt D., Kilner J.A. (2011). Oxygen Diffusion in Solid Oxide Fuel Cell Cathode and Electrolyte Materials: Mechanistic Insights from Atomistic Simulations. Energy Environ. Sci..

[135] Traqueia L.S.M., Marques F.M.B., Kharton V.V. (2006). Oxygen ion conduction in oxide materials: Selected examples and basic mechanisms. Bol. Soc. Cerám..

[136] Hosono H., Hayashi K., Kajihara K., Sushko P.V., Shluger A.L. (2009). Oxygen Ion Conduction in 12CaO·7Al_2_O_3_: O^2−^ Conduction Mechanism and Possibility of O^−^ Fast Conduction. Solid State Ion..

[137] Shlyakhtina A.V., Belov D.A., Knotko A.V., Avdeev M., Kolbanev I.V., Vorobieva G.A., Karyagina O.K., Shcherbakova L.G. (2014). Oxide Ion Transport in (Nd_2−x_Zr_x_)Zr_2_O_7+δ_ Electrolytes by an Interstitial Mechanism. J. Alloys Compd..

[138] Jacobson A.J. (2010). Materials for Solid Oxide Fuel Cells. Chem. Mater..

[139] Lundin S.T.B., Patki N.S., Fuerst T.S., Ricote S., Wolden S.A., Way J.D., Carreon M.A. (2017). Dense Inorganic Membranes for Hydrogen Separation. Membranes for Gas Separations.

[140] Ueki T., Watanabe M. (2008). Macromolecules in Ionic Liquids: Progress, Challenges, and Opportunities. Macromolecules.

[141] Colomban P. (2023). Vibrational Characterization of the Various Forms of (Solvated or Unsolvated) Mobile Proton in the Solid State. Advantages, Limitations and Open Questions. Solid State Ion..

[142] Boreskov G.K., Muzykantov V.S. (1973). Investigation of oxide-type oxidation catalysts by reactions of oxygen isotopic exchange. Ann. N. Y. Acad. Sci..

[143] Muzykantov V.S. (1987). Isotopic Studies of Dioxygen Activation on Oxide Catalysts for Oxidation: Problems, Results and Perspectives. React. Kinet. Catal. Lett..

[144] Busca G., Lorenzelli V. (1982). Infrared Spectroscopic Identification of Species Arising from Reactive Adsorption of Carbon Oxides on Metal Oxide Surfaces. Mater. Chem..

[145] Boreskov G.K., Kasatkina L.A., Amerikov V.G. (1969). Homomolecular Isotope Exchange of CO_2_ on Metal Oxides of the IV Period. Kinet. Catal..

[146] Muzykantov V.S., Cheshkova K.T., Boreskov G.K. (1973). Heteroexchange and Self-Diffusion of Oxygen in the O_2_–CO_2_–MoO_3_ System. Kinet. Catal..

[147] Gorelov V.P., Kurumchin E.K. (1986). Investigation of the Exchange of Cerium Dioxide by Isotopic Exchange with Molecular Oxygen. Kinet. Catal..

[148] Gorelov V.P., Kurumchin E.K. (1993). Investigation of the Exchange of Cerium Dioxide by Isotopic Exchange with Carbon Dioxide. Solid State Ionics..

[149] Amerikov V.G., Boreskov G.K., Kasatkina L.A. (1967). Catalytic Activity of Iron, Cobalt and Nickel Oxides with Respect to the Reaction of Isotope Exchange of Carbon Dioxide Molecules. Kinet. Catal..

[150] Uxa D., Dörrer L., Schulz M., Knoblauch N., Fielitz P., Roeb M., Schmücker M., Borchardt G. (2022). Investigation of CO_2_ Splitting on Ceria-Based Redox Materials for Low-Temperature Solar Thermochemical Cycling with Oxygen Isotope Exchange Experiments. Processes.

[151] Kasatkina L.A., Nekipelov V.N., Zhivotenko N.N. (1973). Reaction of Isotope Exchange of Carbon Monoxide on Fe_3_O_4_. Kinet. Catal..

[152] Tenelshof J., Bouwmeester H., Verweij H. (1996). Oxygen Transport through La_1−x_Sr_x_FeO_3−δ_ Membranes II. Permeation in Air/CO, CO Gradients. Solid State Ion..

[153] Xu X., Mace B., Enriquez E., Bao S., Harrell Z., Chen C., Whangbo M.-H. (2017). Roles of Reaction Kinetics of CO_2_ on a PrBaCo_2_O_5.5+δ_ Surfaces. RSC Adv..

[154] Bachiller-Baeza B., Rodriguez-Ramos I., Guerrero-Ruiz A. (1998). Interaction of Carbon Dioxide with the Surface of Zirconia Polymorphs. Langmuir.

[155] Bonhoeffer K.F., Farkas A. (1932). On Adsorption and Reflection Processes in the Interaction of Hydrogen and Metals. Trans. Faraday Soc..

[156] Rideal E.K. (1939). A Note on a Simple Molecular Mechanism for Heterogeneous Catalytic Reactions. Math. Proc. Camb. Philos. Soc..

[157] Eley D.D. (1948). The Absolute Rate of Conversion of Parahydrogen by Metallic Catalysts. Trans. Faraday Soc..

[158] Kim S., Wang S., Chen X., Yang Y.L., Wu N., Ignatiev A., Jacobson A.J., Abeles B. (2000). Oxygen Surface Exchange in Mixed Ionic Electronic Conductors: Application to La_0.5_Sr_0.5_Fe_0.8_Ga_0.2_O_3−δ_. J. Electrochem. Soc..

[159] Burriel M., Garcia G., Santiso J., Kilner J.A., Chater R.J., Skinner S.J. (2008). Anisotropic Oxygen Diffusion Properties in Epitaxial Thin Films of La_2_NiO_4+δ_. J. Mater. Chem..

[160] Porotnikova N.M., Khodimchuk A.V., Zakharov D.M., Bogdanovich N.M., Osinkin D.A. (2023). Enhancement of Surface Exchange and Oxygen Diffusion of Sr_1.95_Fe_1.4_Ni_0.1_Mo_0.5_O_6–δ_ Oxide Determined by Two Independent Isotope Exchange Methods. App. Surf. Sci..

[161] Sadykov V.A., Sadovskaya E.M., Uvarov N.F. (2015). Methods of Isotopic Relaxations for Estimation of Oxygen Diffusion Coefficients in Solid Electrolytes and Materials with Mixed Ionic-Electronic Conductivity. Russ. J. Electrochem..

[162] Pikalova E., Sadykov V., Sadovskaya E., Yeremeev N., Kolchugin A., Shmakov A., Vinokurov Z., Mishchenko D., Filonova E., Belyaev V. (2021). Correlation between Structural and Transport Properties of Ca-Doped La Nickelates and Their Electrochemical Performance. Crystals.

[163] Sadykov V., Shlyakhtina A., Sadovskaya E., Eremeev N., Skazka V., Goncharov V. (2020). 2D Diffusion of Oxygen in Ln_10_Mo_2_O_21_ (Ln = Nd, Ho) Oxides. Solid State Ion..

[164] Sadykov V., Shlyakhtina A., Lyskov N., Sadovskaya E., Cherepanova S., Eremeev N., Skazka V., Goncharov V., Kharitonova E. (2020). Oxygen Diffusion in Mg-Doped Sm and Gd Zirconates with Pyrochlore Structure. Ionics.

[165] Muzykantov V.S., Popovskii V.V., Boreskov G.K. (1964). Kinetics of Isotope Exchange in a Molecular Oxygen—Solid Oxide System. Kinet. Catal..

[166] Ananyev M.V., Zakharov D.M. (2020). H/D Isotopic Exchange between Methane and a Proton-Conducting Oxide: Theory and Experiment. Catal. Sci. Technol..

[167] Zakharov D.M., Zhuravlev N.A., Denisova T.A., Belozerov A.S., Stroeva A.Y., Vovkotrub E.G., Farlenkov A.S., Ananyev M.V. (2021). Catalytic Methane Activation over La_1−x_Sr_x_ScO_3−α_ Proton-Conducting Oxide Surface: A Comprehensive Study. J. Catal..

[168] Huang Y., Qiu R., Lian W., Lei L., Liu T., Zhang J., Wang Y., Liu J., Huang J., Chen F. (2022). Review: Measurement of Partial Electrical Conductivities and Transport Numbers of Mixed Ionic-Electronic Conducting Oxides. J. Power Sources.

[169] Zhao L., Dou B., Zhang H., Wang Z. (2021). Oxygen Carriers for Chemical-Looping Water Splitting to Hydrogen Production: A Critical Review. Carbon Capture Sci. Technol..

[170] Zhang C., Sunarso J., Liu S. (2017). Designing CO_2_-Resistant Oxygen-Selective Mixed Ionic–Electronic Conducting Membranes: Guidelines, Recent Advances, and Forward Directions. Chem. Soc. Rev..

[171] Hu B., Wang Y., Zhu Z., Xia C., Bouwmeester H.J.M. (2015). Measuring Oxygen Surface Exchange Kinetics on Mixed-Conducting Composites by Electrical Conductivity Relaxation. J. Mater. Chem. A.

[172] He F., Jin X., Tian T., Ding H., Green R.D., Xue X. (2015). Determination of Electrochemical Kinetic Property for Mixed Ionic Electronic Conductors from Electrical Conductivity Relaxation Measurements. J. Electrochem. Soc..

[173] Søgaard M., Hendriksen P.V., Mogensen M. (2007). Oxygen Nonstoichiometry and Transport Properties of Strontium Substituted Lanthanum Ferrite. J. Solid State Chem..

[174] Ovtar S., Søgaard M., Norrman K., Hendriksen P.V. (2018). Oxygen Exchange and Transport in (La_0.6_Sr_0.4_)_0.98_FeO_3−d_–Ce_0.9_Gd_0.1_O_1.95_ Dual-Phase Composites. J. Electrochem. Soc..

[175] He F., Jiang Y., Ren C., Dong G., Gan Y., Lee M., Green R.D., Xue X. (2016). Generalized Electrical Conductivity Relaxation Approach to Determine Electrochemical Kinetic Properties for MIECs. Solid State Ion..

[176] Seo H.G., Tuller H.L. (2023). Surface Oxygen Exchange Kinetics of Mixed Conducting Oxides: Dilatometric vs Electrical Conductivity Relaxation Study. Scr. Mater..

[177] Sadykov V.A., Sadovskaya E.M., Filonova E.A., Eremeev N.F., Belyaev V.D., Tsvinkinberg V.A., Pikalova E.Y. (2020). Oxide Ionic Transport Features in Gd-Doped La Nickelates. Solid State Ion..

[178] Seong A., Jeong D., Kim M., Choi S., Kim G. (2022). Performance Comparison of Composite Cathode: Mixed Ionic and Electronic Conductor and Triple Ionic and Electronic Conductor with BaZr_0.1_Ce_0.7_Y_0.1_Yb_0.1_O_3-δ_ for Highly Efficient Protonic Ceramic Fuel Cells. J. Power Sources.

[179] Sadykov V., Okhlupin Y., Yeremeev N., Vinokurov Z., Shmakov A., Belyaev V., Uvarov N., Mertens J. (2014). In Situ X-Ray Diffraction Studies of Pr_2−x_NiO_4+δ_ Crystal Structure Relaxation Caused by Oxygen Loss. Solid State Ion..

[180] Crank J. (1975). The Mathematics of Diffusion.

[181] Otter M.D., van der Haar L., Bouwmeester H. (2000). Numerical Evaluation of Eigenvalues of the Sheet Diffusion Problem in the Surface/Diffusion Mixed Regime. Solid State Ion..

[182] Otter M.W.D., Bouwmeester H.J.M., Boukamp B.A., Verweij H. (2001). Reactor Flush Time Correction in Relaxation Experiments. J. Electrochem. Soc..

[183] Sun Z., Fabbri E., Bi L., Traversa E. (2011). Lowering Grain Boundary Resistance of BaZr_0.8_Y_0.2_O_3−δ_ with LiNO_3_ Sintering-Aid Improves Proton Conductivity for Fuel Cell Operation. Phys. Chem. Chem. Phys..

[184] Sun W., Liu M., Liu W. (2013). Chemically Stable Yttrium and Tin Co-Doped Barium Zirconate Electrolyte for Next Generation High Performance Proton-Conducting Solid Oxide Fuel Cells. Adv. Energy Mater..

[185] Fabbri E., Bi L., Tanaka H., Pergolesi D., Traversa E. (2011). Chemically Stable Pr and Y Co-Doped Barium Zirconate Electrolytes with High Proton Conductivity for Intermediate-Temperature Solid Oxide Fuel Cells. Adv. Funct. Mater..

[186] Zvonareva I., Fu X.-Z., Medvedev D., Shao Z. (2022). Electrochemistry and Energy Conversion Features of Protonic Ceramic Cells with Mixed Ionic-Electronic Electrolytes. Energy Environ. Sci..

[187] Danilov N., Pikalova E., Lyagaeva J., Antonov B., Medvedev D., Demin A., Tsiakaras P. (2017). Grain and Grain Boundary Transport in BaCe_0.5_Zr_0.3_Ln_0.2_O_3−δ_ (Ln–Y or Lanthanide) Electrolytes Attractive for Protonic Ceramic Fuel Cells Application. J. Power Sources.

[188] Omata T., Otsuka-Yao-Matsuo S. (2001). Electrical Properties of Proton-Conducting Ca^2+^-Doped La_2_Zr_2_O_7_ with a Pyrochlore-Type Structure. J. Electrochem. Soc..

[189] Labrincha J.A., Frade J.R., Marques F.M.B. (1997). Protonic Conduction in La_2_Zr_2_O_7_-Based Pyrochlore Materials. Solid State Ion..

[190] Besikiotis V., Knee C.S., Ahmed I., Haugsrud R., Norby T. (2012). Crystal Structure, Hydration and Ionic Conductivity of the Inherently Oxygen-Deficient La_2_Ce_2_O_7_. Solid State Ion..

[191] Shimura T., Fujimoto S., Iwahara H. (2001). Proton Conduction in Non-Perovskite-Type Oxides at Elevated Temperatures. Solid State Ion..

[192] Seeger J., Ivanova M.E., Meulenberg W.A., Sebold D., Stöver D., Scherb T., Schumacher G., Escolástico S., Solís C., Serra J.M. (2013). Synthesis and Characterization of Nonsubstituted and Substituted Proton-Conducting La_6–*x*_WO_12–*y*_. Inorg. Chem..

[193] Escolástico S., Vert V.B., Serra J.M. (2009). Preparation and Characterization of Nanocrystalline Mixed Proton−Electronic Conducting Materials Based on the System Ln_6_WO_12_. Chem. Mater..

[194] Fantin A., Scherb T., Seeger J., Schumacher G., Gerhards U., Ivanova M.E., Meulenberg W.A., Dittmeyer R., Banhart J. (2017). Relation between Composition and Vacant Oxygen Sites in the Mixed Ionic-Electronic Conductors La_5.4_W_1−y_M_y_O_12−δ_ (M= Mo, Re; 0 ≤ y ≤ 0.2) and Their Mother Compound La_6−x_WO_12−δ_ (0.4 ≤ x ≤ 0.8). Solid State Ion..

[195] Marcano D., Ivanova M.E., Mauer G., Sohn Y.J., Schwedt A., Bram M., Menzler N.H., Vaßen R. (2019). PS-PVD Processing of Single-Phase Lanthanum Tungstate Layers for Hydrogen-Related Applications. J. Therm. Spray Technol..

[196] Partin G.S., Korona D.V., Neiman A.Y., Belova K.G. (2015). Conductivity and Hydration of Fluorite-Type La_6−x_WO_12−1.5x_ Phases (x = 0.4; 0.6; 0.8; 1). Russ. J. Electrochem..

[197] Savvin S.N., Shlyakhtina A.V., Kolbanev I.V., Knotko A.V., Belov D.A., Shcherbakova L.G., Nuñez P. (2014). Zr-Doped Samarium Molybdates—Potential Mixed Electron–Proton Conductors. Solid State Ion..

[198] Savvin S.N., Shlyakhtina A.V., Borunova A.B., Shcherbakova L.G., Ruiz-Morales J.C., Núñez P. (2015). Crystal Structure and Proton Conductivity of Some Zr-Doped Rare-Earth Molybdates. Solid State Ion..

[199] López-Vergara A., Porras-Vázquez J.M., Vøllestad E., Canales-Vazquez J., Losilla E.R., Marrero-López D. (2018). Metal-Doping of La_5.4_MoO_11.1_ Proton Conductors: Impact on the Structure and Electrical Properties. Inorg. Chem..

[200] López-Vergara A., Bergillos-Ruiz M., Zamudio-García J., Porras-Vázquez J.M., Canales-Vazquez J., Marrero-López D., Losilla E.R. (2020). Synergic Effect of Metal and Fluorine Doping on the Structural and Electrical Properties of La_5.4_MoO_11.1_ -Based Materials. Inorg. Chem..

[201] Shlyakhtina A.V., Savvin S.N., Lyskov N.V., Belov D.A., Shchegolikhin A.N., Kolbanev I.V., Karyagina O.K., Chernyak S.A., Shcherbakova L.G., Núñez P. (2017). Sm_6−x_MoO_12-δ_ (x = 0, 0.5) and Sm_6_WO_12_—Mixed Electron-Proton Conducting Materials. Solid State Ion..

[202] Shlyakhtina A.V., Savvin S.N., Lyskov N.V., Kolbanev I.V., Karyagina O.K., Chernyak S.A., Shcherbakova L.G., Núñez P. (2017). Polymorphism in the Family of Ln_6−x_MoO_12−δ_ (Ln = La, Gd–Lu; x = 0, 0.5) Oxygen Ion- and Proton-Conducting Materials. J. Mater. Chem. A.

[203] López-Vergara A., Porras-Vázquez J.M., Infantes-Molina A., Canales-Vázquez J., Cabeza A., Losilla E.R., Marrero-López D. (2017). Effect of Preparation Conditions on the Polymorphism and Transport Properties of La_6–*x*_MoO_12−δ_ (0 ≤ *x* ≤ 0.8). Chem. Mater..

[204] Shlyakhtina A.V., Kolbanev I.V., Degtyarev E.N., Lyskov N.V., Karyagina O.K., Chernyak S.A., Shcherbakova L.G. (2018). Kinetic Aspects of the Synthesis of Ln_6−х_MoO_12−δ_ (Ln = Sm, Ho -Yb; x = 0, 0.5) Rare-Earth Molybdates Using Mechanical Activation of Oxides. Solid State Ion..

[205] Savvin S.N., Avdeev M., Kolbanev I.V., Kharitonova E.P., Shcherbakova L.G., Shlyakhtina A.V., Nuñez P. (2018). Stability against Reduction of Fluorite-like Rhombohedral La_5.5_MoO_11.25_ and Ho_5.4_Zr_0.6_MoO_12.3_ Fluorite: Conductivity and Neutron Diffraction Study. Solid State Ion..

[206] Shlyakhtina A.V., Lyskov N.V., Avdeev M., Goffman V.G., Gorshkov N.V., Knotko A.V., Kolbanev I.V., Karyagina O.K., Maslakov K.I., Shcherbakova L.G. (2019). Comparative Study of Electrical Conduction and Oxygen Diffusion in the Rhombohedral and Bixbyite Ln_6_MoO_12_ (Ln = Er, Tm, Yb) Polymorphs. Inorg. Chem..

[207] Shlyakhtina A.V., Avdeev M., Abrantes J.C.C., Gomes E., Lyskov N.V., Kharitonova E.P., Kolbanev I.V., Shcherbakova L.G. (2019). Structure and Conductivity of Nd_6_MoO_12_-Based Potential Electron–Proton Conductors under Dry and Wet Redox Conditions. Inorg. Chem. Front..

[208] Denisova K., Shlyakhtina A., Yumashev O., Avdeev M., Abdel-Hafiez M., Volkova O., Vasiliev A. (2019). Low Temperature Thermodynamics of Yb_6_MoO_12_ and Lu_6_MoO_12_. J. Alloys Compd..

[209] López-Vergara A., Vizcaíno-Anaya L., Porras-Vázquez J.M., Baldinozzi G., Santos-Gómez L.D., Canales-Vazquez J., Marrero-López D., Losilla E.R. (2020). Unravelling Crystal Superstructures and Transformations in the La_6−*x*_MoO_12−δ_ (0.6 ≤ *x* ≤ 3.0) Series: A System with Tailored Ionic/Electronic Conductivity. Chem. Mater..

[210] Shlyakhtina A.V., Avdeev M., Lyskov N.V., Abrantes J.C.C., Gomes E., Denisova K.N., Kolbanev I.V., Chernyak S.A., Volkova O.S., Vasiliev A.N. (2020). Structure, Conductivity and Magnetism of Orthorhombic and Fluorite Polymorphs in MoO_3_–Ln_2_O_3_ (Ln = Gd, Dy, Ho) Systems. Dalton Trans..

[211] Shlyakhtina A.V., Lyskov N.V., Kolbanev I.V., Shchegolikhin A.N., Karyagina O.K., Shcherbakova L.G. (2021). Key Trends in the Proton Conductivity of Ln_6−x_MoO_12−δ_ (Ln = La, Nd, Sm, Gd -Yb; x = 0, 0.5, 0.6, 0.7, 1) Rare-Earth Molybdates. Int. J. Hydrogen Energy.

[212] Shlyakhtina A.V., Lyskov N.V., Šalkus T., Kežionis A., Patrakeev M.V., Leonidov I.A., Shcherbakova L.G., Chernyak S.A., Shefer K.I., Sadovskaya E.M. (2021). Conductivity and Oxygen Diffusion in Bixbyites and Fluorites Ln_6−x_MoO_12−δ_ (Ln = Er, Tm; x = 0, 0.5). Int. J. Hydrogen Energy.

[213] Bartram S.F. (1966). Crystal Structure of the Rhombohedral MO_3_._3_R_2_O_3_ Compounds (M = U, W, or Mo) and Their Relation to Ordered R_7_O_12_ Phases. Inorg. Chem..

[214] Czeskleba-Kerner H., Cros B., Tourne G. (1981). Phase Equilibria and Compound Formation in the Nd-Mo-O System between 1273 and 1673°K. J. Solid State Chem..

[215] Polfus J.M., Li Z., Xing W., Sunding M.F., Walmsley J.C., Fontaine M.-L., Henriksen P.P., Bredesen R. (2016). Chemical Stability and H_2_ Flux Degradation of Cercer Membranes Based on Lanthanum Tungstate and Lanthanum Chromite. J. Membr. Sci..

[216] Eremeev N.F., Bespalko Y.N., Sadovskaya E.M., Skriabin P.I., Krieger T.A., Ishchenko A.V., Sadykov V.A. (2022). Structural and Transport Properties of Nd Tungstates and Their Composites with Ni_0.5_Cu_0.5_O Obtained by Mechanical Activation. Dalton Trans..

[217] Voronkova V.I., Leonidov I.A., Kharitonova E.P., Belov D.A., Patrakeev M.V., Leonidova O.N., Kozhevnikov V.L. (2014). Oxygen Ion and Electron Conductivity in Fluorite-like Molybdates Nd_5_Mo_3_O_16_ and Pr_5_Mo_3_O_16_. J. Alloys Compd..

[218] Chambrier M.-H. (2009). Analyse Structurale Au Sein Du Diagramme de Phase La_2_O_3_-WO_3_ et Exploration Des Proprietes de Conduction Ionique. Ph.D. Thesis.

[219] Chambrier M.-H., Le Bail A., Giovannelli F., Redjaïmia A., Florian P., Massiot D., Suard E., Goutenoire F. (2014). La_10_W_2_O_21_: An Anion-Deficient Fluorite-Related Superstructure with Oxide Ion Conduction. Inorg. Chem..

[220] Balaguer M., Yoo C.-Y., Bouwmeester H.J.M., Serra J.M. (2013). Bulk Transport and Oxygen Surface Exchange of the Mixed Ionic–Electronic Conductor Ce_1−x_Tb_x_O_2−δ_ (x = 0.1, 0.2, 0.5). J. Mater. Chem. A.

[221] Kumari N., Anjum U., Haider M.A., Basu S. (2019). Oxygen Anion Diffusion in Doped Ceria M_x_Ce_1−x_O_2-0.5x_ (M=Gd, Sm and Pr): A Molecular Dynamics Simulation Study. MRS Adv..

[222] Schaube M., Merkle R., Maier J. (2019). Oxygen Exchange Kinetics on Systematically Doped Ceria: A Pulsed Isotope Exchange Study. J. Mater. Chem. A.

[223] Fernández-García M., Martínez-Arias A., Hanson J.C., Rodriguez J.A. (2004). Nanostructured Oxides in Chemistry: Characterization and Properties. Chem. Rev..

[224] Hungría A.B., Martínez-Arias A., Fernández-García M., Iglesias-Juez A., Guerrero-Ruiz A., Calvino J.J., Conesa J.C., Soria J. (2003). Structural, Morphological, and Oxygen Handling Properties of Nanosized Cerium−Terbium Mixed Oxides Prepared by Microemulsion. Chem. Mater..

[225] Zhong F., Yang S., Chen C., Fang H., Chen K., Zhou C., Lin L., Luo Y., Au C., Jiang L. (2022). Defect-Induced Pyrochlore Pr_2_Zr_2_O_7_ Cathode Rich in Oxygen Vacancies for Direct Ammonia Solid Oxide Fuel Cells. J. Power Sources.

[226] Anantharaman A.P., Dasari H.P. (2021). Potential of Pyrochlore Structure Materials in Solid Oxide Fuel Cell Applications. Ceram. Int..

[227] Julbe A., Farrusseng D., Guizard C. (2005). Limitations and Potentials of Oxygen Transport Dense and Porous Ceramic Membranes for Oxidation Reactions. Catal. Today.

[228] Bespalko Y., Eremeev N., Sadovskaya E., Krieger T., Bulavchenko O., Suprun E., Mikhailenko M., Korobeynikov M., Sadykov V. (2023). Synthesis and Oxygen Mobility of Bismuth Cerates and Titanates with Pyrochlore Structure. Membranes.

[229] Phair J.W., Badwal S.P.S. (2006). Materials for Separation Membranes in Hydrogen and Oxygen Production and Future Power Generation. Sci. Technol. Adv. Mater..

[230] Phair J.W., Badwal S.P.S. (2006). Review of Proton Conductors for Hydrogen Separation. Ionics.

[231] Sadykov V.A., Koroleva M.S., Piir I.V., Chezhina N.V., Korolev D.A., Skriabin P.I., Krasnov A.V., Sadovskaya E.M., Eremeev N.F., Nekipelov S.V. (2018). Structural and Transport Properties of Doped Bismuth Titanates and Niobates. Solid State Ion..

[232] Krasnov A.G., Piir I.V., Koroleva M.S., Sekushin N.A., Ryabkov Y.I., Piskaykina M.M., Sadykov V.A., Sadovskaya E.M., Pelipenko V.V., Eremeev N.F. (2017). The Conductivity and Ionic Transport of Doped Bismuth Titanate Pyrochlore Bi_1.6_M_x_Ti_2_O_7−δ_ (M–Mg, Sc, Cu). Solid State Ion..

[233] Shlyakhtina A.V., Pigalskiy K.S., Belov D.A., Lyskov N.V., Kharitonova E.P., Kolbanev I.V., Borunova A.B., Karyagina O.K., Sadovskaya E.M., Sadykov V.A. (2018). Proton and Oxygen Ion Conductivity in the Pyrochlore/Fluorite Family of Ln_2−x_Ca_x_ScMO_7−δ_ (Ln = La, Sm, Ho, Yb; M = Nb, Ta; x = 0, 0.05, 0.1) Niobates and Tantalates. Dalton Trans..

[234] Shimura T., Komori M., Iwahara H. (1996). Ionic Conduction in Pyrochlore-Type Oxides Containing Rare Earth Elements at High Temperature. Solid State Ion..

[235] Omata T., Ikeda K., Tokashiki R., Otsuka-Yao-Matsuo S. (2004). Proton Solubility for La_2_Zr_2_O_7_ with a Pyrochlore Structure Doped with a Series of Alkaline-Earth Ions. Solid State Ion..

[236] Eurenius K.E.J., Ahlberg E., Knee C.S. (2010). Proton Conductivity in Ln_1.96_Ca_0.04_Sn_2_O_7-δ_ (Ln=La, Sm, Yb) Pyrochlores as a Function of the Lanthanide Size. Solid State Ion..

[237] Eurenius K.E.J., Ahlberg E., Ahmed I., Eriksson S.G., Knee C.S. (2010). Investigation of Proton Conductivity in Sm_1.92_Ca_0.08_Ti_2_O_7−δ_ and Sm_2_Ti_1.92_Y_0.08_O_7−δ_ Pyrochlores. Solid State Ion..

[238] Antonova E.P., Farlenkov A.S., Tropin E.S., Eremin V.A., Khodimchuk A.V., Ananyev M.V. (2017). Oxygen Isotope Exchange, Water Uptake and Electrical Conductivity of Ca-Doped Lanthanum Zirconate. Solid State Ion..

[239] Huo D., Gosset D., Siméone D., Baldinozzi G., Khodja H., Villeroy B., Surblé S. (2015). Influence of Sintering Methods on Microstructure and Ionic Conductivity of La_1.95_Sr_0.05_Zr_2_O_6.975_ Synthesized by Co-Precipitation. Solid State Ion..

[240] Huo D., Baldinozzi G., Siméone D., Khodja H., Surblé S. (2016). Grain Size-Dependent Electrical Properties of La_1.95_Sr_0.05_Zr_2_O_7-δ_ as Potential Proton Ceramic Fuel Cell Electrolyte. Solid State Ion..

[241] Shlyakhtina A.V., Abrantes J.C.C., Gomes E., Lyskov N.V., Konysheva E.Y., Chernyak S.A., Kharitonova E.P., Karyagina O.K., Kolbanev I.V., Shcherbakova L.G. (2019). Evolution of Oxygen–Ion and Proton Conductivity in Ca-Doped Ln_2_Zr_2_O_7_ (Ln = Sm, Gd), Located Near Pyrochlore–Fluorite Phase Boundary. Materials.

[242] Shlyakhtina A.V., Lyskov N.V., Konysheva E.Y., Chernyak S.A., Kolbanev I.V., Vorobieva G.A., Shcherbakova L.G. (2020). Gas-Tight Proton-Conducting Nd_2−x_Ca_x_Zr_2_O_7−δ_ (x = 0, 0.05) Ceramics. J. Solid State Electrochem..

[243] Kaur P., Singh K. (2020). Review of Perovskite-Structure Related Cathode Materials for Solid Oxide Fuel Cells. Ceram. Int..

[244] Ndubuisi A., Abouali S., Singh K., Thangadurai V. (2022). Recent Advances, Practical Challenges, and Perspectives of Intermediate Temperature Solid Oxide Fuel Cell Cathodes. J. Mater. Chem. A.

[245] Farlenkov A.S., Vlasov M.I., Porotnikova N.M., Bobrikov I.A., Khodimchuk A.V., Ananyev M.V. (2020). Hydrogen Diffusivity in the Sr-Doped LaScO_3_ Proton-Conducting Oxides. Int. J. Hydrogen Energy.

[246] Ji Q., Bi L., Zhang J., Cao H., Zhao X.S. (2020). The Role of Oxygen Vacancies of ABO_3_ Perovskite Oxides in the Oxygen Reduction Reaction. Energy Environ. Sci..

[247] Swierczek K., Marzec J., Palubiak D., Zajac W., Molenda J. (2006). LFN and LSCFN Perovskites—Structure and Transport Properties. Solid State Ion..

[248] De Souza R.A., Kilner J.A., Walker J.F. (2000). A SIMS Study of Oxygen Tracer Diffusion and Surface Exchange in La_0.8_Sr_0.2_MnO_3+δ_. Mater. Lett..

[249] Balaguer M., Vert V.B., Navarrete L., Serra J.M. (2013). SOFC Composite Cathodes Based on LSM and Co-Doped Cerias (Ce_0.8_Gd_0.1_X_0.1_O_2–δ_, X = Gd, Cr, Mg, Bi, Ce). J. Power Sources.

[250] Chen M., Cheng Y., He S., Ai N., Veder J.-P., Rickard W.D.A., Saunders M., Chen K., Zhang T., Jiang S.P. (2018). Active, Durable Bismuth Oxide-Manganite Composite Oxygen Electrodes: Interface Formation Induced by Cathodic Polarization. J. Power Sources.

[251] Wang X., Tang B., Wen P., Dong W., Wang L., Wang D. (2022). YSZ/LSM Composite Cathode Deposited by Solution Precursor Plasma Spraying. Coatings.

[252] Budiman R.A., Miyazaki T., Hashimoto S., Yashiro K., Kawada T. (2016). Determination of Oxygen Surface Exchange Constant of LaNi_0.6_Fe_0.4_O_3−δ_ Coated with Ce_0.9_Gd_0.1_O_1.95_ by Isotope Exchange Technique. Solid State Ion..

[253] Chen J., Vashook V., Trots D.M., Wang S., Guth U. (2014). Chemical Diffusion and Oxygen Exchange of LaNi_0.4_Fe_0.6_O_3−δ_ Ceramics. J. Adv. Ceram..

[254] Chen J.Y., Rebello J., Vashook V., Trots D.M., Wang S.R., Wen T.L., Zosel J., Guth U. (2011). Thermal Stability, Oxygen Non-Stoichiometry and Transport Properties of LaNi_0.6_Fe_0.4_O_3_. Solid State Ion..

[255] Kharton V.V., Viskup A.P., Naumovich E.N., Tikhonovich V.N. (1999). Oxygen Permeability of LaFe_1−x_Ni_x_O_3−δ_ Solid Solutions. Mater. Res. Bull..

[256] Harrison C.M., Slater P.R., Steinberger-Wilckens R. (2021). Lanthanum Nickelates and Their Application in Solid Oxide Cells—The LaNi_1−x_Fe_x_O_3_ System and Other ABO_3_-Type Nickelates. Solid State Ion..

[257] Khoshkalam M., Faghihi-Sani M.A., Tong X., Chen M., Hendriksen P.V. (2020). Enhanced Activity of Pr_6_O_11_ and CuO Infiltrated Ce_0.9_Gd_0.1_O_2_ Based Composite Oxygen Electrodes. J. Electrochem. Soc..

[258] Pikalova E., Bogdanovich N., Kolchugin A., Shubin K., Ermakova L., Eremeev N., Farlenkov A., Khrustov A., Filonova E., Sadykov V. (2021). Development of Composite LaNi_0.6_Fe_0.4_O_3-δ_-Based Air Electrodes for Solid Oxide Fuel Cells with a Thin-Film Bilayer Electrolyte. Int. J. Hydrogen Energy.

[259] Hou J., Qian J., Bi L., Gong Z., Peng R., Liu W. (2015). The Effect of Oxygen Transfer Mechanism on the Cathode Performance Based on Proton-Conducting Solid Oxide Fuel Cells. J. Mater. Chem. A.

[260] Pikalova E., Bogdanovich N., Kolchugin A., Ermakova L., Khrustov A., Farlenkov A., Bronin D. (2021). Methods to Increase Electrochemical Activity of Lanthanum Nickelate-Ferrite Electrodes for Intermediate and Low Temperature SOFCs. Int. J. Hydrogen Energy.

[261] Basu R.N., Tietz F., Teller O., Wessel E., Buchkremer H.P., Stöver D. (2003). LaNi_0.6_Fe_0.4_O_3_ as a Cathode Contact Material for Solid Oxide Fuel Cells. J. Solid State Electrochem..

[262] Osinkin D., Bogdanovich N. (2023). Sintering Aid Strategy for Promoting Oxygen Reduction Reaction on High-Performance Double-Layer LaNi_0.6_Fe_0.4_O_3–δ_ Composite Electrode for Devices Based on Solid-State Membranes. Membranes.

[263] Tai L., Nasrallah M., Anderson H., Sparlin D., Sehlin S.-W. (1995). Structure and Electrical Properties of La_1−x_Sr_x_Co_1−y_Fe_y_O_3_. Part 1. The System La_0.8_Sr_0.2_Co_1−y_Fe_y_O_3_. Solid State Ion..

[264] Acosta M., Baiutti F., Tarancón A., MacManus-Driscoll J.L. (2019). Nanostructured Materials and Interfaces for Advanced Ionic Electronic Conducting Oxides. Adv. Mater. Interfaces.

[265] Vibhu V., Yildiz S., Vinke I.C., Eichel R.-A., Bassat J.-M., De Haart L.G.J. (2019). High Performance LSC Infiltrated LSCF Oxygen Electrode for High Temperature Steam Electrolysis Application. J. Electrochem. Soc..

[266] Budiman R.A., Hong H.J., Hashimoto S., Yashiro K., Bagarinao K.D., Kishimoto H., Yamaji K., Kawada T. (2020). Determination of Relevant Factors Affecting the Surface Oxygen Exchange Coefficient of Solid Oxide Fuel Cell Cathode with Ionic Conducting Oxide Coating. Solid State Ion..

[267] Christy M., Rajan H., Yang H., Kim Y.-B. (2020). Optimizing the Surface Characteristics of La_0.6_Sr_0.4_CoO_3−δ_ Perovskite Oxide by Rapid Flash Sintering Technology for Easy Fabrication and Fast Reaction Kinetics in Alkaline Medium. Energy Fuels.

[268] Wolf S.E., Vibhu V., Tröster E., Vinke I.C., Eichel R.-A., De Haart L.G.J. (2022). Steam Electrolysis vs. Co-Electrolysis: Mechanistic Studies of Long-Term Solid Oxide Electrolysis Cells. Energies.

[269] Sadykov V.A., Pavlova S.N., Vinokurov Z.S., Shmakov A.N., Eremeev N.F., Fedorova Y.E., Yakimchuk E.P., Kriventsov V.V., Bolotov V.A., Tanashev Y.Y. (2016). Application of SR Methods for the Study of Nanocomposite Materials for Hydrogen Energy. Phys. Procedia.

[270] Aziz A.J.A., Baharuddin N.A., Somalu M.R., Muchtar A. (2020). Review of Composite Cathodes for Intermediate-Temperature Solid Oxide Fuel Cell Applications. Ceram. Int..

[271] Ren R., Wang Z., Xu C., Sun W., Qiao J., Rooney D.W., Sun K. (2019). Tuning the Defects of the Triple Conducting Oxide BaCo_0.4_Fe_0.4_Zr_0.1_Y_0.1_O_3−δ_ Perovskite toward Enhanced Cathode Activity of Protonic Ceramic Fuel Cells. J. Mater. Chem. A.

[272] Seong A., Kim J., Jeong D., Sengodan S., Liu M., Choi S., Kim G. (2021). Electrokinetic Proton Transport in Triple (H^+^/O^2−^/e^−^) Conducting Oxides as a Key Descriptor for Highly Efficient Protonic Ceramic Fuel Cells. Adv. Sci..

[273] Kasyanova A.V., Tarutina L.R., Rudenko A.O., Lyagaeva J.G., Medvedev D.A. (2020). Ba(Ce,Zr)O_3_ -Based Electrodes for Protonic Ceramic Electrochemical Cells: Towards Highly Compatible Functionality and Triple-Conducting Behaviour. Russ. Chem. Rev..

[274] Uchida H., Yoshikawa H., Esaka T., Ohtsu S., Iwahara H. (1989). Formation of Protons in SrCeO_3_-Based Proton Conducting Oxides. Part II. Evaluation of Proton Concentration and Mobility in Yb-Doped SrCeO_3_. Solid State Ion..

[275] Kreuer K., Schonherr E., Maier J. (1994). Proton and Oxygen Diffusion in BaCeO_3_ Based Compounds: A Combined Thermal Gravimetric Analysis and Conductivity Study. Solid State Ion..

[276] Kreuer K.D., Münch W., Traub U., Maier J. (1998). On Proton Transport in Perovskite-Type Oxides and Plastic Hydroxides. Berichte Der Bunsenges. Für Phys. Chem..

[277] Afroze S., Karim A., Cheok Q., Eriksson S., Azad A.K. (2019). Latest Development of Double Perovskite Electrode Materials for Solid Oxide Fuel Cells: A Review. Front. Energy.

[278] Zhang Y., Shen L., Wang Y., Du Z., Zhang B., Ciucci F., Zhao H. (2022). Correction: Enhanced Oxygen Reduction Kinetics of IT-SOFC Cathode with PrBaCo_2_O_5+δ_/Gd_0.1_Ce_0.9_O_2−δ_ Coherent Interface. J. Mater. Chem. A.

[279] Ivanov I.L., Zakiryanov P.O., Sereda V.V., Mazurin M.O., Malyshkin D.A., Zuev A.Y., Tsvetkov D.S. (2022). Nonstoichiometry, Defect Chemistry and Oxygen Transport in Fe-Doped Layered Double Perovskite Cobaltite PrBaCo_2−x_Fe_x_O_6−δ_ (x = 0–0.6) Membrane Materials. Membranes.

[280] Ananyev M.V., Eremin V.A., Tsvetkov D.S., Porotnikova N.M., Farlenkov A.S., Zuev A.Y., Fetisov A.V., Kurumchin E.K. (2017). Oxygen Isotope Exchange and Diffusion in LnBaCo_2_O_6−δ_ (Ln = Pr, Sm, Gd) with Double Perovskite Structure. Solid State Ion..

[281] Li K., Niemczyk A., Świerczek K., Stępień A., Naumovich Y., Dąbrowa J., Zajusz M., Zheng K., Dabrowski B. (2022). Co-Free Triple Perovskite La_1.5_Ba_1.5_Cu_3_O_7±δ_ as a Promising Air Electrode Material for Solid Oxide Fuel Cells. J. Power Sources.

[282] Lee J.-I., Park K.-Y., Park H., Bae H., Saqib M., Park K., Shin J.-S., Jo M., Kim J., Song S.-J. (2021). Triple Perovskite Structured Nd_1.5_Ba_1.5_CoFeMnO_9−δ_ Oxygen Electrode Materials for Highly Efficient and Stable Reversible Protonic Ceramic Cells. J. Power Sources.

[283] Belik A.A., Johnson R.D., Khalyavin D.D. (2021). The Rich Physics of A-Site-Ordered Quadruple Perovskite Manganites AMn_7_O_12_. Dalton Trans..

[284] Moazzam M., Li C., Cordaro G., Dezanneau G. (2023). Effect of A-Site Cation Ordering on Oxygen Diffusion in NdBa_2_Fe_3_O_8_ through Molecular Dynamics. J. Solid State Chem..

[285] Morales-Zapata M.A., Larrea A., Laguna-Bercero M.A. (2023). Lanthanide Nickelates for Their Application on Solid Oxide Cells. Electrochim. Acta.

[286] Boehm E., Bassat J.-M., Steil M.C., Dordor P., Mauvy F., Grenier J.-C. (2003). Oxygen Transport Properties of La_2_Ni_1−x_Cu_x_O_4+δ_ Mixed Conducting Oxides. Solid State Sci..

[287] Tropin E., Ananyev M., Porotnikova N., Khodimchuk A., Saher S., Farlenkov A., Kurumchin E., Shepel D., Antipov E., Istomin S. (2019). Oxygen Surface Exchange and Diffusion in Pr_1.75_Sr_0.25_Ni_0.75_Co_0.25_O_4±δ_. Phys. Chem. Chem. Phys..

[288] Yatoo M.A., Skinner S.J. (2022). Ruddlesden-Popper Phase Materials for Solid Oxide Fuel Cell Cathodes: A Short Review. Mater. Today Proc..

[289] Pikalov S.M., Vedmid’ L.B., Filonova E.A., Pikalova E.Y., Lyagaeva J.G., Danilov N.A., Murashkina A.A. (2019). High-Temperature Behavior of Calcium Substituted Layered Neodymium Nickelates. J. Alloys Compd..

[290] Tsvinkinberg V.A., Tolkacheva A.S., Filonova E.A., Gyrdasova O.I., Pikalov S.M., Vorotnikov V.A., Vylkov A.I., Moskalenko N.I., Pikalova E.Y. (2021). Structure, Thermal Expansion and Electrical Conductivity of La_2−x_Gd_x_NiO_4+δ_ (0.0 ≤x≤ 0.6) Cathode Materials for SOFC Applications. J. Alloys Compd..

[291] Sadykov V.A., Sadovskaya E.M., Pikalova E.Y., Kolchugin A.A., Filonova E.A., Pikalov S.M., Eremeev N.F., Ishchenko A.V., Lukashevich A.I., Bassat J.M. (2018). Transport Features in Layered Nickelates: Correlation between Structure, Oxygen Diffusion, Electrical and Electrochemical Properties. Ionics.

[292] Sadykov V.A., Sadovskaya E.M., Filonova E.A., Eremeev N.F., Bogdanovich N.M., Pikalov S.M., Vylkov A.I., Pikalova E.Y. (2020). Mixed Ionic-Electronic Conductivity Features of A-Site Deficient Nd Nickelates. Ceram. Int..

[293] Boehm E., Bassat J., Dordor P., Mauvy F., Grenier J., Stevens P. (2005). Oxygen Diffusion and Transport Properties in Non-Stoichiometric Ln_2−x_NiO_4+δ_ Oxides. Solid State Ion..

[294] Minervini L., Grimes R.W., Kilner J.A., Sickafus K.E. (2000). Oxygen Migration in La_2_NiO_4+δ_. J. Mater. Chem..

[295] Chroneos A., Parfitt D., Kilner J.A., Grimes R.W. (2009). A molecular dynamics study of anisotropic oxygen diffusion in La_2_NiO_4+δ_. Open-Access J. Basic Princip. Diff. Theory Exp. Appl..

[296] Li X., Benedek N.A. (2015). Enhancement of Ionic Transport in Complex Oxides through Soft Lattice Modes and Epitaxial Strain. Chem. Mater..

[297] Lee D., Lee H. (2017). Controlling Oxygen Mobility in Ruddlesden–Popper Oxides. Materials.

[298] Xu S., Jacobs R., Morgan D. (2018). Factors Controlling Oxygen Interstitial Diffusion in the Ruddlesden–Popper Oxide La_2–x_Sr_x_NiO_4+δ_. Chem. Mater..

[299] Sadykov V.A., Pikalova E.Y., Vinokurov Z.S., Shmakov A.N., Eremeev N.F., Sadovskaya E.M., Lyagaeva J.G., Medvedev D.A., Belyaev V.D. (2019). Tailoring the Structural, Thermal and Transport Properties of Pr_2_NiO_4+δ_ through Ca-Doping Strategy. Solid State Ion..

[300] Pikalova E.Y., Sadykov V.A., Filonova E.A., Eremeev N.F., Sadovskaya E.M., Pikalov S.M., Bogdanovich N.M., Lyagaeva J.G., Kolchugin A.A., Vedmid’ L.B. (2019). Structure, Oxygen Transport Properties and Electrode Performance of Ca-Substituted Nd_2_NiO_4_. Solid State Ion..

[301] Filonova E.A., Pikalova E.Y., Maksimchuk T.Y., Vylkov A.I., Pikalov S.M., Maignan A. (2021). Crystal Structure and Functional Properties of Nd_1.6_Ca_0.4_Ni_1-y_Cu_y_O_4+δ_ as Prospective Cathode Materials for Intermediate Temperature Solid Oxide Fuel Cells. Int. J. Hydrogen Energy.

[302] Xue J., Liao Q., Chen W., Bouwmeester H.J.M., Wang H., Feldhoff A. (2015). A New CO_2_-Resistant Ruddlesden–Popper Oxide with Superior Oxygen Transport: A-Site Deficient (Pr_0.9_La_0.1_)_1.9_(Ni_0.74_Cu_0.21_Ga_0.05_)O_4+δ_. J. Mater. Chem. A.

[303] Pikalova E., Eremeev N., Sadovskaya E., Sadykov V., Tsvinkinberg V., Pikalova N., Kolchugin A., Vylkov A., Baynov I., Filonova E. (2022). Influence of the Substitution with Rare Earth Elements on the Properties of Layered Lanthanum Nickelate—Part 1: Structure, Oxygen Transport and Electrochemistry Evaluation. Solid State Ion..

[304] Ishihara T., Sirikanda N., Nakashima K., Miyoshi S., Matsumoto H. (2010). Mixed Oxide Ion and Hole Conductivity in Pr_2−α_Ni_0.76−x_Cu_0.24_Ga_x_O_4+δ_ Membrane. J. Electrochem. Soc..

[305] Yashima M., Sirikanda N., Ishihara T. (2010). Crystal Structure, Diffusion Path, and Oxygen Permeability of a Pr_2_NiO_4_ -Based Mixed Conductor (Pr_0.9_La_0.1_)_2_(Ni_0.74_Cu_0.21_Ga_0.05_)O_4+δ_. J. Am. Chem. Soc..

[306] Adler S. (2000). Limitations of Charge-Transfer Models for Mixed-Conducting Oxygen Electrodes. Solid State Ion..

[307] Yashima M., Yamada H., Nuansaeng S., Ishihara T. (2012). Role of Ga^3+^ and Cu^2+^ in the High Interstitial Oxide-Ion Diffusivity of Pr_2_NiO_4_ -Based Oxides: Design Concept of Interstitial Ion Conductors through the Higher-Valence d^10^ Dopant and Jahn–Teller Effect. Chem. Mater..

[308] Maksimchuk T., Filonova E., Mishchenko D., Eremeev N., Sadovskaya E., Bobrikov I., Fetisov A., Pikalova N., Kolchugin A., Shmakov A. (2022). High-Temperature Behavior, Oxygen Transport Properties, and Electrochemical Performance of Cu-Substituted Nd_1.6_Ca_0.4_NiO_4+δ_ Electrode Materials. Appl. Sci..

[309] Sadykov V.A., Sadovskaya E.M., Eremeev N.F., Maksimchuk T.Y., Pikalov S.M., Filonova E.A., Pikalova N.S., Gilev A.R., Pikalova E.Y. (2023). Structure, Oxygen Mobility, and Electrochemical Characteristics of La_1.7_Ca_0.3_Ni_1−x_Cu_x_O_4+δ_ Materials. Russ. J. Electrochem..

[310] Miyoshi S., Furuno T., Sangoanruang O., Matsumoto H., Ishihara T. (2007). Mixed Conductivity and Oxygen Permeability of Doped Pr_2_NiO_4_-Based Oxides. J. Electrochem. Soc..

[311] Song J., Ning D., Boukamp B., Bassat J.-M., Bouwmeester H.J.M. (2020). Structure, Electrical Conductivity and Oxygen Transport Properties of Ruddlesden–Popper Phases Ln_n+1_Ni_n_O_3n+1_ (Ln = La, Pr and Nd; *n* = 1, 2 and 3). J. Mater. Chem. A.

[312] Yatoo M.A., Seymour I.D., Skinner S.J. (2023). Neutron Diffraction and DFT Studies of Oxygen Defect and Transport in Higher-Order Ruddlesden–Popper Phase Materials. RSC Adv..

[313] Ota T., Alaydrus M., Kizaki H., Morikawa Y. (2022). Analysis of Atomic Structure, Magnetic Ordering, and Oxygen Diffusion in Oxygen Deficient Sr_3_Fe_2_O_7−δ_ Perovskite: Toward Rational Catalysts Design. Phys. Rev. Mater..

[314] Yatoo M.A., Skinner S.J. (2023). Oxygen Transport in Higher-Order Ruddlesden-Popper Phase Materials. ECS Trans..

[315] Kuo J.H., Anderson H.U., Sparlin D.M. (1990). Oxidation-Reduction Behavior of Undoped and Sr-Doped LaMnO_3_: Defect Structure, Electrical Conductivity, and Thermoelectric Power. J. Solid State Chem..

[316] Cao J., Su C., Ji Y., Yang G., Shao Z. (2021). Recent Advances and Perspectives of Fluorite and Perovskite-Based Dual-Ion Conducting Solid Oxide Fuel Cells. J. Energy Chem..

[317] Ananyev M.V., Porotnikova N.M., Eremin V.A., Kurumchin E.K. (2021). Interaction of O_2_ with LSM–YSZ Composite Materials and Oxygen Spillover Effect. ACS Catal..

[318] Wang M., Su C., Zhu Z., Wang H., Ge L. (2022). Composite Cathodes for Protonic Ceramic Fuel Cells: Rationales and Materials. Compos. Part B Eng..

[319] Tan K.H., Hamimah A.R., Nor M.R. (2022). Addition of Sm_0.2_Ce_0.8_O_1.9_ carbonate into perovskite Cathode materials for low-temperature solid oxide fuel cell: Short review. J. Innov. Technol..

[320] Esquirol A., Kilner J., Brandon N. (2004). Oxygen Transport in La_0.6_Sr_0.4_Co_0.2_Fe_0.8_O_3-δ_/Ce_0.8_Ge_0.2_O_2−x_ Composite Cathode for IT-SOFCs. Solid State Ion..

[321] Eremeev N.F. (2017). Structural Studies of Pr Nickelate-Cobaltite—Y-Doped Ceria Nanocomposite. J. Ceram. Sci. Tech..

[322] Xin X., Liu L., Liu Y., Zhu Q. (2018). Novel Perovskite-Spinel Composite Conductive Ceramics for SOFC Cathode Contact Layer. Int. J. Hydrogen Energy.

[323] Cheng J., Qian W., Wang P., Tian C. (2022). A High Activity Cathode of Sm0.2Ce_0.8_O_1.9_ Decorated Mn_1.5_Co_1.5_O_4_ Using Ion Impregnation Technique within a Solid Oxide Fuel Cell System. SSRN J..

[324] Wang J., Lu Y., Mushtaq N., Shah M.A.K.Y., Rauf S., Lund P.D., Asghar M.I. (2023). Novel LaFe_2_O_4_ Spinel Structure with a Large Oxygen Reduction Response towards Protonic Ceramic Fuel Cell Cathode. J. Rare Earths.

[325] Ogura Y., Yokoi T., Toyoura K., Matsunaga K. (2020). First-Principles Analysis of Oxide-Ion Conduction Mechanism in Neodymium Silicate. Solid State Ion..

[326] Kendrick E., Kendrick J., Knight K.S., Islam M.S., Slater P.R. (2007). Cooperative Mechanisms of Fast-Ion Conduction in Gallium-Based Oxides with Tetrahedral Moieties. Nat. Mater..

[327] Thabet K., Salle A.L.G.L., Quarez E., Joubert O. (2020). Protonic-Based Ceramics for Fuel Cells and Electrolyzers. Solid Oxide-Based Electrochemical Devices.

[328] Mitra C., Meyer T., Lee H.N., Reboredo F.A. (2014). Oxygen Diffusion Pathways in Brownmillerite SrCoO_2.5_: Influence of Structure and Chemical Potential. J. Chem. Phys..

[329] Zhang W., Yashima M. (2023). Recent Developments in Oxide Ion Conductors: Focusing on Dion–Jacobson Phases. Chem. Commun..

[330] Thangadurai V., Weppner W. (2002). Determination of the Sodium Ion Transference Number of the Dion−Jacobson-Type Layered Perovskite NaCa_2_Nb_3_O_10_ Using Ac Impedance and Dc Methods. Chem. Mater..

[331] Shi J., Han C., Niu H., Zhu Y., Yun S. (2021). Theoretical Investigation of Proton Diffusion in Dion–Jacobson Layered Perovskite RbBiNb_2_O_7_. Nanomaterials.

[332] Teusner M., De Souza R.A., Krause H., Ebbinghaus S.G., Martin M. (2016). Oxygen Transport in Undoped and Doped Mayenite. Solid State Ion..

[333] Phaneuf V.N. (2021). Synthesis, Characterization and Application of Mayenite. Ph.D. Thesis.

[334] Orera A., Slater P.R. (2010). Water Incorporation Studies in Apatite-Type Rare Earth Silicates/Germanates. Solid State Ion..

[335] Yu Z., Liu Q., Ragipani R., Wang B. (2020). Formation and Transport Mechanisms of Hydrogenous Species in Mayenite. J. Phys. Chem. C.

[336] Zhou Q., Wang Y., Bu F., Yang F., Wang M., Li Y. (2021). Preparation and Properties of Low Thermal Expansion Coefficient (Y_0.5_Ca_0.5_)_1−x_In_x_BaCo_3_ZnO_7+δ_ (X=0, 0.1, 0.2, 0.3) Solid Oxide Fuel Cell Cathode Materials. SSRN J..

[337] Shin J.-S., Park H., Park K., Saqib M., Jo M., Kim J.H., Lim H.-T., Kim M., Kim J., Park J.-Y. (2021). Activity of Layered Swedenborgite Structured Y_0.8_Er_0.2_BaCo_3.2_Ga_0.8_O_7+δ_ for Oxygen Electrode Reactions in at Intermediate Temperature Reversible Ceramic Cells. J. Mater. Chem. A.

[338] Kirichenko V.G., Kovalenko O.V. (2014). Study of Element Composition and Component Diffusion of Yttrium Iron Garnet Thin Films. Vopr. At. Nauk. I Tekhnik.

[339] Zhang X., Tian Y., Nie Z., Wu X., Li Y., Ding L. (2022). Electrochemical Characteristics of Ca_3_Co_4_O_9+δ_ Oxygen Electrode for Reversible Solid Oxide Cells. J. Electroanal. Chem..

[340] Urusova A., Bryuzgina A., Solomakhina E., Kolchugin A., Malyshkin D., Pikalova E., Filonova E. (2023). Assessment of the Y-Doped Ca_3_Co_4_O_9+δ_ as Cathode Material for Proton-Conducting Fuel Cells. Int. J. Hydrogen Energy.

[341] Yue X., Huang X., Li J., Su C., Zhang Y., Zhang Y., Wei B., Lv Z. (2022). In-Situ Surface Reconstruction Induced-Significant Performance Promotion of Ca_3_Co_4_O_9+δ_ Cathode for Solid Oxide Fuel Cells. J. Power Sources.

[342] Zhu X., Lusi A., Zhu C., Wang Y., Jin J. (2017). Performance Evaluation of Ca_3_Co_4_O_9-δ_ Cathode on Sm_0.075_Nd_0.075_Ce_0.85_O_2-δ_ Electrolyte for Solid Oxide Fuel Cells. J. Alloys Compd..

[343] Pikalova E., Kolchugin A., Koroleva M., Vdovin G., Farlenkov A., Medvedev D. (2019). Functionality of an Oxygen Ca_3_Co_4_O_9+δ_ Electrode for Reversible Solid Oxide Electrochemical Cells Based on Proton-Conducting Electrolytes. J. Power Sources.

[344] Rolle A., Mohamed H.A.A., Huo D., Capoen E., Mentré O., Vannier R.-N., Daviero-Minaud S., Boukamp B.A. (2016). Ca_3_Co_4_O_9+δ_, a Growing Potential SOFC Cathode Material: Impact of the Layer Composition and Thickness on the Electrochemical Properties. Solid State Ion..

[345] Zhu Y., Zhou W., Chen Y., Shao Z. (2016). An Aurivillius Oxide Based Cathode with Excellent CO_2_ Tolerance for Intermediate-Temperature Solid Oxide Fuel Cells. Angew. Chem. Int. Ed..

[346] Villegas M., Moure C., Fernandez J.F., Duran P. (1996). Low-Temperature Sintering of Submicronic Randomly Oriented Bi_4_Ti_3_O_12_ Materials. Ceram. Int..

[347] Thoréton V., Hu Y., Pirovano C., Capoen E., Nuns N., Mamede A.S., Dezanneau G., Yoo C.Y., Bouwmeester H.J.M., Vannier R.N. (2014). Oxygen Transport Kinetics of the Misfit Layered Oxide Ca_3_Co_4_O_9+δ_. J. Mater. Chem. A.

[348] Filonova E.A., Tokareva E.S., Pikalova N.S., Vylkov A.I., Bogdanovich N.M., Pikalova E.Y. (2020). Assessment of Prospective Cathodes Based on (1−x)Ca_3_Co_4_O_9+δ_-xBaCe_0.5_Zr_0.3_Y_0.1_Yb_0.1_O_3-δ_ Composites for Protonic Ceramic Electrochemical Cells. J. Solid State Electrochem..

[349] Boukamp B.A., Rolle A., Vannier R.N., Sharma R.K., Djurado E. (2020). Electrostatic Spray Deposited Ca_3_Co_4_O_9+δ_ and Ca_3_Co_4_O_9+δ_/Ce_0.9_Gd_0.1_O_1.95_ Cathodes for SOFC. Electrochim. Acta.

[350] Yurchenko M.V., Antonova E.P., Tropin E.S., Suntsov A.Y. (2023). Adjusting Electrochemical Properties of PrBaCo_2_O_6–δ_ as SOFC Cathode by Controllable Ca_3_Co_4_O_9_ Additions. Ceram. Int..

[351] Xing W., Syvertsen G.E., Grande T., Li Z., Haugsrud R. (2012). Hydrogen Permeation, Transport Properties and Microstructure of Ca-Doped LaNbO_4_ and LaNb_3_O_9_ Composites. J. Membr. Sci..

[352] Syvertsen G.E., Estournès C., Fjeld H., Haugsrud R., Einarsrud M.-A., Grande T. (2012). Spark Plasma Sintering and Hot Pressing of Hetero-Doped LaNbO_4_. J. Am. Ceram. Soc..

[353] Haugsrud R., Norby T. (2006). Proton Conduction in Rare-Earth Ortho-Niobates and Ortho-Tantalates. Nat. Mater..

[354] Wood J.R. (2007). Defects and Conductivity in Sr-Doped LaNb_3_O_9_. Master’s Thesis.

[355] Haugsrud R., Norby T. (2006). High-Temperature Proton Conductivity in Acceptor-Doped LaNbO_4_. Solid State Ion..

[356] Sadykov V.A., Bespalko Y.N., Krasnov A.V., Skriabin P.I., Lukashevich A.I., Fedorova Y.E., Sadovskaya E.M., Eremeev N.F., Krieger T.A., Ishchenko A.V. (2018). Novel Proton-Conducting Nanocomposites for Hydrogen Separation Membranes. Solid State Ion..

[357] Altynbekova D., Bespalko Y., Valeev K., Eremeev N., Sadovskaya E., Krieger T., Ulihin A., Uhina A., Massalimova B., Simonov M. (2022). Simple Approach to the Fabrication of Lanthanum Orthoniobates and Nanocomposites with Ni, Cu, and Co Metal Nanoparticles Using Supercritical Isopropanol. J. Compos. Sci..

[358] Colognesi D., Demmel F., Filabozzi A., Pietropaolo A., Pozio A., Romanelli G., Santucci A., Tosti S. (2020). Proton Dynamics in Palladium–Silver: An Inelastic Neutron Scattering Investigation. Molecules.

[359] Huang F., Li X., Shan X., Guo J., Gallucci F., Annaland M.V.S., Liu D. (2020). Hydrogen Transport through the V-Cr-Al Alloys: Hydrogen Solution, Permeation and Thermal-Stability. Separ. Purif. Technol..

[360] Sidorov N.I., Estemirova S.K., Kurbanova E.D., Polukhin V.A. (2022). Hydrogen Kinetics in Membrane Alloys Based on Fe–Ni, Nb–Ni, and V–Ni. Russ. Met. Met..

[361] Alimov V.N., Bobylev I.V., Busnyuk A.O., Kolgatin S.N., Peredistov E.Y., Livshits A.I. (2020). Fuel Processor with Vanadium Alloy Membranes for Converting CH_4_ into Ultrapure Hydrogen to Generate Electricity via Fuel Cell. Appl. Energy.

